# Promising approaches for the assembly of the catalytically active, recombinant *Desulfomicrobium baculatum* hydrogenase with substitutions at the active site

**DOI:** 10.1186/s12934-023-02127-w

**Published:** 2023-07-21

**Authors:** Malgorzata Witkowska, Robert P. Jedrzejczak, Andrzej Joachimiak, Onur Cavdar, Anna Malankowska, Piotr M. Skowron, Agnieszka Zylicz-Stachula

**Affiliations:** 1 Department of Molecular Biotechnology, Faculty of Chemistry, University of Gdansk, Wita Stwosza 63, Gdansk, 80-308 Poland; 2 Structural Biology Center, X-ray Science Division, Argonne National Laboratory, Argonne, IL 60439 USA; 3 Department of Environmental Technology, Faculty of Chemistry, University of Gdansk, Wita Stwosza 63, Gdansk, 80-308 Poland

**Keywords:** Recombinant hydrogenase, Maturase, Metalloenzyme, Biohydrogen, Mutagenesis

## Abstract

**Background:**

Hydrogenases (H2ases) are metalloenzymes capable of the reversible conversion of protons and electrons to molecular hydrogen. Exploiting the unique enzymatic activity of H2ases can lead to advancements in the process of biohydrogen evolution and green energy production.

**Results:**

Here we created of a functional, optimized operon for rapid and robust production of recombinant [NiFe] *Desulfomicrobium baculatum* hydrogenase (Dmb H2ase). The conversion of the [NiFeSe] Dmb H2ase to [NiFe] type was performed on genetic level by site-directed mutagenesis. The native *dmb* operon includes two structural H2ase genes, coding for large and small subunits, and an additional gene, encoding a specific maturase (protease) that is essential for the proper maturation of the enzyme. Dmb, like all H2ases, needs intricate bio-production machinery to incorporate its crucial inorganic ligands and cofactors. Strictly anaerobic, sulfate reducer *D. baculatum* bacteria are distinct, in terms of their biology, from *E. coli*. Thus, we introduced a series of alterations within the native *dmb* genes. As a result, more than 100 elements, further compiled into 32 operon variants, were constructed. The initial requirement for a specific maturase was omitted by the artificial truncation of the large Dmb subunit. The assembly of the produced H2ase subunit variants was investigated both, in vitro and in vivo. This approach resulted in 4 recombinant [NiFe] Dmb enzyme variants, capable of H_2_ evolution. The aim of this study was to overcome the gene expression, protein biosynthesis, maturation and ligand loading bottlenecks for the easy, fast, and cost-effective delivery of recombinant [NiFe] H2ase, using a commonly available *E. coli* strains.

**Conclusion:**

The optimized genetic constructs together with the developed growth and purification procedures appear to be a promising platform for further studies toward fully-active and O_2_ tolerant, recombinant [NiFeSe] Dmb H2ase, resembling the native Dmb enzyme. It could likely be achieved by selective cysteine to selenocysteine substitution within the active site of the [NiFe] Dmb variant.

**Supplementary Information:**

The online version contains supplementary material available at 10.1186/s12934-023-02127-w.

## Introduction

Hydrogen (H_2_) is considered an excellent, environmentally friendly energy carrier. The combustion of H_2_ produces only heat and water vapour, contrary to traditional fossil fuels. Thus, it is gaining more and more attention as an alternative to oil and gas energy [1–3]. The industrial production of hydrogen is aided mostly with the intensive use of chemicals, toxic metals, and a vast amount of energy to maintain specified conditions. At the same time, H_2_, as the most abundant element in the Universe, is widely exploited by living organisms in their energy conservation pathways [4].

Hydrogenases (H2ases) are essential enzymes for the catalytic turnover of hydrogen, which are widely spread in a variety of organisms. These complex metalloenzymes catalyse the uptake and evolution of H_2_. They are divided into several subclasses according to the metal content of the catalytic centre, as well as their phylogenetic and genetic features: (i) [Fe] hydrogenases, (ii) [FeFe] hydrogenases, and (iii) [NiFe] hydrogenases [5].

From the perspective of industrial applications, [NiFe] H2ases could be particularly useful because they exhibit a high activity towards hydrogen evolution and are somewhat resistant to oxygen inactivation. [NiFe] H2ases consist of at least two subunits. The large subunit (LH) of the [NiFe] H2ases contains an active site with nickel and iron atoms, and a small subunit (SH) provides an electron transfer pathway with several, cubane [4Fe4S] or non cubane (like [4Fe3S] or [3Fe4S]) iron-sulphur clusters [6]. In the catalytic centre, nickel and iron coordinate CO and CN^−^ ligands that are rarely found in living organisms [6, 7]. Thus far, only a limited number of the successful, homologous or heterologous production of recombinant [NiFe] H2ases have been reported [6, 8–10], due to their complex structures and maturation processes.

Among [NiFe] H2ases, the specific [NiFeSe] subgroup is of a great interest. H2ases belonging to this subgroup contain selenocysteine (symbol SeCys or U) instead of one of the cysteines (symbol Cys or C) at the active site. [NiFeSe] H2ases, such as *D. baculatum* periplasmic hydrogenase (Dmb H2ase), exhibit an enhanced oxygen tolerance and high H_2_ evolution activity [11, 12]. It has been proposed that the role of selenium might be crucial due to its redox properties of Dmb H2ase, when compared with sulphur [13]. The role of selenium in the catalytic center was examined by Marques et al. by creating a selenocysteine to cysteine variant of *Desulfovibrio vulgaris* Hildenborough (DvH) H2ase. The recombinant enzyme was biosynthesized in a native organism and demonstrated lowered rates of nickel incorporation, which indicates that the selenium moiety plays a role in the maturation process. Nevertheless, this recombinant enzyme was still able to manifest some activity and inactive states characteristic of the [NiFe] type H2ase [14]. Recently, Evans et al. confirmed the importance of a special position of selenocysteine of the [NiFe] Hyd-1 *E. coli* H2ase for catalysis and O_2_ tolerance. The studies were performed using a recent method for UAG-programmed site-specific Sec incorporation [15].

In *E. coli*, site-specific incorporation of SeCys into the biosynthesized polypeptide demands a dedicated set of enzymes, as well as the SeCys insertion sequence (SECIS element) – a strictly defined structure of translated mRNA template [16]. Unfortunately, incorporation of SECIS, while preserving the original DNA sequence, is not always possible. Even if the SECIS component is successfully introduced, obtaining the recombinant protein with positions fully occupied by SeCys is still highly challenging. Additionally, the mechanism behind the incorporation of iron and nickel atoms into the buried active site is complex and not entirely understood, with several proteins known to be essential for this process [17, 18]. However, this should not be a drawback for the heterological expression of the [NiFeSe] H2ase genes in *E. coli*, since *E. coli* itself produces several H2ases and harbors a sufficient set of helper and accessory proteins. The maturation process of LH is finished with the cleavage of the C-terminal extension by a maturation protease, which is specific to a particular H2ase [19]. Only after the LH cleavage occurs, SH can be assembled into functional complex [20]. Maturation of SH involves the synthesis and incorporation of cubane [4Fe4S] clusters. Two major systems – Isc and Suf – are responsible for Fe-S cluster biogenesis [21–23]. Their role is supposed to be partially interchangeable. It was shown, however, that widely used *E. coli* strains carry an in-frame gene fusion in the *suf* operon, impairing the efficiency of the cluster synthesis [24].

Although native Dmb H2ase is secreted to periplasm, a specific DNA fragment, coding for a membrane translocation signal is present only in the SH coding gene. Interestingly, such a signal sequence directs the protein transport through the twin-arginine translocation (TAT) system, which allows for the secretion of fully folded proteins [25]. We hypothesize that the leader sequence of SH may serve for transportation of both assembled Dmb H2ase subunits.

Thus far, Dmb H2ase was only isolated from *D. baculatum* [26, 27]. Here, we investigated several possible strategies to solve the problems in heterologous H2ase production, arising from the existing differences between a native host - *D. baculatum* and *E. coli*. Our approach resulted in dozens of the tested gene layouts. In this report, we describe a development of a promising strategy for the cloning, heterologous expression, and purification of the catalytically active Dmb H2ase in a fast and simple manner.

## Materials and methods

### Plasmids and bacterial strains

All expression vectors used in this study were developed at the Midwest Center for Structural Genomics (MCSG). The description of the vectors is provided in Supplementary Material File 1. The combination of vectors gives flexible options for tag location and levels of protein expression while allowing for co-expression from two compatible origins of replication without time consuming artificial operon construction. The pMCSG53 expression vector generates a hexa-histidine tag upstream of a TEV recognition site at the N-termini of recombinant protein; this vector also harbors two genes coding for rare tRNAs - AGG/AGA for arginine and AUA for isoleucine [28, 29]. pRSF1 is used for the biosynthesis of protein variants without tags; its origin of replication - RSF1030 - enables the implementation of a two-plasmid system with ColE1 origin vectors. Plasmid pMCSG92 has similar properties to pMCSG53, but the protein of interest (POI) is biosynthesized with a TEV cleavage site and His_6_-tag on the C-termini. pMCSG93 shares most of the pRSF1 functional features, but the TEV cleavage site preceding the His_6_-tag is introduced at the C-terminus of the POI. Many of these vectors share the POIs adjacent sequence, which allows the same set of primers to be used for the cloning of the same insert into all the expression vectors. For a high throughput approach, ligation-independent cloning (LIC) was employed [30]. *E. coli* BL21-Gold(DE3) {F^–^*ompT hsdS*(r_B_^–^ m_B_^–^) *dcm*^+^ Tet^r^*gal* λ(DE3) *end*A Hte} (Agilent Technologies) was used for the cloning procedures and for gene expression experiments. Cells were grown in liquid or on solid LB medium with 150 µg/ml ampicillin and 100 µg/ml kanamycin, when appropriate.

### Bioinformatics analysis, primer design, cloning methods

For the minimal *dmb* operon, three native genes from *D. baculatum* (Gene Bank Accession no. CP001629.1) were selected coding for: (i) large subunit (LH), (ii) small subunit (SH), and (iii) H2ase maturation protease (Hmp). The latter is vital for the final steps of enzyme maturation. For the prediction of transmembrane domains and signal sequences, online tools were used: Phobius [31] and PRED-TAT [32]. Gene sequences were codon optimized for *E. coli* as an expression host (see Supplementary Material File 2 for alignment of the native and optimized gene sequences). For the preparation of the designed recombinant constructs, the PCR cloning method and KOD Hot Start Polymerase (Novagen) were used. For the designing of the primers, a publicly available software tool, provided by the MCSG, was used [33]. PCR was conducted following the touch-down method (TD-PCR) guidelines [34]. DNA amplification schedule is provided in Supplementary Material File 1. PCR products were analyzed by agarose gel electrophoresis. The selected, specific PCR fragments were purified from excess deoxynucleoside triphosphates (dNTPs) using Qiagen spin columns. Subsequently, inserts were treated with T4 DNA polymerase (NEB) and the corresponding dNTP. The prepared DNA inserts were annealed to the T4 DNA polymerase treated vectors and directly transformed into chemically competent *E. coli* cells.

### Screening for positive bacterial clones and POIs biosynthesis

A single bacterial colony was picked from each transformation well and used as an inoculum for 1 ml of LB media with the appropriate antibiotic. Bacterial cells were grown at 37 °C with intense aeration. The next day, 1 ml of fresh LB medium was inoculated with the overnight culture and grown until OD_600_ reached 1. Culture plates were chilled on ice to 19 °C and recombinant gene expression was induced with 1 mM IPTG. Upon induction, the following supplements were added: 1 mM MgCl_2_, 50 µM NiSO_4_, 25 µM FeCl_3_. Cells were further cultured overnight at 18 °C with intense aeration (aerobic conditions) or, where indicated, purged with argon gas for 20 min and grown without shaking (anaerobic conditions). After a preliminary analysis, plasmid DNAs isolated from the selected bacterial clones were subjected to DNA sequencing. Protein expression was analyzed using polyacrylamide gel electrophoresis in denaturing conditions (SDS-PAGE) using Criterion TGX gels (Bio-Rad).

### Screening for POIs solubility

Plates with the induced, overnight cultures were spun down, and the pellets were resuspended in 200 µl of lysis buffer (50 mM HEPES pH 8.0, 500 mM NaCl, 5% glycerol). The resulting suspension was frozen and sonicated in an ice bath in a cup-horn sonicator. Total protein samples were collected, and the plates were spun down for 60 min. Supernatants were mixed with Ni Sepharose (Ge Healthcare) and transferred to a 96-well filter plates. The resin was washed three times with 250 µl of lysis buffer with 20 mM imidazole and finally 40 µl of elution buffer (lysis buffer with 500 mM imidazole) was added to each well. The collected samples were analyzed by SDS-PAGE using Criterion TGX gels (Bio-Rad). If a detectable band of the correct molecular weight was observed after Coomassie-staining, the corresponding POI was scored for expression and solubility.

### Spectrophotometric assay for hydrogenase enzymatic activity

The colorimetric assay of purified hydrogenase was conducted as based on the rate of the diametrical change in colour and extinction coefficient (εMV) of methyl viologen (MV, Sigma) upon oxidation and measured at 600 nm [35, 36]. When MV is in its reduced form (MV ^·+^), its extinction coefficient is ε = 8.25 (λ = 600 nm) and the solution color is dark blue, while the oxidized form (MV ^2+^), the extinction coefficient is ε = 0.0 (λ = 600 nm) and the solution is colorless. This method allows the indirect evaluation of hydrogenase activity as proton reduction is coupled to electron donor oxidation. Samples of purified enzyme were subjected to buffer exchange with Amicon centrifuge filters (Merck Millipore). 12 ml samples were concentrated twice to 0.5 ml and diluted with 50 mM phosphate buffer pH 7.0. After the second concentration, samples were diluted to a final concentration of 3 mg of protein/ml. 2 mM MV solution in 50 mM sodium phosphate pH 7.0 (reduced with 40 mM sodium dithionite) was prepared, and purged under argon for 15 min. 300 ng of protein (100 µl) was mixed with 100 µl of the MV solution, in 96-well clear bottom, black sided polystyrene microplate. The microplate was placed within a pre-cut tedlar bag, heat sealed, purged under argon for 15 min, and transferred to an anaerobic chamber, in which the measurements were conducted simultaneously with a Gene5 Microplate Reader (BioTek). Samples were prepared in triplicate and measurements were undertaken with shaking during a 6 min kinetic run with a measurement interval of 1 min. Control samples were prepared without sodium dithionite and without protein. A change in the absorbance during the time course of the test was calculated (∆A600nm/∆t). The H2ase activity towards hydrogen evolution (U) was calculated as µmoles H_2_/min/mg of enzyme. The calculation was as follows:

$$U\, = \,\frac{{\left( {dilution\,factor} \right)\left( {\frac{{\Delta {\rm{A600nm}}}}{{\Delta t}}} \right)}}{{\left( {{\rm{\varepsilon 600nm}}\, \times \,l} \right)\left( {{\rm{mg}}\,{\rm{of}}\,{\rm{enzyme}}\,{\rm{used}}} \right)}}$$

### Photocatalytic hydrogen evolution assay

7.5 ml phosphate buffer was transferred to the Teflon reactor equipped with a quartz glass and cooling jacket and stirred with magnetic stirrer. The mixture was deaerated to remove the oxygen using nitrogen gas flow for 30 min. Finally, the pre-prepared syringe containing 7.5 ml mixture containing methyl viologen (MV^2+^), L-cysteine and hydrogenase in an anaerobic tent was injected slowly into the reactor under nitrogen flow and purged for another 30 min. The final 15 ml reaction mixture with MV^2+^ = 1.8 mM, L-cysteine = 18 mM and hydrogenase in 50 mM phosphate pH 7 buffer was then irradiated using a 1000 W Xenon lamp (Oriel, 66,021), which emitted both UV and visible irradiation. UV light was removed by a cut-off filter GG420 (λ > 420 nm). The temperature of the reactor was kept at 10^o^C by a thermostat. The zero sample was taken before light irradiation to detect the oxygen level in the reactor, which was always trace amount. At the end of 8 h of irradiation, 200 ml of gas samples were collected from the headspace of the photoreactor using an air-tight syringe (Hamilton) and injected to the gas chromatograph (Thermo Scientific TRACE 1300-GC), coupled with thermal conductivity detector (TCD). The amount of hydrogen was calculated µL per liter of the reaction mixture.

## Results

### Construction of the components of the synthetic *dmb* operon

To obtain layouts containing all three genes of the minimal *dmb* operon, several variants of three synthetic genes encoding LH, SH, and maturation protease (*lh*, *sh*, *hmp* genes respectively) were designed and synthesized (Supplementary material File 3). Every gene of the minimal *dmb* operon was amplified with suitable primers in order to be inserted into the matching vector (pMCSG53, pRSF1, pMCSG92, pMCSG93) or as a subsequent synthetic operon. All primers used in this study are provided in Supplementary Material File 1.

**Fig. 1:**
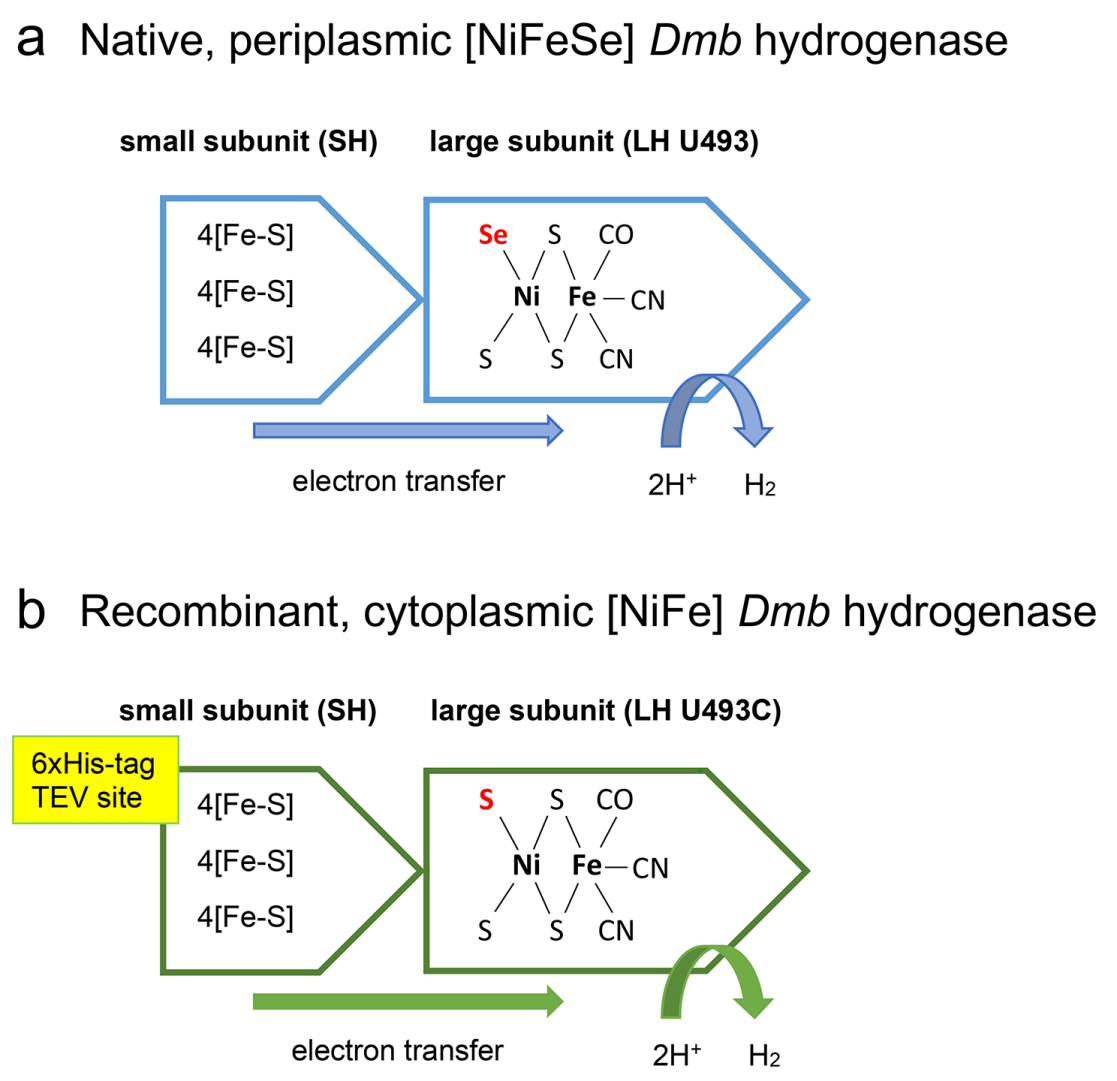
Differences between the native and final, recombinant Dmb hydrogenase. **(a)** Schematic representation of the most prominent features of the native Dmb H2ase. Selenium in the active centre is marked in red. **(b)** Schematic representation of the most prominent features of the recombinant Dmb H2ase. The residues/amino acid position altered by mutagenesis (U493C) is marked in red. The N-terminal His_6_-tag and TEV site, resulting from the expression from pMCSG53 vector, are shown in a yellow rectangle

Native Dmb enzyme belongs to the [NiFeSe] subgroup of H2ases and contains SeCys in the active site. In *E. coli*, the insertion of the SeCys is driven by the appearance of the TGA (STOP) codon and requires the presence of the SECIS element. Unfortunately, the SECIS element for LH coding gene could not be recreated without altering the LH amino acid sequence. PCR reactions aiming to restore the SECIS element structure resulted in products highly ‘toxic’ for *E. coli* host and no expression of the recombinant genes was observed. To deal with this problem, we designed three different LH species: (a) U493M variant with SeCys replaced by methionine residue, (b) U493C variant with SeCys replaced by the Cys residue, as found in [NiFe] H2ases (Fig. 1), and (c) U493STOP variant with a STOP codon inserted at position 493, resembling the native LH gene sequence, but without the presence of SECIS. Initially, the synthetic gene was designed as the U493M variant to obtain a full-length LH polipeptide (514 residues). Later, the gene was subjected to site-directed mutagenesis, and subsequently the U493C and U493STOP variants were obtained.

The performed expression experiments revealed that, in the case of all the investigated SH recombinant constructs, the solubility of the resulting SH protein variants was very low, regardless of the applied operon design and culture medium supplementation (Supplementary Material File 3). For that reason, two methods aimed at improving the solubility of the SH protein were applied: solubilization with urea and in vitro Fe-S cluster insertion [37]. The first of these methods was disruptive to the susceptible structure of cubane clusters, the second one was supposed to have low performance. However, the conducted experiments allowed for an improvement in the solubility of the selected SH variants (see Supplementary Material File 4), which could be further used for the in vitro assembly of recombinant H2ase.

### Dmb H2ase production via two-gene synthetic operon

Our original goal was to produce the active Dmb H2ase using a three-gene synthetic operon. However, in the first step, we constructed and investigated properties of two-gene operons (Supplementary Material File 3, Sect. 1.2, Suppl. Table 2). For this purpose, two types of the ribosome binding site (RBS) were employed. Protein expression analysis of the several constructs showed low levels of the second gene expression. Moreover, it was observed that LH was not being cleaved, regardless of the chosen gene layout. Neither expression of the two-gene operon with both *lh* and *hmp* genes, nor co-expression of the genes using the separate recombinant constructs, resulted in the expected LH cleavage. Without the proper removal of the C-terminal section of the LH polypeptide, the subunit cannot form a heterodimer with SH. This step assures that SH subunit would not be assembled with an inactive LH subunit structure and this is common throughout maturation schemes of phylogenetically distant H2ases [38–40]. To address this problem, we decided to construct their truncated versions (Supplementary Material File 3). For each variant of LH (differing at position 493) we created its shorter counterpart (499 residues in length), with a C-terminal STOP codon (TAA). Employing the truncated LH-coding gene variants allowed us to remove the *hmp* gene from the operons (Supplementary Material File 3: Sect. 1.2). Such an approach eliminated the need to sustain overproduction of three proteins in the bacteria. The resulting constructs contained only two structural *dmb* genes (*lh* and *sh*). However, it was not clear whether the truncated LH species would be assembly competent with SH, as LH cleavage by Hmp is believed to be the final step in the [NiFe]-H2ases maturation. To develop a fast and effective Dmb H2ase purification procedure, we finally selected pMCSG53 as the expression vector (Supplementary Material File 1). Considering the above observed low level of SH solubility, in vitro assembly of the truncated LH and the solubilized SH variant was performed, similarly to the method developed by Caserta et al. [41], who demonstrated that individually purified subunits of the *Ralstonia eutropha* [NiFe] H2ase could be assembled in vitro, forming a fully active enzyme. For in vitro assembly experiments, the separate recombinant bacterial cultures, producing LH variants with or without His_6_-tag and SH variants with His_6_-tag, were grown simultaneously. SH solubilization was performed and the resulting preparations were mixed in a 1:1 ratio prior to Immobilized Metal Affinity Chromatography (IMAC) purification.

### Recombinant gene expression and purification of H2ase

**Fig. 2:**
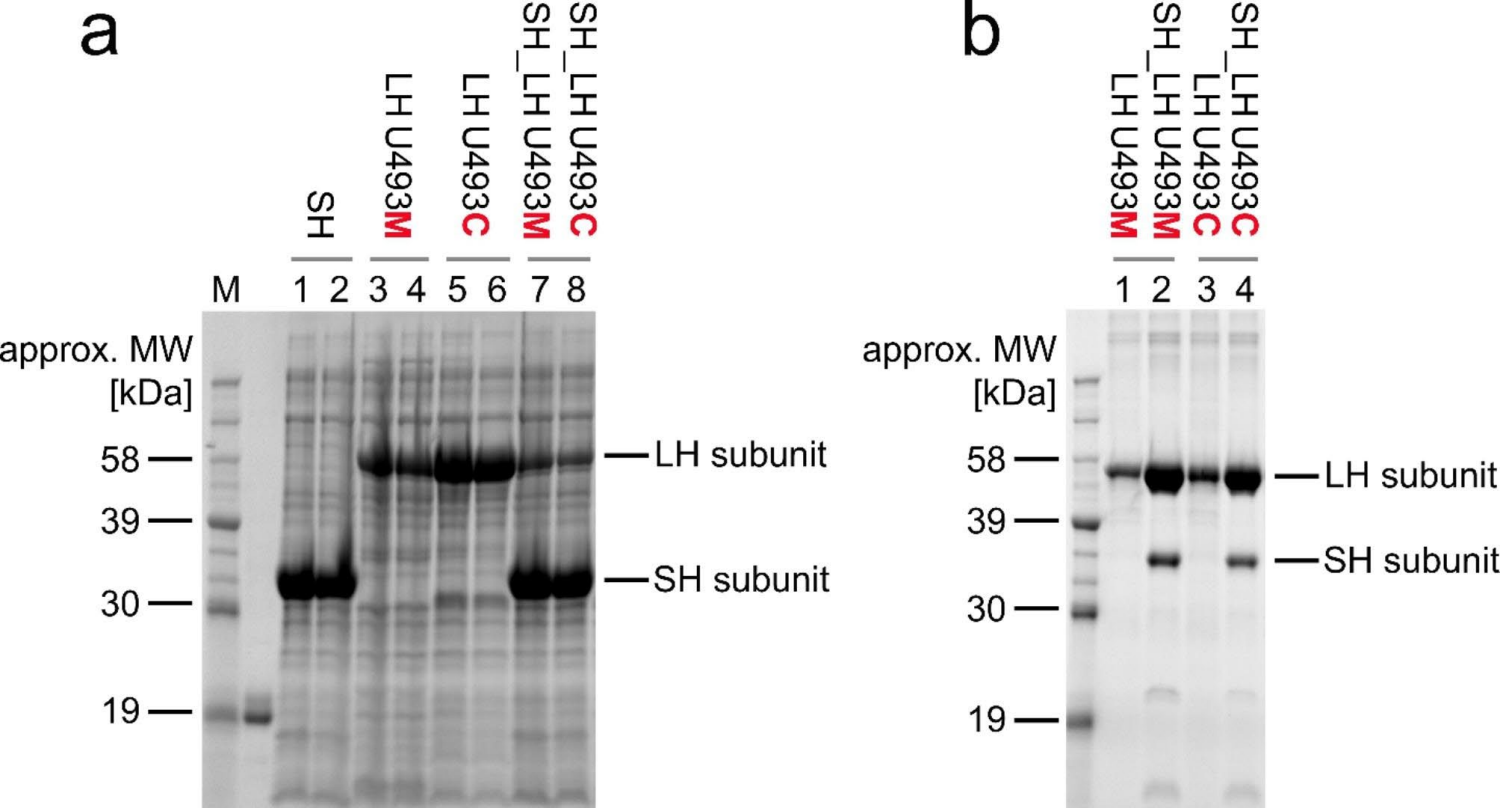
Biosynthesis and isolation of the recombinant Dmb hydrogenase subunits from *E. coli*. After IPTG induction of the recombinant genes, the bacteria were further cultivated overnight at 18^o^C; approx. MW – approximate, theoretically predicted molecular weight. **(a)** Biosynthesis of the large (LH) and small (SH) recombinant Dmb H2ase subunits in *E. coli* BL21(DE3)Gold. Lane M, protein molecular weight ladder, selected bands marked; lanes 1–2, *E. coli* BL21(DE3)Gold [SH_ pMCSG53]; lanes 3–4, *E. coli* BL21(DE3)Gold [LH U493M_ pMCSG53], lanes 5–6, *E. coli* BL21(DE3)Gold [LH U493C_ pMCSG53]; lane 7, *E. coli* BL21(DE3)Gold [SH_LH U493M_ pMCSG53]; lane 8, *E. coli* BL21(DE3)Gold [SH_LH U493C pMCSG53]. **(b)** Isolation and Immobilized metal affinity chromatography (IMAC) purification of the recombinant Dmb H2ase subunits from *E. coli* BL21(DE3)Gold. All the purified protein variants were obtained using the recombinant pMCSG53 constructs. Lane M, protein molecular weight ladder, selected bands marked; lane 1, purified LH U493M; lane 2, purified SH and truncated LH U493M – expression from the operon; lane 3, purified truncated LH U493C; lane 4, purified SH and truncated LH U493C – expression from the operon

For the enzymatic activity testing, the following gene layouts were chosen: *(i*) two-gene operons with the truncated *lh* gene variants (U493C, U493M, U493STOP), paired with the *sh dmb* gene and (*ii*) single subunit coding genes expressed in various compatible vectors. Cultures of *E. coli* harbouring the selected *dmb* genes were grown under intense aeration. From the tested variants, designs prepared in the pMCSG53 vector were selected as this vector provides a high yield of the POI with a cleavable His_6_-tag permitting fast protein purification. In case of the investigated two-gene operons (*sh* and *lh* genes in pMCSG53 vector), the biosynthesized SH subunit contained a cleavable N-terminal His_6_-tag. Total protein samples were taken after overnight cultivations and analysed using SDS-PAGE. SH and LH U493M and U493C subunits, expressed from the single-gene layouts, are visible as dominant protein bands (Fig. 2A, **lanes 1–6**), similarly to U493STOP variant (Supplementary Material File 3, Sect. 1.1, Suppl. Tables 1 and Suppl. Figure 1). In the case of two-gene operons, the SH-corresponding band is more prominent than the band representing the subsequently translated LH subunit (Fig. 2A, **lanes 7–8**), which was observed for both U493C and U493M variants. Nevertheless, both subunits expressed together were in their soluble form (Fig. 2B, **lanes 2 and 4**). This eliminated an extensive and time consuming solubilization of SH.

Because H2ases are easily inactivated by oxygen species, as well as other factors, it is crucial to develop fast and simple purification procedures. A following protocol was developed: (*i*) sonication of the bacterial cells, (*ii*) vacuum aided IMAC purification, (*iii*) buffer exchange. IMAC purification was conducted in two stages, with a final 500 mM imidazole concentration in the elution buffer. Step (*iii*) is crucial for removing contaminants as well as imidazole from the sample buffer before a spectrophotometric activity assays.

### H2ase activity measurements

Hydrogen evolution can be measured directly, for example by drawing a sample from the reaction mixture headspace ([42, 43], or indirectly by spectrophotometric measurement of change in absorbance. The indirect spectrophotometric methods recruit small-molecule dyes (oxidized or reduced by H2ases), such as benzyl viologen and methyl viologen [35, 36, 44–51]. For the spectrophotometric H2ase screening in multi-well plates, usually stoichiometric amounts of dithionite are used, or the H2 oxidation coupled to MV reduction in the absence of dithionite is tested. In this study, however, spectrophotometric measurements in the presence of excess dithionite were undertaken. The final preparations containing dimeric Dmb enzyme, obtained in vivo (via expression of the two-gene operon) or by in vitro assembly of the separately purified subunits, were immediately purged with argon gas and assayed with an indirect hydrogen evolution assay using methyl viologen. A sample of *E. coli* BL21-Gold(DE3) lysate was used as a negative control. The enzymatic activity of single LH and SH subunits was also investigated as a form of negative control. The average activity towards H_2_ evolution of the investigated samples is shown in Table 1. The results of the performed experiment indicate that the recombinant [NiFe] Dmb H2ase is capable of H2 evolution. It should be noted, however, that in the presence of excess dithionite, MV cannot be easily oxidised by an active H2ase, potentially leading to false negative results.

**Table 1:** Comparison of the recombinant Dmb hydrogenase variant activities in the spectrophotometric methyl viologen assay

Construct (sample tested)	average hydrogenase activity [µmoles H_2_/min/mg of protein]	standard deviation
LH U493C_STOP499_pMCSG53 + SH_pMCSG53 (in vitro assembly)	81.368	± 0.15
SH_rbs517_LH U493C_STOP499_pMCSG53 (in vivo assembly)	85.344	± 0.11
LH U493M_STOP499_pMCSG53 + SH_pMCSG53 (in vitro assembly)	28.644	± 0.11
SH_rbs517_LH U493M_STOP499_pMCSG53 (in vivo assembly)	38.278	± 0.01
LH U493M_STOP499_pMCSG53	0	0
LH U493C_STOP499_pMCSG53	0	0
SH_pMCSG53	0	0
assay buffer	0	0
*E. coli* BL21-Gold(DE3) strain (aerobic conditions)	0.0016	0
*E. coli* BL21-Gold(DE3) strain	0.0017	0

The recombinant Dmb H2ase preparations, containing 0.3 mg of the purified protein variant, were obtained via in vivo or in vitro assembly. The small H2ase subunit (SH) was subjected to a solubilization procedure prior to in vitro assembly. Single H2ase subunits: SH, LH U493C_STOP499 (truncated), LH U493M_STOP499 (truncated), prepared in a similar manner to the operon derived enzyme, showed no activity. A crude lysate from the *E. coli* BL21(DE3)Gold strain culture, which was grown in parallel with the IPTG induced recombinant Dmb H2ase producing cultures, was used as a negative control. An additional control sample of the strain, grown overnight in standard aerobic conditions, was also included. A sample of the assay buffer contained all of the components of the assay except the enzyme preparation/lysate. All the samples were prepared in triplicate and measurements were undertaken with shaking during 6 min kinetic runs with a measurement interval of 1 min.

The activity calculated for each measurement is shown in Table 1. The most active construct is the SH_LH U493C_pMCSG53 variant (expressed from a two-gene operon). Its’ nucleotide and amino acid sequence, as well as map of the recombinant construct, are shown in Supplementary Material File 5. Surprisingly, constructs comprising of LH U493M and SH also exhibited significant activity, about half of that of the recombinant [NiFe] Dmb H2ase variant. In case of in vitro assembled subunits, expressed and biosynthesized separately and mixed prior to the assay, the calculated H_2_ evolution activity is only slightly lower than for the operon derived Dmb enzyme (Table 1). The variant remains active even though SH purification was aided by urea solubilization, presumably affecting 4[Fe-S] cluster structures. No significant enzymatic activity was observed for the crude *E. coli* BL21(DE3)Gold lysates and single SH subunit. Interestingly, Caserta et al. recently demonstrated that the large subunit HoxC of the O_2_-sensitive *Ralstonia eutropha* [NiFe] H2ase was capable of H_2_ activation, although its activity was significantly lower compared to the native holoenzyme [52]. In contrast to the Caserta et al. results, no enzymatic activity of single LH Dmb subunit was detected using spectrophotometric methyl viologen assay.

**Fig. 3:**
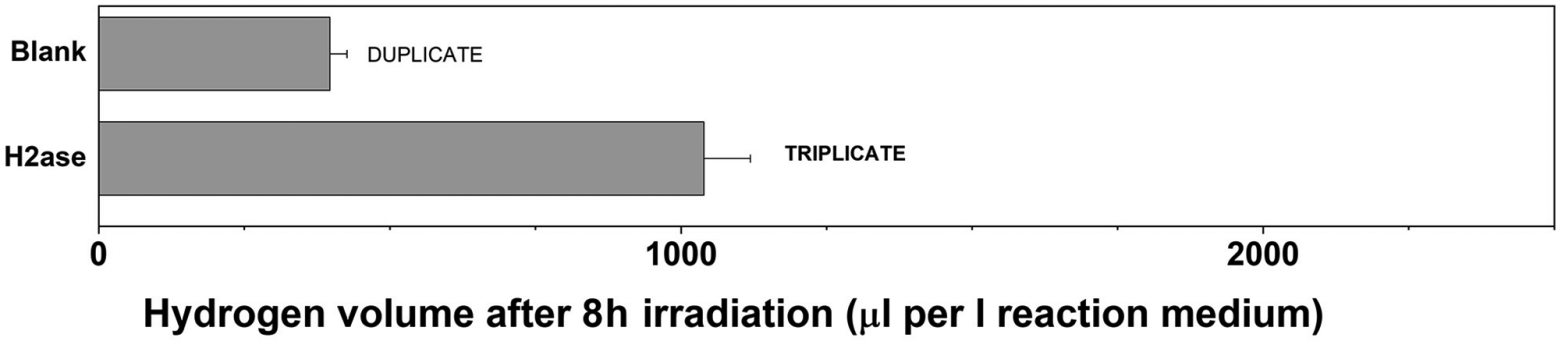
Photocatalytic hydrogen evolution assay. The deaerated mixture containing methyl viologen, L-cysteine and SH_LH U493C_pMCSG53 hydrogenase variant was irradiated using a 1000 W Xenon lamp at 10^o^C. After 8 h of irradiation, 200 ml of gas samples were collected from the headspace of the photoreactor and injected to the gas chromatograph, coupled with thermal conductivity detector (TCD). The amount of hydrogen was calculated at µl per litre of the reaction mixture

To finally confirm the recombinant Dmb H2ase activity, photocatalytic evolution assay was performed. For that purpose, the most promising Dmb variant SH_LH U493C_pMCSG53 (selected using the spectrophotometric assay) was chosen (Table 1; Supplementary Material File 5). The performed experiment clearly indicated that the recombinant Dmb H2ase is capable of H_2_ evolution (Fig. 3).

## Discussion

A large number of H2ases, belonging to different classes, were cloned and isolated from native and closely related hosts [8]. However, heterologous expression of these enzymes often results in an inactive enzyme. Most active hydrogenases are isolated from organisms with specific demands for cultivation: anaerobes and extremophiles. Generally, we lack the molecular tools to easily manipulate those microbes, but also cultivation of these strains often demands very specialized equipment and complex strategies. Obtaining a high yield of POI from such cultures requires enormous effort. At the same time, the exact mechanism and features enhancing the abilities of H2ases are still poorly understood. In order to produce a considerable amount of enzyme for characterization, testing and application in photoinduced water splitting experiments, an efficient and easy to handle expression system is needed. To generate expression construct producing an active enzyme, dozens of layouts were tested.

In summary, *D. baculatum* periplasmic [NiFeSe] hydrogenase and hydrogenase maturation protease genes (3 genes) were optimized for heterologous expression in *E. coli*. 4 different expression vectors were employed and more than 100 constructs created (Supplementary Material File 3). Site directed mutagenesis was performed to obtain all the designed LH variants. The resulting constructs were arranged in two-gene or three-gene synthetic operons (37 different layouts) (Supplementary Material File 3, Sect. 1.2 and 1.3). The need for specific protease (maturase) during Dmb hydrogenase maturation was dismissed by introduction of artificial truncation of the LH gene sequence, by means of mutagenesis. Out of the tested variants and designs, two operons: SH_LH U493M_pMCSG53 and SH_LH U493C_pMCSG53 resulted in a soluble H2ase with activity towards H_2_ evolution. These constructs differ from each other with the amino acid residue placed in position 493 of the polypeptide. In the native enzyme, this position is occupied by selenocysteine, encoded by the TGA codon. During translation, a specific structure (SECIS), following the TGA codon, determines an insertion of suitable amino acid instead of termination of the process. The *E. coli* SECIS structure differs from the one from *D. baculatum*, thus the incorporation of SeCys is not possible without interference in the H2ase structure. Attempts to create the SECIS element in the U493STOP mutant failed due to the high toxicity of the construct and immediate cell lysis after induction of the recombinant operon expression. Therefore, we constructed the U493C LH variant, which resulted in production of a partially active [NiFe] instead of [NiFeSe] hydrogenase. Unexpectedly, the other variant U493M of Dmb H2ase, which will be used in future for the planned experiments with selenomethionine insertion (this can be achieved simply by addition of the selenomethionine to the culture media), also exhibited some H2ase activity. This phenomenon should be further investigated, as no simple explanation for the role of methionine in the catalytic centre can be readily provided. Our results (concerning the U493C LH variant) corroborate with the results presented by Marques et al. [14], who constructed, characterized, and crystalized the U489C variant of the DvH NiFeSe H2ase, converting [NiFeSe] H2ase to a partially active [NiFe] enzyme. Interestingly, the U489C variant appeared to be Ni-depleted, revealing crucial involvement of SecCys residue in the enzyme maturation. In addition, Marques et al. showed that the active site of the purified U489C variant could be partially reconstituted by incubation with NiCl_2_ under H_2_.

The biosynthesis of SH turned out to be another drawback in this study. Typically for solubilization of this protein in vitro, a 2 M urea wash was used, followed by Fe-S cluster incorporation. However, the problem with low SH solubility was solved by co-expression of the SH encoding gene from a single operon with the truncated version of LH gene (Supplementary Material File 3).

While several vectors were used in this study, providing the POIs with or without His_6_-tag on either termini, the recombinant gene expression level, obtained for vector pMCSG53, seemed to exceed the others. Additionally, the resulting N-terminal His_6_-tag extension was crucial to reduce the time of the isolation procedure. Immediately after the purification step, activity tests were performed. We have aimed for a minimal number of steps and maximum reduction of time between bacterial pellet collection and further experiments with the enzymes. During purification, anaerobic conditions were not strictly maintained, thus the assayed H2ases experienced short periods of O_2_ exposure. A fast and simple protocol of purification of the recombinant [NiFe] Dmb H2ase, applicable for the production of active enzyme, even after short O_2_ exposure, was developed. It was previously shown that [NiFeSe] H2ases are able to recover after oxygen inactivation. Although the U493C variant remained active despite short O_2_ exposure during the purification process, it is probably less stable after O_2_ inactivation than the native [NiFeSe] H2ase. During the assay, only a small difference in calculated activity was visible between the enzyme variant assembled in vitro and in vivo. On the one hand, this proves the efficacy of the solubilization procedure, on the other hand it might be the result of Fe-S cluster inactivation by the applied conditions. In the future, the influence of purification procedure on the iron content in the recombinant Dmb H2ase could be investigated with spectroscopic studies.

According to literature, the specific activity of the native [NiFeSe] Dmb H2ase was found to be 500–2000 µmol H_2_/min/mg, depending on the culture medium, enzyme preparation and the H_2_ evolution assay [11, 26, 53, 54]. Although the performed assays indicated that the recombinant [NiFe] Dmb H2ase is capable of H_2_ evolution, experiments using direct electrochemical methods or gas chromatography should be performed to evaluate the enzyme properties. It needs to be stated that the enzyme exhibits only a fraction of the native [NiFeSe] Dmb H2ase activity. Further studies need also to be carried out to improve the recombinant enzyme capabilities. Among them, the site-specific UAG-programmed Sec insertion is planned to reconstitute the active site of the [NiFeSe] enzyme, as it was done for the [NiFe] Hyd-1 H2ase [55]. Additionally, biosynthesis of the recombinant Dmb H2ase subunits in other *E. coli* strains, such as derivatives of K-12 (which do not have issues in terms of nickel transport) or co-production of the *D. baculatum* Hyp maturation proteins may be considered to increase the catalytic activity of the recombinant Dmb H2ase. Such a strategy has been recently employed by Fan et al. to produce catalytically active, recombinant [NiFe] hydrogenase from *Cupriavidus necator* in *E. coli* [8]. Our future studies will also concern investigation of the iron content in the active site pocket of the isolated U493C LH subunit. Although, we did not observed catalytic activity of this subunit, it is possible that incubation of the purified protein with NiCl_2_ under H_2_ [14] may lead to reconstitution of its active site and potential conversion to minimal H2ase, similar to the catalytically competent, large subunit HoxC of the O_2_-sensitive [NiFe]-hydrogenase, derived from *Ralstonia eutropha* [52].

## Conclusions

Catalytically active recombinant [NiFe] Dmb H2ase may be biosynthesized in *E. coli* by co-expression of the SH encoding gene and the truncated version of the LH gene, organized into a single operon. Substitution of selenocysteine to cysteine in LH subunit results in production of an active [NiFe] instead of [NiFeSe] H2ase. The proposed approach could be advantageous for other H2ases, enabling the establishment of the minimal pathway for enzyme maturation.

## Electronic supplementary material

Below is the link to the electronic supplementary material.

Additional file 1: Primer sequences, thermal cycling schedule and protein expression vectors.

Additional file 2: Aligned sequences of native and optimized synthetic genes.

Additional file 3: Detailed experimental results concerning cloning, expression and purification of the Dmb operon components. 1.1. Separate cloning of the Dmb operon components. 1.2. Production of protein complexes via two genes layouts. 1.3. Production of hydrogenase complex via co-expression of three-piece operon elements. 1.4. Production of hydrogenase complex via in vitro assembly. 1.5. Periplasmic transport signal modifications and large subunit truncation. 1.6. Production of hydrogenase complex via co-expression of two-piece operon elements. 1.7. Optimization of the small subunit solubility. 1.8. Hydrogenase assembly in optimized conditions. 1.9. Production of hydrogenase complex via expression of two-piece operon elements.

Additional file 4: Procedures for the solubilization of SH constructs and for in vitro Fe-S clusters insertion.

Additional file 5: Genetic map and DNA sequence of the Dmb hydrogenase SH_LH_U493C_pMCSG53 recombinant construct.

## Data Availability

The datasets used and/or analysed during the current study are available from the corresponding author on reasonable request.
