# Supplementary material for: Promising approaches for the assembly of the catalytically active, recombinant Desulfomicrobium baculatum hydrogenase with substitutions at the active site
Source: Microb Cell Fact. 2023 Jul 21;22:134. doi: 10.1186/s12934-023-02127-w (PMC10362691; doi:10.1186/s12934-023-02127-w)
Supplement: Supplementary file 1 — Additional file 1: Primer sequences, thermal cycling schedule and protein expression vectors. [file 12934_2023_2127_MOESM1_ESM.pdf]

Supplementary Material File 1.

A. Primer sequences and thermal cycling schedule.

Thermal cycling schedule for TD-PCR procedures applied in this study

cycle	denaturation	annealing	Polymerization
1 - 2	3 min at 95°C		
3 - 4	30 s at 95°C	30 s at 62°C	5 min at 70°C
5 - 6	30 s at 95°C	30 s at 61°C	5 min at 70°C
7 - 8	30 s at 95°C	30 s at 60°C	5 min at 70°C
9 - 10	30 s at 95°C	30 s at 59°C	5 min at 70°C
11 - 43	30 s at 95°C	30 s at 58°C	5 min at 70°C

no.	PURPOSE	5’- 3’ DNA SEQUENCE
1	signal sequence insertion	AGGAGGTTAGATATGAAGATCAAGACAGGTGCACGCAT
2	signal sequence insertion	AGGAGGTTAGATATGACCGAGGGTGCTAAGAAAGCAC
3	signal sequence insertion	AGGAGGTTAGATATGAGTCAGGCTGCAACACCCG
4	signal sequence insertion	AGGAGGTTAGATATGGTCAAAGAGCTTGGACAGGAAAAC
5	signal sequence insertion	CACCACCACCACCATCATTGAGATCCGGCTGCTAACAA
6	signal sequence insertion	TGGTGGTGGTGGTGCTCTGCAACGTAAAATGGCGACTTC
7	signal sequence insertion	TTGGAAGTAGAGGTTCTCCTCTGCAACGTAAAATGGCGACTTC
8	signal sequence insertion	TTGGAAGTAGAGGTTCTCATCGATGTTCACAACGTGTTCCTCAC
9	signal sequence insertion	TTGGAAGTAGAGGTTCTCAGACACTAATGCACGAATTTCGCGAC
10	signal sequence insertion	CATATCTAACCTCCTTACTTAAAGTTAAACAAAATTATTTCTAGAGGGGAAT

no.	PURPOSE	5'- 3' DNA SEQUENCE
11	signal sequence insertion	AGGAGGTTAGATATGAGTCAGGCTGCAACACCC
12	signal sequence insertion	GCGTGCAGCACGTGCACGGCACAGCCCAGACATGGGTCATACGAGCGAACCAAAC
13	signal sequence insertion	GCGTGCAGCACGTGCACGGCACAGCCCAGTCATGGGTCATACGAGCGAACCAAAC
14	signal sequence insertion	GTCAATGTTGACAACGTGTTCTTCACCGGTCTCAGCGTGCAGCACGTGCACGGCA
15	signal sequence insertion	TTGGAAGTAGAGGTTCTCGTCAATGTTGACAACGTGTTCTTCACC
16	operon	TTATCCACTTCCAATGTTACTCTGCAACGTAAAATGGCGACTTC
17	operon	TTATCCACTTCCAATGTTAATCGATGTTCACAACGTGTTCCTCAC
18	operon	TTATCCACTTCCAATGTTAGTCAATGTTGACAACGTGTTCTTCACC
19	operon	TTATCCACTTCCAATGTTAAGACACTAATGCACGAATTTCGCGAC
20	operon	TGGTGGTGGTGGTGATCGATGTTCACAACGTGTTCCTCAC
21	operon	TGGTGGTGGTGGTGGTCAATGTTGACAACGTGTTCTTCACC
22	operon	TGGTGGTGGTGGTGAGACACTAATGCACGAATTTCGCGAC
23	signal sequence modification and large subunit truncation	TACTTCCAATCCAATGCCAGTCAGGCTGCAACACCCGCT
24	signal sequence modification and large subunit truncation	TACTTCCAATCCAATGCCAGTCAGGCTGCAACACCCGCT
25	signal sequence modification and large subunit truncation	TACTTCCAATCCAATGCCACCGAGGGTGCTAAGAAAGCACCA
26	signal sequence modification and large subunit truncation	CACCACCACCACCATCATTGAGATCCGGCTGCTAACAA
27	signal sequence modification and large subunit truncation	CACCACCACCACCATCATTAAGGTAAAGAAACCGCTGCT
28	signal sequence modification and large subunit truncation	AACCTCTACTTCCAAGGTCATCATCACCATCACCATTAA
29	signal sequence modification and large subunit truncation	AACCTCTACTTCCAAGGTCATCATCACCATCACCATT
30	signal sequence modification and large subunit truncation	AGGAGGTTAGATATGAGTCAGGCTGCAACACCC
31	signal sequence modification and large subunit	GGAGGAAAAGAAATGAAGAAAATCTGGCTTGCACTGGCT

no.	PURPOSE	5'- 3' DNA SEQUENCE
	truncation	
32	signal sequence modification and large subunit truncation	GGAGGAAAAGAAATGACCGAGGGTGCTAAGAAAGCAC
33	signal sequence modification and large subunit truncation	GGAGGAAAAGAAATGGTCAAAGAGCTTGGACAGGAAAAC
34	signal sequence modification and large subunit truncation	TTCTCTGCTTCTGCTAGTCAGGCTGCAACACCCGCT
35	signal sequence modification and large subunit truncation	AGGAGGTTAGATATGAGTCAGGCTGCAACACCCG
36	signal sequence modification and large subunit truncation	TTATCCACTTCCAATGTTAGTCAATGTTGACAACGTGTTCTTCACC
37	signal sequence modification and large subunit truncation	CATTTCTTTTCCTCCTTAATCGATGTTCACAACGTGTTCCTCAC
38	signal sequence modification and large subunit truncation	CATTTCTTTTCCTCCCTATTAGTCAATGTTGACAACGTGTTCTTCACC
39	signal sequence modification and large subunit truncation	CATTTCTTTTCCTCCCTATTAATCGATGTTCACAACGTGTTCCTCAC
40	signal sequence modification and large subunit truncation	CATTTCTTTTCCTCCTTAGTCAATGTTGACAACGTGTTCTTCACC
41	signal sequence modification and large subunit truncation	CATTTCTTTTCCTCCTTACTCTGCAACGTAAAATGGCGACTTC
42	signal sequence modification and large subunit truncation	CATTTCTTTTCCTCCCTATTACTCTGCAACGTAAAATGGCGACTTC
43	signal sequence modification and large subunit truncation	TTGGAAGTAGAGGTTCTCGTCAATGTTGACAACGTGTTCTTCACC
44	signal sequence modification and large subunit truncation	TGGTGGTGGTGGTGTTAAGACACTAATGCACGAATTTCGCGAC
45	signal sequence modification and large subunit truncation	TGGTGGTGGTGGTGGTCAATGTTGACAACGTGTTCTTCACC
46	signal sequence modification and large subunit	GCGTGCAGAACATGAACGGCGCAACCCAATCATGGGTCATACGAGCGAACCAAAC

no.	PURPOSE	5'- 3' DNA SEQUENCE
	truncation	
47	signal sequence modification and large subunit truncation	GCGTGCAGAACTGCAACGGCGCAACCCAATCATGGGTCATACGAGCGAACCAAAC
48	operon	CACCACCACCACCATCATCATTAAGATCCGGCTGCTAACAAAGC
49	operon	GCTTCTGCTCTCGCTAAGATCGTCAAAGAGCTTGGACAGGAAAACGA
50	operon	TTCTCTGCTTCTGCTGCTAGTCAGGCTGCAACACCCGCT
51	operon	TTCTCTGCTTCTGCTGCTGTCAAAGAGCTTGGACAGGAAAACGA
52	operon	GCTTCTGCTCTCGCTAAGATCAGTCAGGCTGCAACACCCGCT
53	operon	CACCACCACCACCATCATTAACTGGTAAAGAAACCGCTGCTGCGAA
54	operon	TACTTCCAATCCAATGCCGCGGAGGTGACTCCGGAG
55	operon	TTTAAGAAGGAGATATACATATGAAGAAAATCTGGCTTGCACTGGCTGGTT
56	operon	AGGAGGTTAGATATGGCGGAGGTGACTCCGGAG
57	operon	AGGAGGTTAGATATGTCCGCCTCCGATGCCGTC
58	operon	AGGAGGTTAGATATGGACGATGCGATCGGGATTAATCC
59	operon	AGGAGGTTAGATATGATCGCACAGCAATGGGCGATTTTTC
60	operon	TTCTCTGCTTCTGCTGCTACCGAGGGTGCTAAGAAAGCACCA
61	operon	GCTTCTGCTCTCGCTAAGATCACCGAGGGTGCTAAGAAAGCACCA
62	operon	AGCGAGAGCAGAAGCTGAGAACATCATCGTCGTT
63	operon	TGGTGGTGGTGGTGTTAATCGATGTTCACAACGTGTTCCTCAC
64	operon	TGGTGGTGGTGGTGTTAATGGACTGCGCAACCCAACATTGG
65	operon	TGGTGGTGGTGGTGTTAGTCAATGTTGACAACGTGTTCTTCACC
66	operon	TGGTGGTGGTGGTGTTAGTGCACGGCACAGCCCAGACAT
67	operon	TATATCTCCTTCTTAAAGTTAAACAAAATTATTTCTACAGGGGAATTGT
68	operon	TTATCCACTTCCAATGTTAATGGACTGCGCAACCCAACATTGG
69	operon	TTATCCACTTCCAATGTTAGTGCACGGCACAGCCCAGACAT
70	operon	TTATCCACTTCCAATGTTATAGATAAGGTTTCAAAATGGCGTCTTTCAC
71	operon	TTGGAAGTAGAGGTTCTCTAGATAAGGTTTCAAAATGGCGTCTTTCAC
72	operon	TTGGAAGTAGAGGTTCTCTTAATGGACTGCGCAACCCAACATTGG

no.	PURPOSE	5'- 3' DNA SEQUENCE
73	operon	TTGGAAGTAGAGGTTCTCCCAATACAGGGCCGCCATGC
74	operon	TTGGAAGTAGAGGTTCTCTTAGTGCACGGCACAGCCCAGACAT
75	operon	TTGGAAGTAGAGGTTCTCCATACCATCACCCTGATTTCTAGCAATATAT
76	operon	TTGGAAGTAGAGGTTCTCCTGATTTCTAGCAATATATGCCAACATAGTCAAA
77	operon	AGCAGCAGAAGCAGAGAATGCTAACACCAAACCA
78	operon	GATCTTAGCGAGAGCAGAAGCTGAGAACATCATCGTCGTT
79	operon	TTCTCTGCTTCTGCTGCTAGTCAGGCTGCAACACCCGCT
80	operon	GGAGGAAAAGAAATGAGTCAGGCTGCAACACCC
81	operon	GGAGGAAAAGAAATGAGTCAGGCTGCAACACCC
82	operon	GGAGGAAAAGAAATGAGTCAGGCTGCAACACCCG
83	operon	TGGTGGTGGTGGTGTTAATCGATGTTCACAACGTGTTCCTCACCT
84	operon	GTGGTGGTGGTGTTAGTGCACGGCACAGCCCAGTCAT

B. Expression vectors* used in this study.

*All plasmid backbones were kindly provided by Robert Jędrzejczak from the Structural Biology Centre at the Argonne National Laboratory.

pMCSG53 expression vector

pMCSG53 is an expression vector harboring two genes coding for rare tRNAs-with AGG/AGA coding for arginine and AUA coding for isoleucine. It has a high-copy-number ColE1/pMB1/pBR322/pUC origin of replication, compatible with pRSF1-MCSG for co-expression. The gene of interest is cloned into the LIC site under the T7 promoter. The POI is biosynthesized with a His-tag on the N-terminus of the resulting protein (Eschenfeldt et al., 2013).

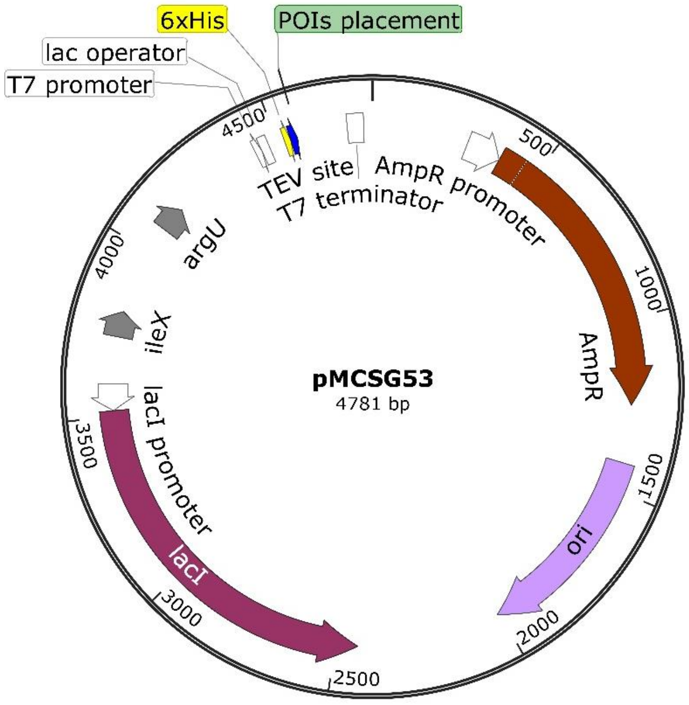

Schematic representation of vector pMCSG53 properties. ileX- region coding for rare isoleucine tRNA, argU – region coding for rare arginine tRNAs, 6xHis – region coding for six histidine tag, AmpR – region confers resistance to the antibiotic ampicillin, ori – high-copy-number ColE1/pMB1/pBR322/pUC origin of replication, lacI – lac repressor. Map created with SnapGene software (www.snapgene.com).

atg cac cat cat cat cat cat gag aac ctg tac ttc caa tcc aat gcc **XXX** taa

M H H H H H H E N L Y F Q S N A **X** *

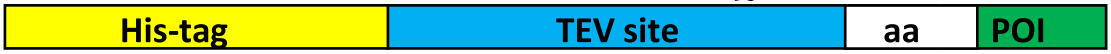

POI expressed from vector pMCSG53. The placement of the POI is marked as **X**. The STOP codon (taa) is indicated by a red asterisk. Amino acids inserted additionally after the TEV recognition sequence are marked as **aa**.

pRSF1-MCSG expression vector

pRSF1-MCSG is an expression vector. The RSF 1030 origin of replication enables the implementation of a two-plasmid system. It is compatible with the pMCSG53 origin of replication. The gene of interest is cloned into the LIC site under the T7 promoter. The POI is biosynthesized without a His-tag.

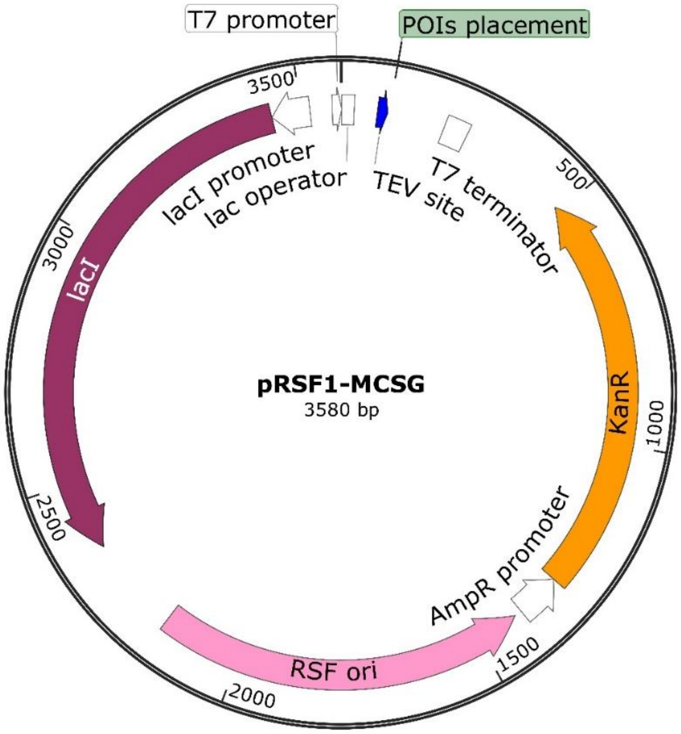

Schematic representation of vector pRSF1-MCSG properties. KanR – region confers resistance to the antibiotic kanamycin, RSF ori – allows for propagation in *E. coli* cells that contain additional plasmids with compatible origins, lacI – lac repressor. Map created with SnapGene software (www.snapgene.com).

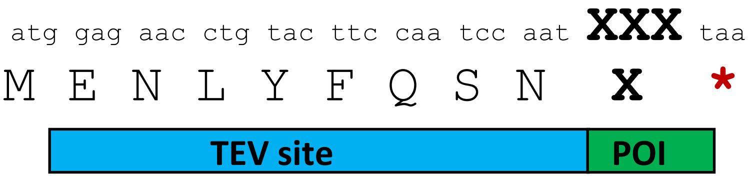

POI expressed from vector pRSF. The POI placement is marked as **X**. The STOP codon (taa) is indicated by a red asterisk.

pMCSG92 expression vector

pMCSG92 is an expression vector, like pMCSG53, harboring two genes coding for rare tRNAs - AGG/AGA for arginine and AUA for isoleucine. It has also the same origin of replication and antibiotic resistance. The gene of interest is cloned in the LIC site under the T7 promoter. The POI is biosynthesized with a His-tag on the C-terminus of the resulting protein, with a preceding TEV cleavage site.

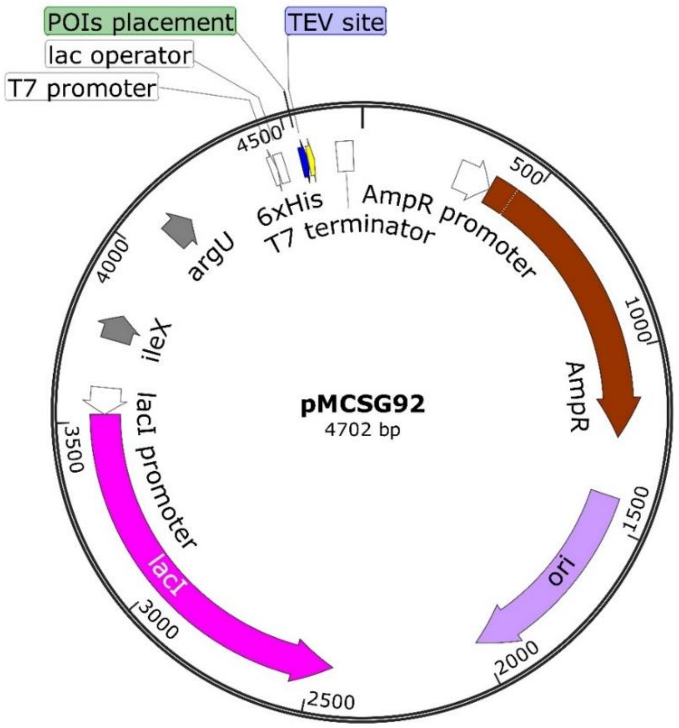

Schematic representation of vector pMCSG92 properties. ileX- region coding for rare isoleucine tRNA, argU – region coding for rare arginine tRNAs, AmpR – region confers resistance to the antibiotic ampicillin, ori – high-copy-number ColE1/pMB1/pBR322/pUC origin of replication, region coding for six histidine tag is marked as a yellow arrow. Map created with SnapGene software (www.snapgene.com).

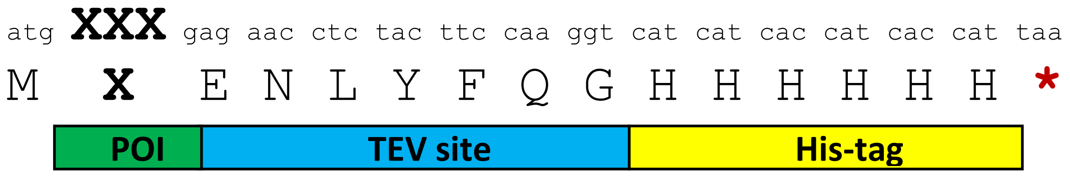

POI expressed from vector pMCSG92. POIs placement is marked as X. The STOP codon (taa) is indicated by a red asterisk.

pMCSG93expression vector

pMCSG93 is based on pRSF1-MCSG and shares most of its functional features, with one exception: the TEV cleavage site preceding the His-tag is introduced at the C-terminus of the resulting POI.

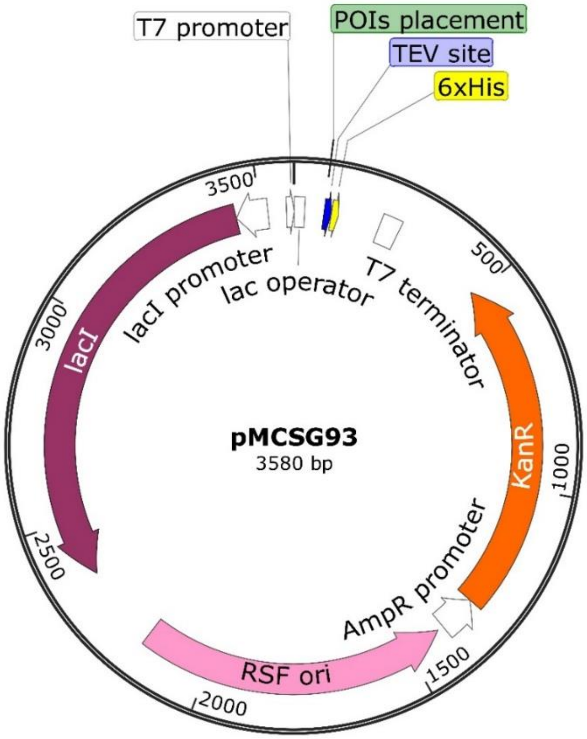

Schematic representation of vector pMCSG93 properties. KanR – region confers resistance to the antibiotic kanamycin, RSF ori – allows for propagation in *E. coli* cells that contain additional plasmids with compatible origins, lacI – lac repressor. Map created with SnapGene software (www.snapgene.com).

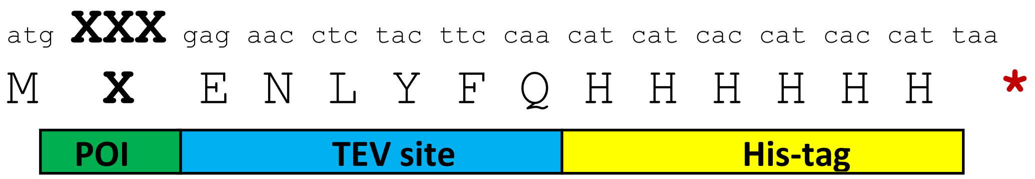

POI expressed from vector 93. POI placement is marked as X. The STOP codon (taa) is indicated by a red asterisk. Amino acids inserted additionally after the TEV recognition sequence are marked as **aa**.

References

Eschenfeldt, W. H., Makowska-Grzyska, M., Stols, L., Donnelly, M. I., Jedrzejczak, R., & Joachimiak, A. (2013). New LIC vectors for production of proteins from genes containing rare codons. Journal of Structural and Functional Genomics, 14(4), 135–144. https://doi.org/10.1007/s10969-013-9163-9
