# Supplementary material for: Promising approaches for the assembly of the catalytically active, recombinant Desulfomicrobium baculatum hydrogenase with substitutions at the active site
Source: Microb Cell Fact. 2023 Jul 21;22:134. doi: 10.1186/s12934-023-02127-w (PMC10362691; doi:10.1186/s12934-023-02127-w)
Supplement: Supplementary file 2 — Additional file 2: Aligned sequences of native and optimized synthetic genes. [file 12934_2023_2127_MOESM2_ESM.pdf]

Supplementary Material File 2.

Aligned sequences of native and optimized synthetic genes.

A. Aligned sequences of the genes coding for the large subunit: synthetic, optimized (LH_M) and native (LH_wt). Nucleotides of the native sequence that are the same as in the synthetic sequence are marked with dots. Nucleotides that were changed due to optimization are marked in red. A green frame indicates alternation leading to a change in translation (STOP493M).

```
LH_M    1   ATGAGTCAGGCTGCAACACCCGCTGCCGACGGTAAAGTTAAGATCTCGATCGATCCATTG  60
LH_wt   1   G..TCA..A..A..T..T.....A..T.....G...........T..C..T.....G...  60

LH_M    61  ACTCGTGTCGAGGGTCACCTTAAGATCGAAGTAGAGGTAAAGGATGGAAAGGTGGTAGAC  120
LH_wt   61  ..C..G..T..A.....T..C.....T.....T..A..C..A..C..C.....C..C..T  120

LH_M    121 GCTAAGTGCAGTGGTGGTATGTTTCGTGGCTTCGAGCAGATCTTGCGTGGTCGTGACCCT  180
LH_wt   121 ..C.....TTCC..C..A.....C.....G...........TC....C..C..C..T..C  180

LH_M    181 CGTGATTCGTCACAGATTGTTCAGCGCATCTGTGGCGTTTGTCCAACAGCGCACTGCACG  240
LH_wt   181 A.G.....T..C.....C..A...........C.....G..C..C..G........T...  240

LH_M    241 GCATCCGTCATGGCGCAAGATGACGCATTTGGAGTGAAAGTCACAACGAACGGGCGCATT  300
LH_wt   241 ..T...........C..G........C..C..C..C.....A..C..C..T..C......  300

LH_M    301 ACGCGTAACCTGATCTTCGGAGCTAACTACTTGCAGAGTCACATCCTGCACTTCTACCAT  360
LH_wt   301 ..C.................C..C.....TC.....TC...T..T.....T..T..T..C  360

LH_M    361 CTTGCAGCTCTGGATTATGTCAAAGGTCCTGACGTGTCTCCATTCGTACCACGCTATGCC  420
LH_wt   361 T.G..C..C..............G..C..C..T..A..C..C..T..T..C..T..C...  420

LH_M    421 AACGCGGACCTTTTGACTGACCGTATCAAGGATGGCGCAAAGGCTGATGCCACCAACACG  480
LH_wt   421 ..T.....T..G.....G..T..C........C..A..C.....C.....A........C  480

LH_M    481 TATGGACTGAATCAGTACTTGAAGGCCTTGGAGATCCGTCGCATTTGCCACGAAATGGTC  540
LH_wt   481 ..C..CT...........C.......AC....A.....C.....C..T..T..G......  540

LH_M    541 GCTATGTTTGGTGGTCGTATGCCACATGTACAAGGTATGGTTGTAGGAGGTGCTACAGAA  600
LH_wt   541 ..C.....C..C.....C.....T.....T..G..C.....C..G..C.....A..C..G  600

LH_M    601 ATTCCCACAGCGGATAAGGTCGCAGAGTACGCAGCTCGCTTCAAAGAGGTCCAAAAGTTC  660
LH_wt   601 ..............C.....T..C..A........C........G..A.....G..A...  660

LH_M    661 GTCATCGAGGAATACTTGCCTTTGATCTACACACTTGGATCGGTTTACACTGACCTTTTC  720
LH_wt   661 ..G...........TC.....C..........T..G..T..C..G.....C..T..G...  720

LH_M    721 GAGACCGGTATTGGATGGAAGAACGTCATCGCTTTCGGTGTGTTCCCAGAGGACGACGAT  780
LH_wt   721 ........C..C..C..........................T.....C..A.........  780
```

```
LH_M    781 TACAAGACTTTCTTGTTGAAACCGGGTGTCTACATCGACGGTAAGGACGAGGAGTTCGAC  840
LH_wt   781 ........C...C..C.C.....C.................C...........A.....T  840

LH_M    841 AGCAAACTTGTTAAGGAATACGTTGGTCACTCCTTTTTCGATCATAGTGCCCCAGGAGGG  900
LH_wt   841 TC...G..G..C........T..C..A..T.....C..T..C...TCC..T..C..C..C  900

LH_M    901 CTGCACTATAGCGTCGGGGAGACAAACCCTAATCCTGACAAACCTGGCGCCTATTCATTT  960
LH_wt   901 ........C........C.....G..T..C.....G.....G..C.....A..CAGC..C  960

LH_M    961 GTAAAAGCACCCCGTTACAAGGATAAACCGTGCGAGGTAGGGCCTTTAGCTCGTATGTGG  1020
LH_wt   961 ..C..G..T..............C..G..C.....A..C..T..GC.G..C..C......  1020

LH_M   1021 GTCCAAAACCCGGAGTTGTCACCTGTGGGGCAGAAACTGTTAAAAGAACTTTATGGAATT  1080
LH_wt  1021 .....G........AC..AGC..C..T..C.....G...C.C..G.....C..C..C...  1080

LH_M   1081 GAAGCCAAGAACTTTCGCGATCTTGGCGACAAAGCATTCTCTATCATGGGCCGTCATGTA  1140
LH_wt  1081 ..............C........G........G..T.....C...........C..C..G  1140

LH_M   1141 GCACGTGCTGAAGAGACCTGGTTGACTGCGGTCGCAGTGGAGAAGTGGTTAAAGCAGGTC  1200
LH_wt  1141 ..T.....G............C.T..C..C..T..C..T..A..A...C.C........T  1200

LH_M   1201 CAGCCTGGTGCGGAGACTTATGTCAAGAGCGAAATTCCGGACGCAGCAGAGGGTACCGGT  1260
LH_wt  1201 .....C..C..C..A..C..C......TC...G........T..C..C..A..C......  1260

LH_M   1261 TTCACAGAAGCGCCTCGTGGAGCGCTGCTGCACTACTTGAAGATCAAGGATAAAAAGATC  1320
LH_wt  1261 .....G.....T..C..C..C..AT..........TC..........A..C..G......  1320

LH_M   1321 GAGAACTACCAAATTGTCTCAGCCACACTGTGGAATGCTAACCCTCGCGATGATATGGGC  1380
LH_wt  1321 .....T..T..G..C..G..T..G..T..T.....C..C.....CA.A.....C......  1380

LH_M   1381 CAGCGTGGACCAATTGAGGAGGCACTTATCGGTGTCCCAGTGCCTGATATCAAGAACCCA  1440
LH_wt  1381 .....C..T..G..C.....A..C..C.....C..G..G..T..C..C........T..C  1440

LH_M   1441 GTGAACGTGGGTCGTTTGGTTCGCTCGTATGACCCAATGTTGGGTTGCGCAGTCCATGTG  1500
LH_wt  1441 ..C..T.....G..CC..........C..C.....GTGAC....C..T..C..G..C...  1500

LH_M   1501 CTGCACGCTGAAACAGGTGAGGAACACGTTGTGAACATCGA  1541
LH_wt  1501 ...........G..C.....A...........C.....T..  1541
```

B. Aligned sequences of the genes coding for the small subunit: synthetic, optimized (SH_opt) and native (SH_wt). Native sequence nucleotides that are the same as in the synthetic sequence are marked with dots. Nucleotides that were changed due to optimization are marked in red. The native SH_wt sequence starts with a signal sequence (81 bp), thus the START codon is not included.

```
SH_opt 1 ATGACCGAGGGTGCTAAGAAAGCACCAGTGATTTGGGTGCAGGGTCAGGGTTGCACTGGT   60
SH_wt  1 ---.....A..A..C..A.....T..G..C..C.....A.....A........T......   60

SH_opt 61 TGTTCCGTTTCACTGCTGAATGCGGTACATCCGCGTATTAAAGAAATCCTGCTGGACGTG  120
SH_wt  61 ..C........T.....C..C..A..C.....CA.A..C..G..G..T........T...  120

SH_opt 121 ATTTCACTGGAATTTCATCCGACTGTTATGGCTAGTGAAGGAGAAATGGCACTGGCGCAC 180
SH_wt  121 ..CAGC..T..G..C.....C..C..C.....A........T..G.....T........T 180

SH_opt 181 ATGTATGAAATTGCGGAAAAGTTCAACGGCAACTTCTTTTTGTTAGTGGAGGGCGCCATC 240
SH_wt 181  .....C........T........T...........T..C...C.G.....A..T...... 240

SH_opt 241 CCAACTGCTAAGGAAGGACGCTACTGTGTTGTAGGTGAGACACTGGACGCGAAGGGTCAC 300
SH_wt  241 ..C..C..C........T........C.....C.....A..T.....T..C..A..G..T 300

SH_opt 301 CACCATGAAATTACGATGATGGAGTTAATTCGCGACCTTGCCCCCAAGAGTCTTGCAACT 360
SH_wt  301 ..T........C..C........AC.G..C..G..T..G..A......TC...G..C..C 360

SH_opt 361 GTAGCTATTGGTACCTGCGCAGCTTATGGTGGCATCCCAGCTGCAGCTGGAAATGTTACG 420
SH_wt  361 ..G..C..A.....T..T..C.....C..C.....T..C..G..T..A..C..C..C..C 420

SH_opt 421 GGCTCTAAGTCAGTGCGTGATTTCTTTGCCGAAGAAAAAATCGAGAAGCTGCTGGTAAAC 480
SH_wt  421 .....C...AGC........C..............G..G.....A..A........C... 480

SH_opt 481 GTGCCAGGTTGTCCTCCTCACCCAGATTGGATGGTAGGTACGTTAGTTGCGGCATGGTCT 540
SH_wt  481 .....C..A.....G..C..T..G..C........C..C..TC.G.....C......AGC 540

SH_opt 541 CACGTTCTGAACCCGACTGAACATCCTTTACCCGAGTTGGATGATGATGGCCGTCCGTTG 600
SH_wt  541 ..T..C..C..T.....C..G.....CC.G.....A...........C.....C...C.. 600

SH_opt 601 CTTTTCTTCGGCGATAACATCCATGAGAACTGTCCTTACCTGGACAAGTACGATAACAGT 660
SH_wt  601 ..G.....T.....C........C...........G..T..T..T..A.....C...TCC 660

SH_opt 661 GAATTTGCAGAGACCTTTACCAAACCCGGTTGCAAAGCAGAATTGGGATGCAAAGGACCC 720
SH_wt  661 .....C..G..A.....C.....G..G..C.....G..C...C.T..C.....G..T..G 720

SH_opt 721 TCCACGTACGCGGACTGCGCTAAGCGTCGTTGGAATAACGGTATCAATTGGTGTGTAGAA 780
SH_wt  721 .....C..T..C..T.....C........C.....C.....C..A..C........C..G 780

SH_opt 781 AACGCAGTGTGCATTGGTTGTGTCGAGCCCGACTTCCCAGACGGGAAGTCGCCATTTTAC 840
SH_wt  781 .....C.....T..C..C.....G..A..G.....T..G.....A.....T..T..C..T 840

SH_opt 841  GTTGCAGA   848
SH_wt  841  ..A..G..   848
```

C. Aligned sequences of the genes coding for the maturation protease: synthetic, optimized (HMP_opt) and native (HMP_wt). Nucleotides of the native sequence that are the same as in the synthetic sequence are marked with dots. Nucleotides that were changed due to optimization are marked in red. The native sequence starts with a signal sequence (69 bp), thus the START codon is not included.

```
HMP_opt 1   ATGGTCAAAGAGCTTGGACAGGAAAACGAGTGGGATCCCGATAAAGTGGACTTCTTGGAC  60
HMP_wt  1   ---.....G..A..C..C........T...........G..C..G..C..T..TC.....  60

HMP_opt 61  GGAGCTACGTTCACCCAGGATATCTTCTACATCTTCCAGGAGTATGAACGTGTGTTGGTT  120
HMP_wt  61  .....C..C..T..T........T..T..T..T..T.....A..C.....C..TC.T..G  120

HMP_opt 121 CTGGACACGGTAAAGGGTGGTCGTGAGCCAGGTACTGTATATCGTTTCACGGAGGAGAAT  180
HMP_wt  121 ..C.....C..C.....C..C..G..A..C..A..G..G........T...........C  180

HMP_opt 181 TTGCGTGACAACTATCAACAGCGCCTGTCTTTACACGATATTGACCTGTTAGATTCGTTG  240
HMP_wt  181 C....G.....T.....G...........GC.G..T..C..C..T...C.G......C..  240

HMP_opt 241 AAGATGGCTGAGTTACTGGGGAATAAGCCTGAACTGATGGTAATCGGCATCGAACCACTT  300
HMP_wt  241 ..A.....G.....G.....A..C..A..C.....C.....C..............CT.G  300

HMP_opt 301 ACTATCTCCGAGTGGTCAATGGAGCTTTCTGCACCAGTTAAGGATAAGTACCCGAAGTTC  360
HMP_wt  301 ..C...AG.........C......T.G..G..T..C..C...........T.....A..T  360

HMP_opt 361 TTGGAAGCTGCTCGTCGCGAAATTCGTGCATTAGTGTC  398
HMP_wt  361 C....G..A..G..C........C..C..GC.C.....  398
```
