# Supplementary material for: Promising approaches for the assembly of the catalytically active, recombinant Desulfomicrobium baculatum hydrogenase with substitutions at the active site
Source: Microb Cell Fact. 2023 Jul 21;22:134. doi: 10.1186/s12934-023-02127-w (PMC10362691; doi:10.1186/s12934-023-02127-w)
Supplement: Supplementary file 3 — Additional file 3: Detailed experimental results concerning cloning, expression and purification of the Dmb operon components. 1.1. Separate cloning of the Dmb operon components. 1.2. Production of protein complexes via two genes layouts. 1.3. Production of hydrogenase complex via co-expression of three-piece operon elements. 1.4. Production of hydrogenase complex via in vitro assembly. 1.5. Periplasmic transport signal modifications and large subunit truncation. 1.6. Production of hydrogenase complex via co-expression of two-piece operon elements. 1.7. Optimization of the small subunit solubility. 1.8. Hydrogenase assembly in optimized conditions. 1.9. Production of hydrogenase complex via expression of two-piece operon elements. [file 12934_2023_2127_MOESM3_ESM.pdf]

Supplementary file 3

1.1 Separate cloning of the *Dmb* operon components

The minimal operon of the NiFeSe *Dmb* H2ase consists of three genes that encode two hydrogenase subunits and a specific maturase. In the first stage of the project, the genes from the hydrogenase operon were cloned and expressed separately in various vector configurations. For this purpose, the designed inserts were amplified and the recombinant constructs for the expression of single proteins were prepared.

Due to the properties of the native hydrogenase genes, and differences between native and heterologous host, DNA fragments encoding signal sequences for periplasmic transport were incorporated into the designed constructs. Four signal sequences were tested: MalE and DsbA in both shorter and longer versions. These DNA fragments were incorporated by means of PCR either into the vector or into the insert sequences.

To prepare the designed synthetic genes with suitable overhangs and vectors enabling the fusion of the DsbA signal to the recombinant protein, PCR reactions were performed. The LIC cloning method requires the presence of single-stranded, complementary overhangs in the insert and vector sequence. These overhangs are created by T4 DNA polymerase from specifically designed DNA fragments, introduced into the sequence via PCR.

The PCR results were analyzed controlled on agarose gels. All planned reactions were conducted successfully with a single band of desired product observed.

1.1.1 PCR amplification with addition of signal sequences

Inserts for different vectors were prepared with LH mutants U493C, U493M, U493STOP, as well as SH and HMP as templates. The main goal of this PCR round was to create a range of primary inserts with terminal sequences complementary for 5 vectors used in this study. Vectors pRSF and pMCSG53 have identical flanking sequences for the subcloned genes, thus one version of the insert can be used for annealing with either of them. Vector

pMCSG53 carries ampicillin resistance and allows for overproduction of proteins with fused N-terminal His-tags. Vector pRSF carries resistance to ampicillin and allows for the overproduction of non-tagged proteins. The origins of replication of these vectors are compatible. Thus, a pair can be used for coexpression of genes from two plasmids in one bacterial cell. The same principle applies for the pMCSG92 and pMCSG93 vector pair. Vector 93 carries kanamycin resistance and the resulting recombinant protein has an additional His-tag preceded by a TEV site on the C-terminus. Vector 92 carries ampicillin resistance. The recombinant protein variants obtained with the use of v92 recombinant constructs have C-terminal His-tags preceded by a TEV site. Vector DsbA is based on the vector 93 backbone and requires the addition of the DsbA signal sequence encoding DNA fragment to the insert in order to be annealed.

1.1.2 Annealing and transformation

PCR reactions were cleaned-up from excess dNTPs and subjected to reaction with T4 DNA polymerase. The alignment of the insert/vector pairs, as well as insert modifications obtained by PCR reactions, are listed in Suppl. Tab.1. Annealing and transformation were performed according to the protocol described in the Methods section of the manuscript. The protocol for bacterial growth and the genes expression is provided in the main manuscript. Transformants were observed after 18 hours of incubation for all reactions (Suppl. Tab.1). A total of 36 types of recombinant *E. coli* variants were obtained.

1.1.3 Expression

Three colonies were selected from each transformation well and used to inoculate 1 ml of LB. Bacterial cultures were spun down after 18 hours from IPTG induction of the recombinant gene expression. Bacterial cells from each well were lysed, 4 µl samples were taken and analyzed by SDS-PAGE.

Constructs with positive results from the expression screening were chosen for plasmid DNA isolation and DNA sequencing. Based on the sequencing results, plasmid DNAs isolated from positive bacterial clones were used as templates for the next round of PCR, aiming for the construction of the designed operons.

Out of the obtained recombinant bacterial clones (with confirmed biosynthesis of POIs), twenty-nine recombinant constructs were purified and sequenced. All the constructs are listed in Suppl. Tab.1, along with gel numbers and lanes for reference (Suppl. Fig.1A and B).

Suppl. Tab.1. Properties of the three main target protein variants, produced in the first stage of the research and marked positive after DNA sequencing. Location of His-tag and TEV site are marked as N-term (located at N-terminus) or C-term (located at C-terminus). The POI biosynthesis level is evaluated using a three-point scale (1- low, 2- medium, 3- high). The biosynthesis of the obtained recombinant protein variants was investigated by SDS-PAGE. Gel and lane numbers corresponding to the investigated recombinant constructs are provided below in Suppl. Fig.1.

**target protein**	**mutagenesis**	**#**	**signal sequence**	**vector name**	**His-tag**	**TEV sequence**	**level of biosynthesis**	**gel no. Suppl. Fig.1AB**	**lane no.**
**HMP**	no	1	no	pMCSG53	N-term	N-term	3	I	7
		2	DsbA, N-term	vDsbA	C-term	No	1	III	14
		3	no	pMCSG92	C-term	C-term	3	III	15
		4	no	pRSF	no	N-term	1	III	20
**LH**	U493C (two-step mutagenesis)	5	no	pMCSG93	no	C-term	3	II	21
	U493C	6	no	pMCSG93	no	C-term	2	I	2
		7	DsbA, N-term	vDsbA	C-term	No	3	II	14
		8	no	pMCSG92	C-term	C-term	3	I	17
		9	no	pMCSG53	N-term	N-term	1	IV	5
		10	no	pMCSG92	C-term	C-term	2	IV	9
		11	no	pRSF	no	no	2	IV	21
	U493M	12	no	pMCSG92	C-term	C-term	2	II	4
		13	DsbA, N-term	vDsbA	C-term	no	2	II	7
		14	no	pMCSG53	N-term	N-term	3	III	12
		15	no	pRSF	no	no	3	IV	25
	U493C (two-step mutagenesis)	16	no	pMCSG92	C-term	C-term	2	II	18

target protein	mutagenesis	#	signal sequence	vector name	His-tag	TEV sequence	level of biosynthesis	gel no. Suppl. Fig.1AB	lane no.
**LH**	U493STOP	17	no	pMCSG93	no	C-term	1	I	22
		18	no	pMCSG92	C-term	C-term	1	I	18
		19	no	pMCSG53	N-term	N-term	1	IV	12
		20	no	pMCSG92	C-term	C-term	1	IV	16
		21	no	pMCSG93	no	C-term	3	IV	20
		22	no	pRSF	no	C-term	2	IV	22
**SH**	No	23	no	pMCSG92	C-term	C-term	1	I	10
		24	DsbA, N-term	vDsbA	C-term	no	2	I	8
		25	no	pMCSG93	no	C-term	2	I	24
		26	MalE, N-term	pMCSG92	C-term	C-term	2	II	9
		27	MalE, N-term	pMCSG93	no	C-term	1	II	19
		28	no	pMCSG53	N-term	N-term	3	III	5
		29	no	pRSF	no	C-term	1	III	25

gel I

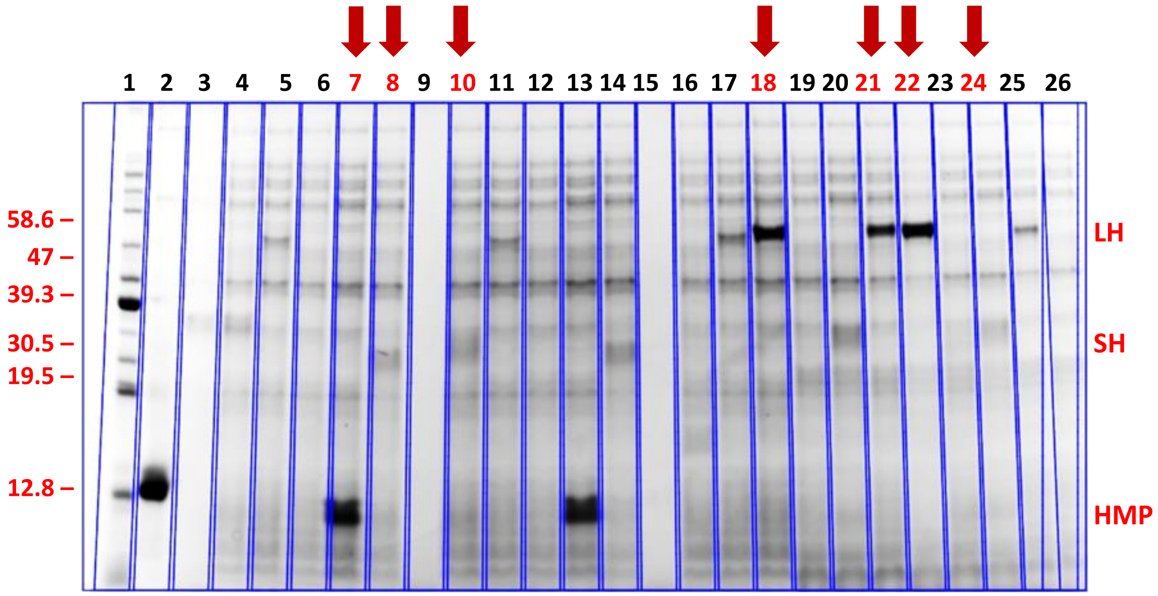

gel II

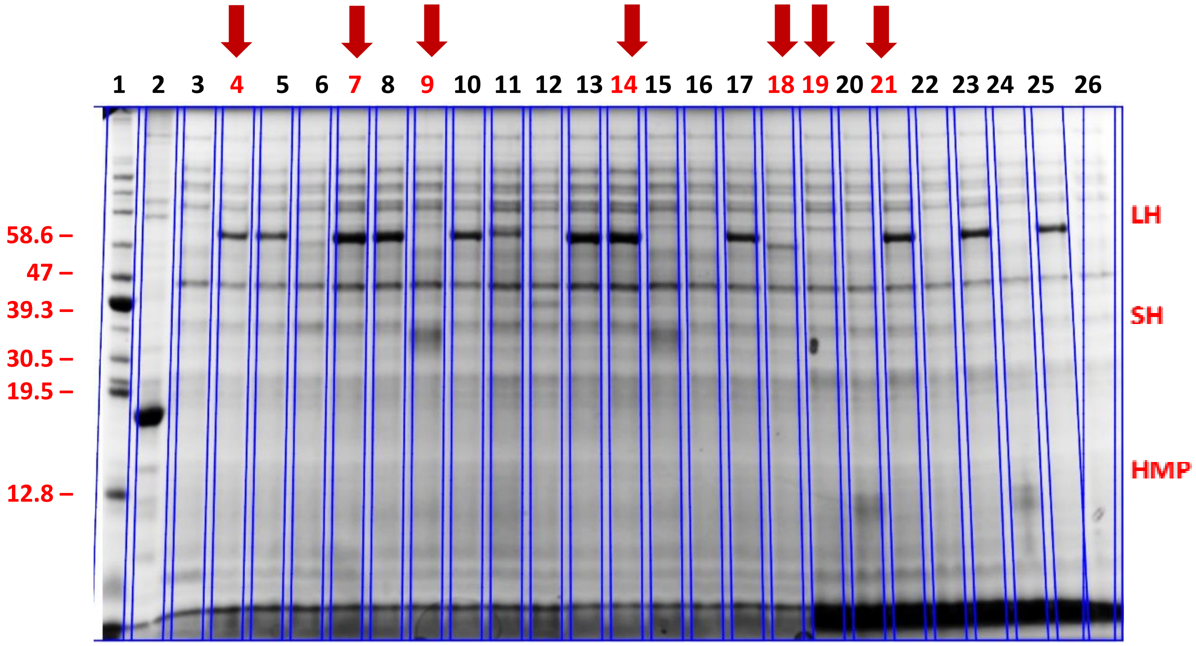

Suppl. Fig.1A. SDS-PAGE analysis of POI biosynthesis, obtained with various recombinant DNA constructs: gels I and II. Lanes corresponding to positive bacterial clones, which were selected for further experiments (Suppl. Tab.1), are marked with red arrows. Large subunit (LH), small subunit (SH) and hydrogenase maturation protease (HMP) abbreviations are placed corresponding to the predicted POIs migration in gel.

gel III

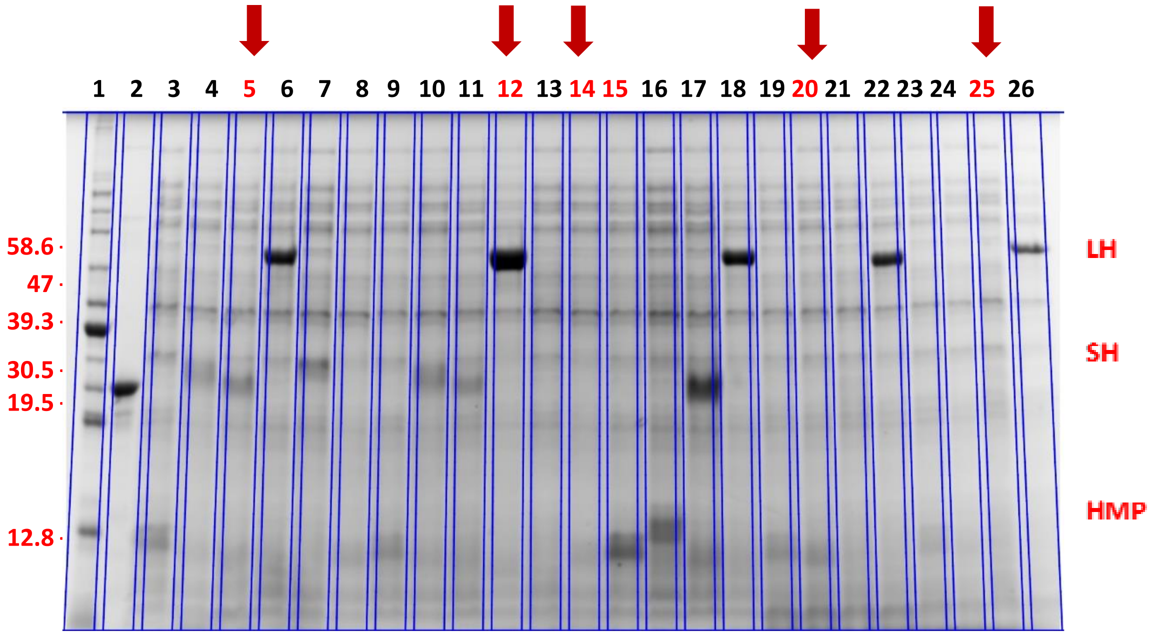

gel IV

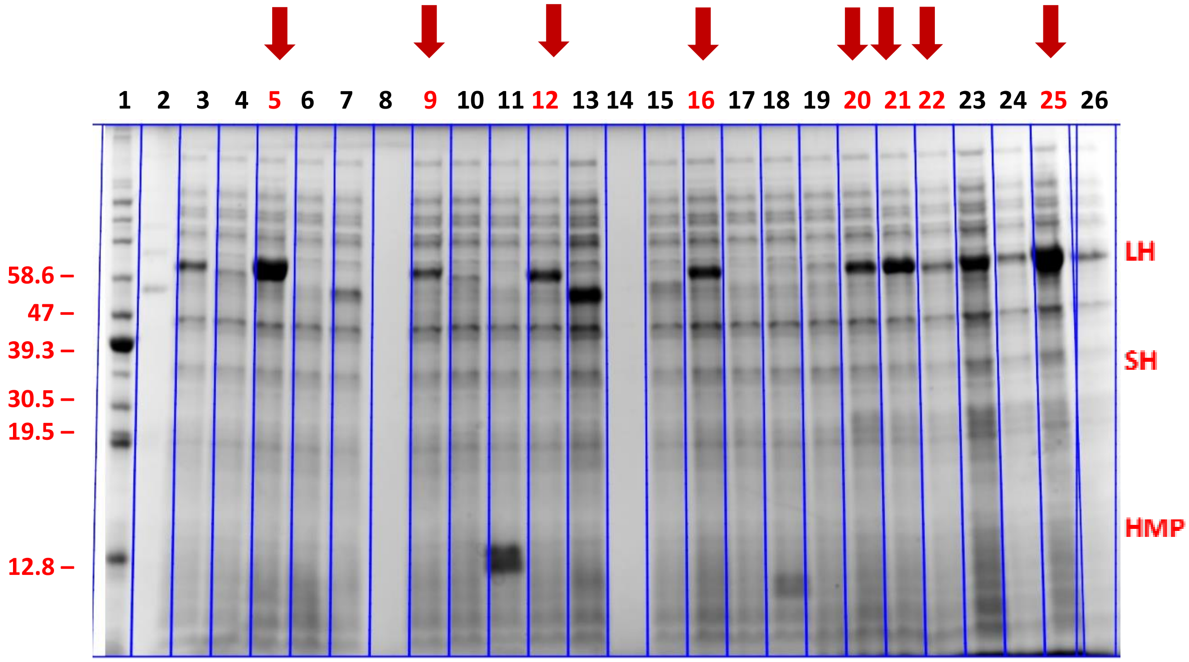

Suppl. Fig.1B. SDS-PAGE analysis of the POIs biosynthesis using various recombinant DNA constructs: gels III and IV. Lanes corresponding to positive bacterial clones, which were selected for further experiments (Suppl. Tab.1), are marked with red arrows. Large subunit (LH), small subunit (SH) and hydrogenase maturation protease (HMP) abbreviations are placed corresponding to the predicted POIs migration in gel.

1.2 Production of protein complexes via two genes layouts

1.2.1 PCR amplification

The main purpose of this stage was to produce inserts for assembling operons with RBS 514 or RBS 517 sequences. Thirty six PCR reactions were prepared with the previously confirmed constructs as DNA templates. PCR products were analyzed using agarose gel electrophoresis. Two PCR reactions – designed for SECIS element reconstruction – were negative.

1.2.2 Annealing and transformation

Thirty four specific PCR products were purified from an excess of dNTPs and subjected to incubation with T4 DNA polymerase. Operons consisting of two genes, with RBS 514 or 517, in various vectors, were assembled (LH-SH, LH-HMP, and SH-HMP) to result in twenty transformation reactions (Suppl. Tab.2).

Both kanamycin- and ampicillin-resistant colonies were observed after overnight incubation.

Suppl. Tab.2. Inserts and vectors for the designed operons.

vector	insert	5' to 3' arrangement	gel no. Suppl. Fig.2AB	lane
LH_STOP493_rbs517_v92	rbs514/517_HMP_STOP	LH → HMP →	VII	8
LH_STOP493_rbs517_v92	rbs514/517_DsbA_SH_His-tag, insert	LH → SH →	VI	10
HMP_v92	No	HMP →	VII	14
LH M493_rbs514_v92	rbs514/517_HMP_STOP	HMP → LH →	VIII	7
LH M493_rbs514_v92	SH_TEV, insert	LH → SH →	VII	18
LH M493_rbs514_v92	rbs514/517_DsbA_SH_His-tag, insert	LH → SH →	VIII	6

vector	insert	5' to 3' arrangement	gel no. Suppl. Fig.2AB	lane
LH STOP493_rbs517_v92,	SH_TEV, insert	LH → SH	VIII	10
v92	LH C493_TEV, insert	LH	VII	5
LH C493_rbs514_v53	DsbA_HMP_rbs514, insert	HMP → LH	V	6
LH C493_rbs514_v53	rbs514/517_DsbA_SH_His-tag, insert	LH → SH	V	7
LH C493_rbs517_v53	rbs514/517_HMP_STOP	LH → HMP	V	22
LH M493_rbs517_v53	DsbA_HMP_rbs514, insert	HMP → LH	V	5
LH M493_rbs517_v53	rbs514/517_HMP_STOP	LH → HMP	VIII	12
LH M493_rbs517_v53	rbs514/517_DsbA_SH_His-tag, insert	LH → SH	VIII	6
LH STOP493_rbs514_v53	DsbA_HMP_rbs514, insert	HMP → LH	VI	21
LH STOP493_rbs514_v53	rbs514/517_DsbA_SH_His-tag, insert	LH → SH	VII	10
SH_rbs514_ v53,	rbs514/517_HMP_STOP	HMP → SH	V	11
SH_rbs517_v53	rbs514/517_HMP_STOP	HMP → SH	V	12
v53	TEV_LH STOP493, insert	LH	VI	16
vDsba	DsbA_LH STOP493_His-tag, insert	LH	VIII	5
vDsba	DsbA_LH C493_His-tag, insert	LH	VII	6
vDsba	DsbA_HMP_rbs514, insert	HMP	VII	11
LH M493_rbs514_v93	rbs514/517_HMP_STOP	LH → HMP	V	21
LH M493_rbs514_v93	rbs514/517_DsbA_SH_His-tag, insert	LH → SH	VI	20
HMP_v93	No	HMP	VII	22
LH C493_rbs514_v93	SH_TEV, insert	LH → SH	VII	17
LH C493_rbs517_v93	rbs514/517_HMP_STOP	LH → HMP	V	26

vector	insert	5' to 3' arrangement	gel no. Suppl. Fig.2AB	lane
LH C493_rbs517_v93	rbs514/517_DsbA_SH_His-tag, insert	LH SH	VIII	22
LH M493_rbs514_v93	SH_TEV, insert	LH SH	VIII	21
v93	LH STOP493_TEV, insert	LH	VIII	3
LH STOP493_rbs517_vRSF	rbs514/517_HMP_STOP	LH HMP	VI	23
SH_rbs514_vRSF	rbs514/517_HMP_STOP	SH HMP	VII	25
SH_rbs517_vRSF	rbs514/517_HMP_STOP	SH HMP	VI	24
vRSF	TEV_LH_M493, insert	LH	V	18
vRSF	TEV_SHP, insert	SH	VI	19

1.2.3 Expression

Two bacterial colonies were picked from each agar well and grown on a small scale. After 18 hours from induction of the recombinant gene expression, bacteria were spun down. Pellets from one expression well per transformation were subjected to lysis and analyzed by polyacrylamide gel electrophoresis (Suppl. Fig.2AB). Differences in electrophoretic migration of the C493 (Gel VII, lane 17), M493 (Gel VII, lane 18), and STOP493 (Gel VII, lane 10), LH variants were clearly visible (Suppl. Fig.2). The differences in migration of the potentially processed and non-processed DsbA_LH, however, were not detected (Suppl. Fig. 2). Based on the results of SDS-PAGE analysis, thirty two recombinant clones with confirmed biosynthesis of the POIs were selected for plasmid isolation and DNA sequencing.

gel V

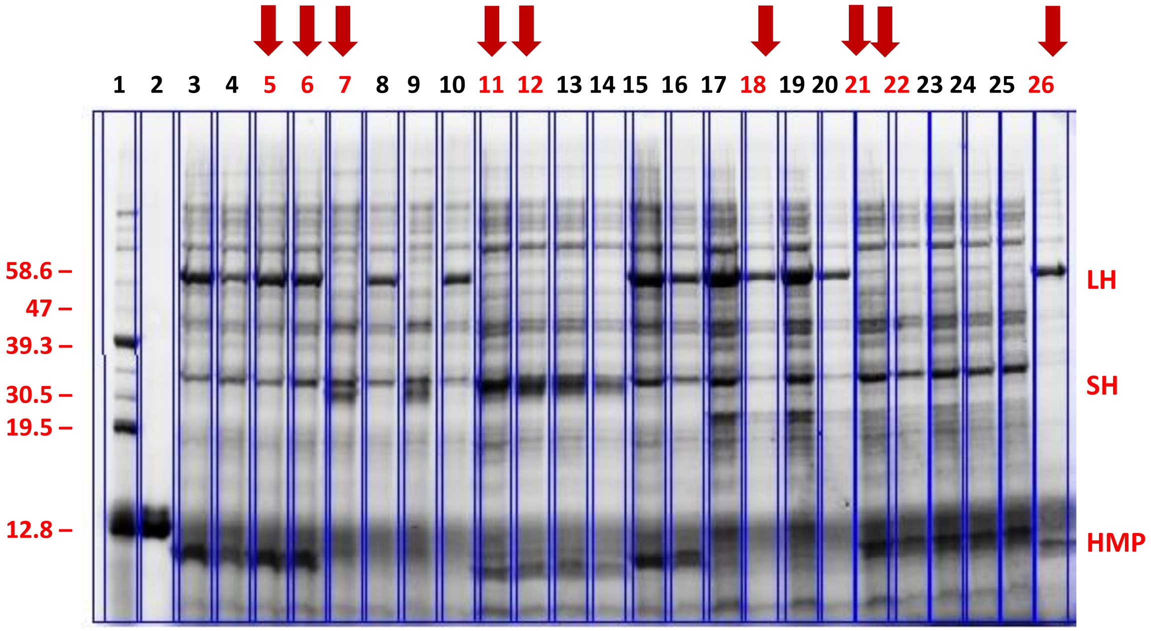

gel VI

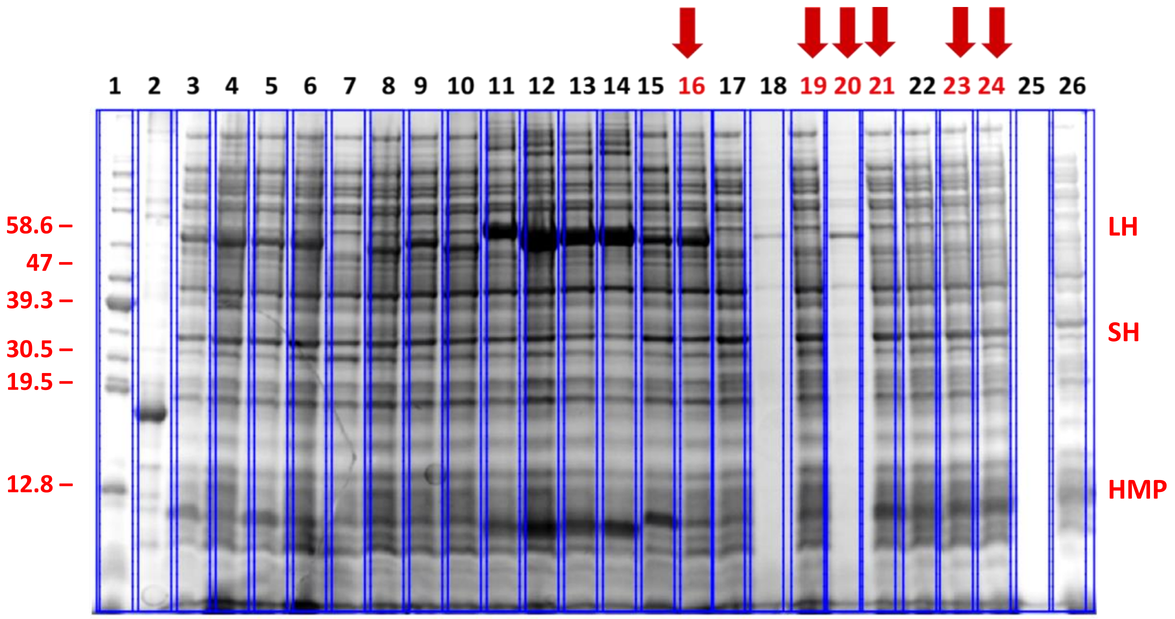

Suppl. Fig.2A. SDS-PAGE analysis of the POIs biosynthesis, obtained with the use of various recombinant operons: gels V and VI. Lanes corresponding to positive bacterial clones, which were selected for further experiments (Tab. 18), are marked with red arrows. Large subunit (LH), small subunit (SH) and hydrogenase maturation protease (HMP) abbreviations are placed corresponding to the predicted POIs migration in gel.

gel VII

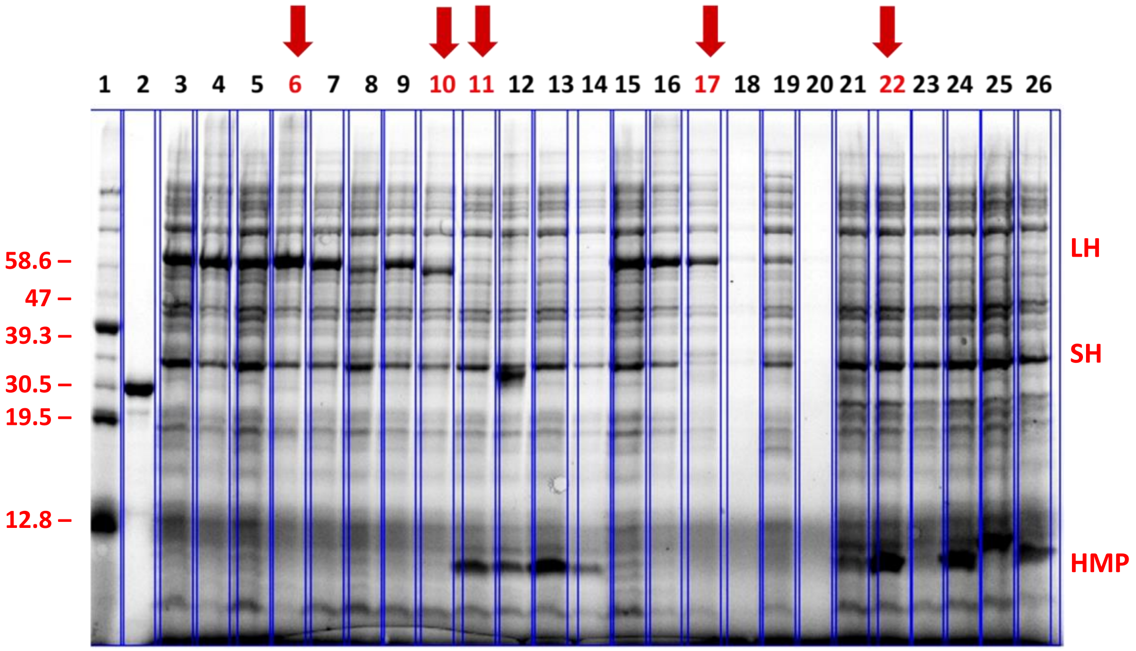

gel VIII

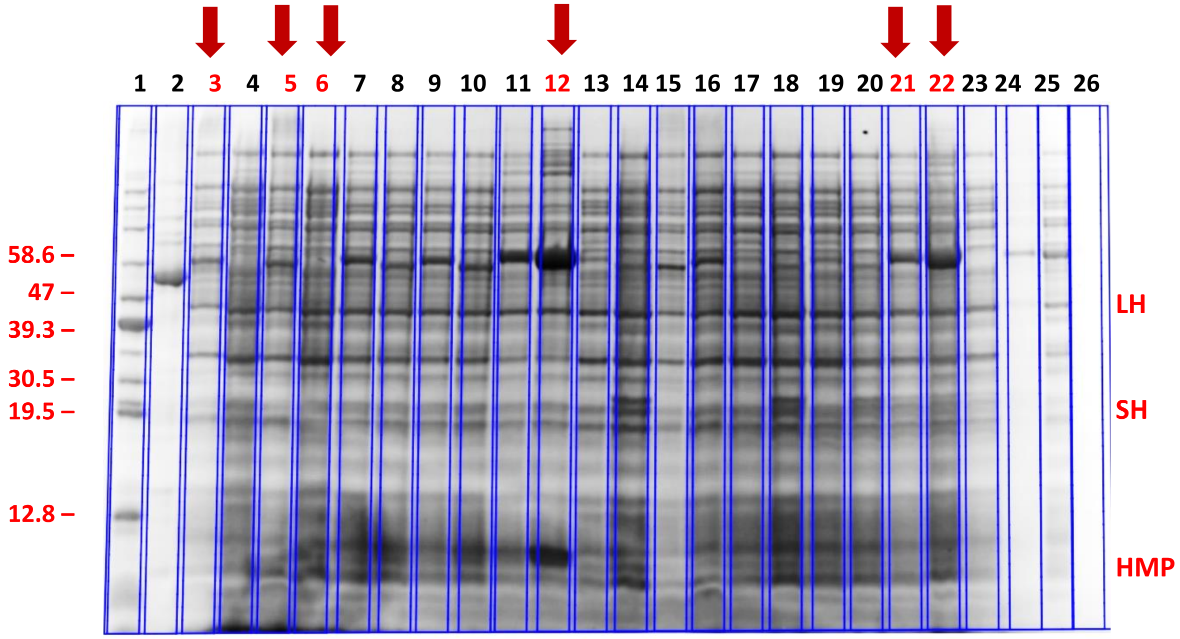

Suppl. Fig.2B. SDS-PAGE analysis of the POIs biosynthesis, obtained with the use of various recombinant operons: gels VII and VIII. Lanes corresponding to positive bacterial clones, which were selected for further experiments (Tab. 18), are marked with red arrows. Large subunit (LH), small subunit (SH) and hydrogenase maturation protease (HMP) abbreviations are placed corresponding to the predicted POIs migration in gel.

1.2.4 IMAC purification

Based on the results of the POI production screening, twenty five recombinant bacterial clones were chosen for IMAC purification experiments (Suppl. Tab.3). Most of the selected constructs code for two His-tagged proteins and so show two bands when analyzed by SDS-PAGE. Thus, two bands were detected in the analyzed samples and show rather the occurrence of both proteins than pairing of the subunits (Suppl. Fig.3; Suppl. Tab.3).

Suppl. Tab.3. Constructs chosen for IMAC purification.

Construct	gel no. (Suppl. Fig.3)	lane
LH STOP493_v53	IX	2
DsbA_LH C493_rbs514_DsbA_SH_v53	IX	3
LH C493_v92	IX	4
DsbA_LH STOP493_rbs514_DsbA_SH_v53	IX	5
LH C493_vDsbA	IX	6
LH M493_rbs514_SH_v92	IX	7
DsbA_LH M493_rbs517_DsbA_SH_v53	IX	8
LH STOP493_rbs517_SHP_v92	IX	9
LH M493_rbs514_DsbA_SH_v92	IX	10
SH_rbs517_HMP_STOP_v53	IX	11
LH_STOP493_rbs517_DsbA_SH_v92	IX	12
SH_rbs514_HMP_STOP_ v53	IX	13
LH STOP493_rbs517_SHP_v92	IX	14
LH C493_rbs517_HMP_STOP_v53	IX	15
DsbA_LH C493_rbs514_HMP_v53	IX	16
LH M493_rbs517_HMP_STOP_v53	IX	17
LH STOP493_v53	IX	18
HMP_v92	IX	19
LH M493_rbs514_SHP_v92	IX	20
SH_rbs514_HMP_STOP_ v53	IX	21
LH M493_rbs514_DsbA_SH_v93	IX	22
SH_rbs514_HMP_vRSF	IX	23
LH C493_rbs517_DsbA_SH_His-tag_v93	IX	24
SH_rbs514_HMP_vRSF	IX	25
LH C493_rbs514_STOP_v92	IX	26

gel IX

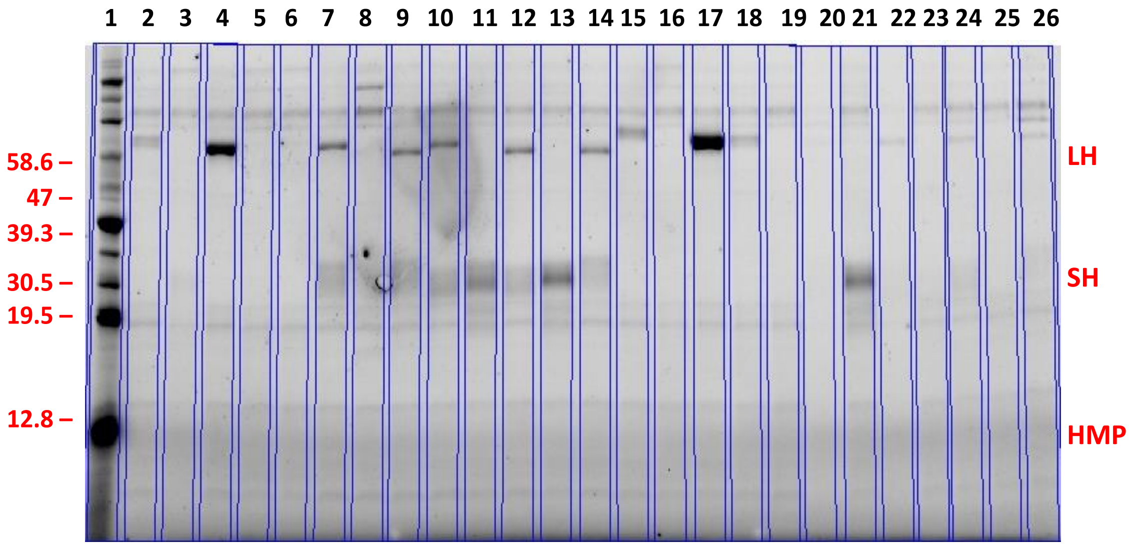

Suppl. Fig.3. IMAC purification of the selected recombinant protein constructs (Suppl. Tab.3). Large subunit (LH), small subunit (SH) and hydrogenase maturation protease (HMP) abbreviations are placed corresponding to the predicted POIs migration in gel.

1.3 Production of hydrogenase complex via co-expression of three-piece operon elements

Native *Dmb* is derived from a strictly anaerobic, Gram-negative bacterium. Hydrogenases, *per se*, are vulnerable to oxidizing species. Thus, it is legitimate to apply anaerobic growth conditions for hydrogenase biosynthesis. In fact, such an approach was previously used by others to express heterologous hydrogenases genes from organisms such as *Pyrococcus furiosus* (Song et al., 2019; Sun et al., 2010), *Caldanaerobacter subterranus* (Kelly et al., 2015), *Clostridium pasteurianum* (Kuchenreuther et al., 2010) or to enhance the activity of native *E. coli* hydrogenases (del Barrio et al., 2019; Sawers et al., 2004). Of three-piece operons, both LH and SH carry elements susceptible to oxidation. Although the catalytic center of LH is buried in the protein, it can be poisoned by oxygen species. Additionally, Fe-S clusters, connecting the catalytic center with the surface of the enzyme, can be inactivated by oxidation (Lukey et al., 2011; Stripp et al., 2014; Vincent et al., 2005). In order to test the hypothesis on favorable anoxic conditions, constructs comprising one or two gene layouts were simultaneously co-expressed in anaerobic and aerobic conditions.

7.3.1 Annealing and transformation

Eight co-transformations were performed, (Suppl.Tab.4). Each transformant was grown in duplicate. Cells were cultured at 37°C/190 rpm until A_{600} = 1.1 was reached. Cultures were cooled in an ice bath to 20°C, upon induction, additional phosphate (40 mM) and glucose (27.5 mM) were added, and one of each transformant duplicate culture was moved to an argon purged shaker. Cultures were grown at 18°C/150 rpm under argon purging and 18°C/190 rpm under aerobic conditions overnight.

Suppl. Tab.4. Constructs aligned for co-expression. Constructs marked in red manifested a slow growth rate and two projects with HMP_v92 proved to be toxic upon induction (cell lysis).

**construct I**	**construct II**	**antibiotic**
DsbA_LH M493_rbs517_DsbA_SH_v53	no	Am
LH M493_rbs514_SH_v92	HMP_v93	Am/Kn
LH M493_rbs514_DsbA_SH_v92	HMP_v93	Am/Kn
LH C493_rbs517_DsbA_SH_His-tag_v93	HMP_v92	Am/Kn
LH C493_rbs514_STOP_v93	HMP_v92	Am/Kn
SH_rbs517_HMP_STOP_v53	LH M493_vRSF	Am/Kn
SH_rbs517_HMP_STOP_v53	LH M493_vRSF	Am/Kn
SH_rbs517_HMP_STOP_v53	LH STOP493_vRSF	Am/Kn

1.3.2 Expression and IMAC analysis

After lysis, total protein samples were taken and IMAC purification was performed. Samples from cultures grown in aerobic (O2) and anaerobic (~~O2~~) conditions were analyzed side by side (Suppl. Tab.5), (Suppl. Fig.4). Constructs with LH U493C and HMP appeared to be especially toxic upon expression. No significant changes in gene expression and protein production were observed under anaerobic conditions.

Suppl. Tab.5. Set of co-expressed constructs grown in aerobic (O2) and anaerobic (~~O2~~) conditions.

conditions	lane no. (Suppl. Fig.4)	co-expressed constructs
O2	3	DsbA_LH M493_rbs517_DsbA_SH_v53
O2	5	LH M493_rbs514_SH_v92 + HMP_v93
O2	7	LH M493_rbs514_DsbA_SH_v92 + HMP_v93
O2	9	SH_rbs517_HMP_STOP_v53 + LH M493_vRSF
O2	11	SH_rbs517_HMP_STOP_v53 + LH M493_vRSF
O2	13	SH_rbs517_HMP_STOP_v53 + LH STOP493_vRSF
O2	16	DsbA_LH M493_rbs517_DsbO2_SH_v53
O2	18	LH M493_rbs514_SH_v92 + HMP_v93
O2	20	LH M493_rbs514_DsbA_SH_v92 + HMP_v93
O2	22	SH_rbs517_HMP_STOP_v53 + LH M493_vRSF
O2	24	SH_rbs517_HMP_STOP_v53 + LH M493_vRSF
O2	26	SH_rbs517_HMP_STOP_v53 + LH STOP493_vRSF
~~O2~~	2	DsbA_LH M493_rbs517_DsbA_SH_v53
~~O2~~	4	LH M493_rbs514_SH_v92 + HMP_v93
~~O2~~	6	LH M493_rbs514_DsbA_SH_v92 + HMP_v93
~~O2~~	8	SH_rbs517_HMP_STOP_v53 + LH M493_vRSF
~~O2~~	10	SH_rbs517_HMP_STOP_v53 + LH M493_vRSF
~~O2~~	12	SH_rbs517_HMP_STOP_v53 + LH STOP493_vRSF
~~O2~~	15	DsbA_LH M493_rbs517_DsbA_SH_v53
~~O2~~	17	LH M493_rbs514_SH_v92 + HMP_v93
~~O2~~	19	LH M493_rbs514_DsbA_SH_v92 + HMP_v93
~~O2~~	21	SH_rbs517_HMP_STOP_v53 + LH M493_vRSF
~~O2~~	23	SH_rbs517_HMP_STOP_v53 + LH M493_vRSF
~~O2~~	25	SH_rbs517_HMP_STOP_v53 + LH STOP493_vRSF

gel X

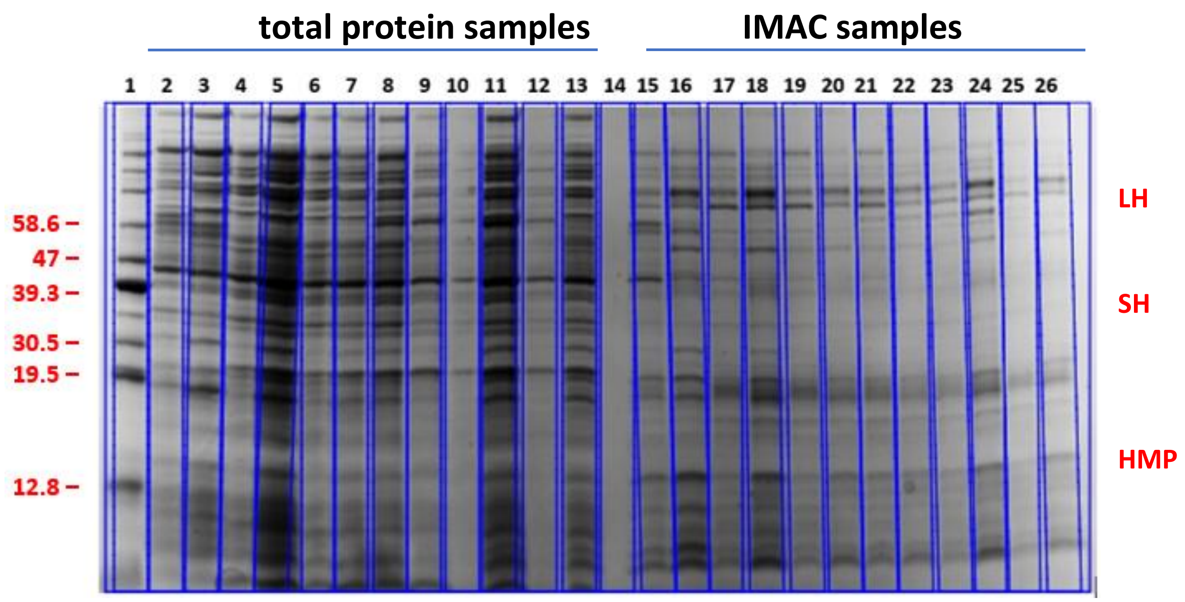

Suppl. Fig.4. SDS-PAGE analysis of total protein samples and fractions obtained after IMAC purification: gel X. All the investigated bacterial cultures contained three elements of the recombinant *Dmb* hydrogenase operon. Large subunit (LH), small subunit (SH) and hydrogenase maturation protease (HMP) abbreviations are placed corresponding to the predicted POIs migration in gel.

.

C493-HMP constructs appeared to be especially toxic. No significant changes in expression and protein production were observed under anaerobic conditions.

1.4 Production of hydrogenase complex via *in vitro* assembly

Due to the high toxicity of certain protein constructs, or very low growth rate of some recombinant clones chosen for co-expression experiments, another set of constructs was used to transform *E. coli*. Bacterial cultures were grown simultaneously in anaerobic and aerobic conditions as before. Additionally, the growth medium was supplemented with 50 μM of $NiSO_4$ upon induction. The obtained cell pellets (containing one or two hydrogenase subunits) were mixed *in vitro*, subjected to lysis, and further analyzed.

1.4.1 Expression and IMAC analysis

Small scale expression of the selected, previously verified recombinant constructs was performed. For the mixing experiment, 1 ml of culture was added from each desired well

(Suppl. Tab.6). Then, the mixing plate was spun down at 3250 rpm for 25 min, the supernatant was discarded and, if necessary, a third bacterial culture was added. Cell lysis and IMAC purification were performed.

Suppl. Tab.6. Construct pairings for *in vitro* assembly experiments. Constructs grown in aerobic (O2) and anaerobic (~~O2~~) conditions. ND: not determined.

**construct 1**	**construct 2**	**construct 3**	**gel no. Suppl. Fig.5**	**lane O2**	**lane ~~O2~~**
HMP_v53	LH C493_v92	no	XI	4	3
HMP_v53	LH C493_v92	SH_v92	XI	6	5
HMP_v53	LH C493_v92	SH_v53	XI	8	7
HMP_v53	LH C493_v53	no	XI	10	9
HMP_v53	LH C493_v53	SH_v92	XI	12	11
HMP_v53	LH C493_v53	SH_v53	XI	14	13
SH_v92	LH C493_rbs517_HMP_STOP_v53	no	XI	16	15
SH_v92	LH C493_v92	no	XI	18	17
SH_v92	LH C493_v53	no	XI	20	19
SH_v53	LH C493_v53	no	XI	22	21
SH_v53	LH C493_rbs517_HMP_STOP_v53	no	XI	24	23
SH_v53	LH C493_v92	no	XI	26	25
HMP_vRSF	LH C493_v53	SH_v92	XII	3	ND
HMP_vRSF	LH C493_v53	SH_v53	XII	4	ND
HMP_v92	LH M493_v53	no	XII	5	ND
HMP_v92	LH M493_v92	SH_v53	XII	6	ND
HMP_v92	LH M493_v53	SH_v92	XII	7	ND
HMP_v92	LH C493_v93	no	XII	8	ND
HMP_v92	LH M493_v53	SH_v53	XII	9	ND
HMP_v92	LH C493_v93	SH_v92	XII	10	ND
HMP_v92	LH M493_v92	no	XII	11	ND
HMP_v92	LH C493_v93	SH_v53	XII	12	ND
HMP_v92	LH M493_v92	SH_v92	XII	13	ND
HMP_v92	LH M493_rbs514_SH_v92	no	XII	14	ND
SH_v92	LH C493_v93	no	XII	15	ND
HMP_vDsbAss	LH C493_vDsbA	no	XII	16	ND

construct 1	construct 2	construct 3	gel no. Suppl. Fig.5	lane O2	lane ~~O2~~
SH_v92	LH M493_v92	no	XII	17	ND
HMP_vDsbAss	LH C493_vDsbA	SH_vDsbA	XII	18	ND
SH_v92	LH M493_v53	no	XII	19	ND
HMP_vDsbAss	LH M493_vDsbA	no	XII	20	ND
SH_v53	LH M493_v53	no	XII	21	ND
HMP_vDsbAss	LH M493_vDsbA	SH_vDsbA	XII	22	ND
SH_v53	LH M493_v92	no	XII	23	ND
HMP_vDsbAss	DsbA_LH M493_rbs517_DsbA_SH_v53	no	XII	24	ND
SH_v53	LH C493_v93	no	XII	25	ND
SHP_vDsbA	LH M493_vDsbA	no	XII	26	ND
DsbA_LH C493_rbs514_DsbA_HMP_v53	no	no	XIII	4	3
LH C493_rbs517_HMP_STOP_v53	no	no	XIII	6	5
LH C493_v92	no	no	XIII	8	7
SH_v92	no	no	XIII	10	9
HMP_v53	no	no	XIII	12	11
SH_vDsbA	no	no	XIII	14	13
SH_v53	no	no	XIII	16	15
LH C493_v53	no	no	XIII	18	17
LH C493_vDsbA	no	no	XIII	20	19
HMP_v92	no	no	XIII	21	ND
LH M493_v53	no	no	XIII	22	ND
LH M493_rbs514_SH_v92	no	no	XIII	23	ND
LH M493_v92	no	no	XIII	24	ND
DsbA_LH M493_rbs517_DsbA_SH_v53	no	no	XIII	25	ND
HMP_vDsbA	no	no	XIII	26	ND
SH_vDsbA	DsbA_LH C493_rbs514 _DsbA_HMP_v53	no	XIV	3	4
LH C493_v93	no	no	XIV	5	ND
LH C493_rbs514_STOP_v93	HMP_v92	no	XIV	6	ND
HMP_vRSF	No	no	XIV	7	ND
SHP_vDsbA	LH C493_vDsbA	no	XIV	8	10
LH C493_rbs517_DsbA_SH_His-tag_v93	HMP_v92	no	XIV	9	ND
LH C493_rbs514_STOP_v93	HMP_v92	no	XIV	11	ND

gel XI

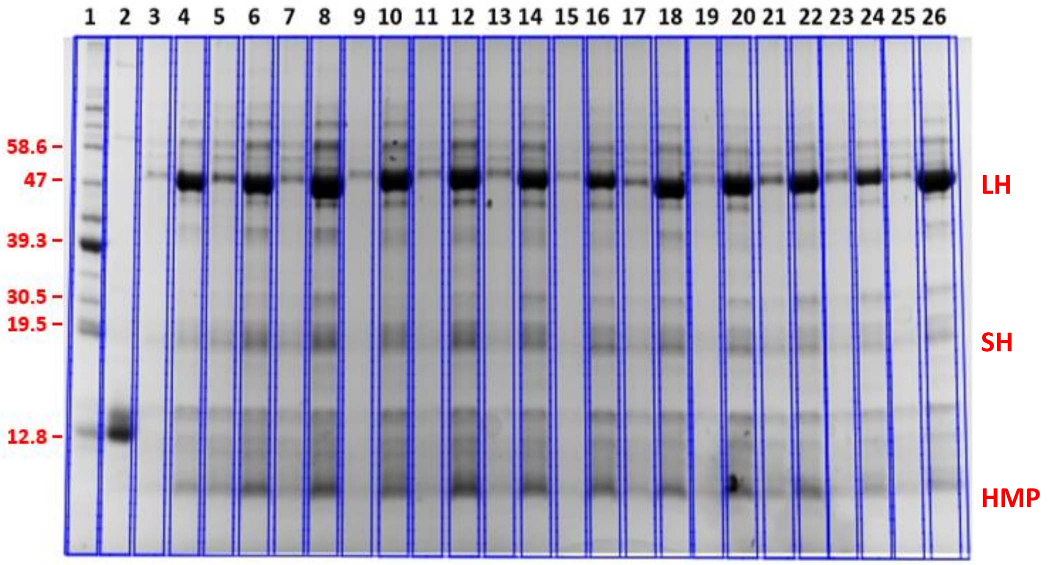

gel XII

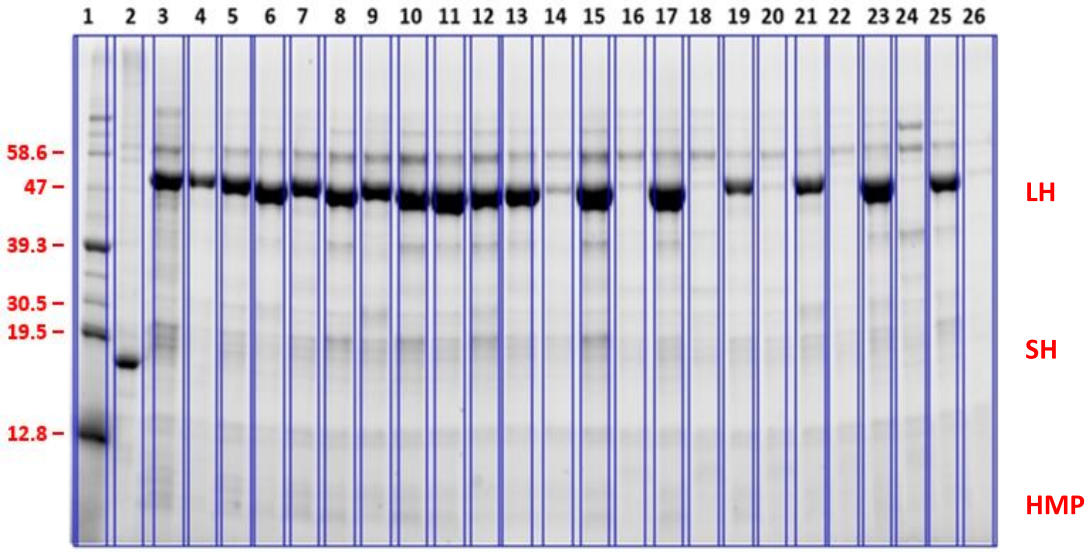

Suppl. Fig.5A. SDS-PAGE analysis of the eluted protein fractions after IMAC purification of the cell lysates, obtained after *in vitro* mixing experiments (Suppl. Tab.6): gels XI and XII. Large subunit (LH), small subunit (SH) and hydrogenase maturation protease (HMP) abbreviations are placed corresponding to the predicted POIs migration in gel.

gel XIII

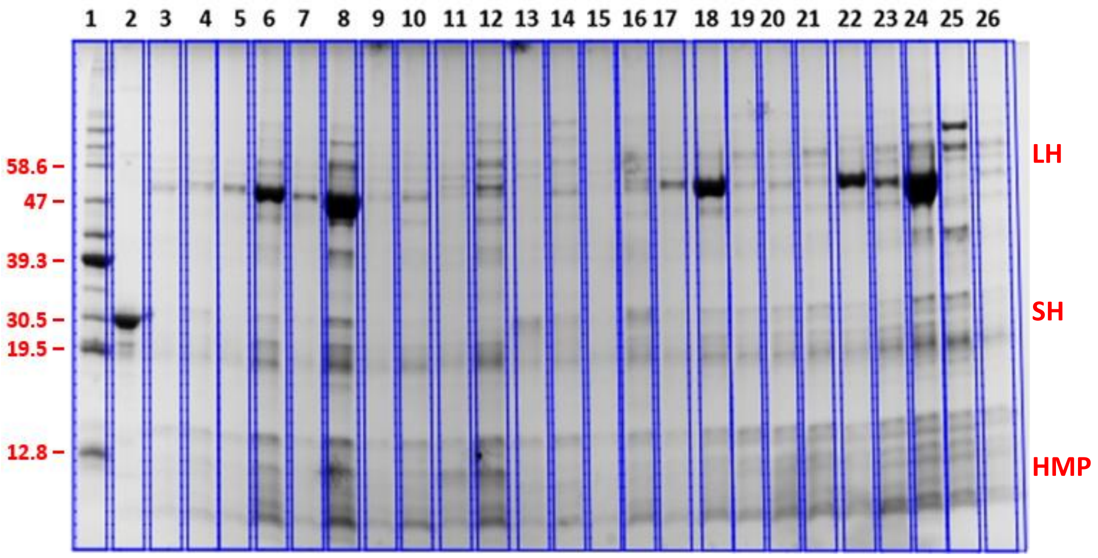

gel XIV

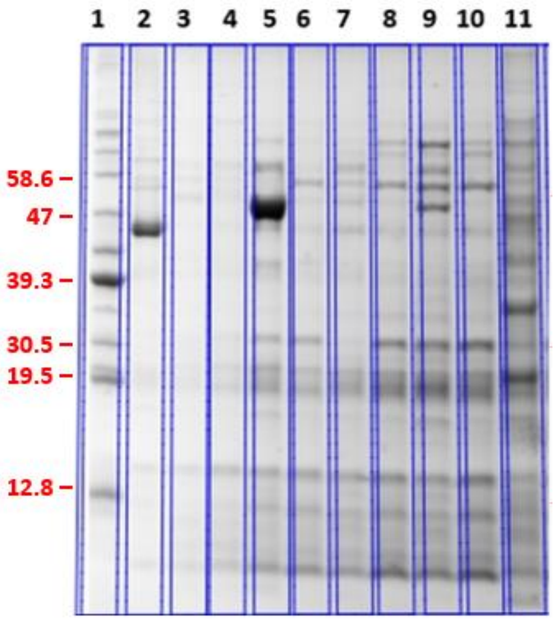

Suppl. Fig.5B. SDS-PAGE analysis of the eluted protein fractions after IMAC purification of the cell lysates, obtained after *in vitro* mixing experiments (Suppl. Tab.5): gels XIII and XIV. Large subunit (LH), small subunit (SH) and hydrogenase maturation protease (HMP) abbreviations are placed corresponding to the predicted POIs migration in gel.

1.5 Periplasmic transport signal modifications and large subunit truncation

Based on the previous results, it was hypothesized that the periplasmic transportation signals DsbA and MalE are probably not processed. For the previous experiments, only partial DsbA and MalE signal sequences were employed. Thus, it was believed that the implementation of longer versions could improve periplasmic transport performance.

The main purpose of this experiment was to produce inserts for the periplasmic transport with a longer DsbA signal (DsbA2: SASAA instead of DsbA: SASA), longer MalE signal (MalE2: SALAKI), and constructs for the DsbA vector with kanamycin resistance. Additionally, inserts with the large subunit truncated at position 499 (LH (all)493_STOP499; mimicking the effect of proteolysis with HMP), as well as large hydrogenase subunits modified with the SECIS element (LH STOP493_499A, LH STOP493_499H), were produced.

1.5.1 PCR amplification

Twenty five PCR reactions were prepared. The obtained PCR products were analyzed using agarose gel electrophoresis.

1.5.2 Annealing and transformation

Specific PCR products were purified from the excess of dNTPs and subjected to reaction with T4 DNA polymerase. LIC-treated reactions were annealed and transformed, resulting in twenty two transformation experiments (Suppl. Tab.7). Both kanamycin and ampicillin-resistant clones were observed after overnight incubation except for: *i*) LH C493_STOP499_v92 (no colonies), *ii*) LH M493_STOP499_v92 (one colony), *iii*) LH STOP493_STOP499_v92 (two colonies).

Suppl. Tab.7. Alignment of inserts and vectors for LIC reaction and *E. coli* transformation.

**vector**	**Insert**	**gel no. Suppl. Fig.6**	**lane**
SH_vMalE2	No	XVI	25
v92	LH M493_499STOP_TEV, insert	XVI	12
v92	LH STOP493_499STOP_TEV, insert	XVI	15
v93	LH M493_499STOP_TEV, insert	XVI	11
v93	LH C493_499STOP_TEV, insert	XVI	13
v93	LH STOP493_499STOP_TEV, insert	XV	15
vDsbA	DsbA2_SH, insert	XV	2
vDsbA	DsbA2_LH M493_STOP_His-tag, insert	XV	4
vDsbA	DsbA2_LH M493_499STOP_His-tag, insert	XV	5
vDsbA	DsbA2_LH C493_STOP_His-tag, insert	XV	6
vDsbA	DsbA2_LH STOP493_STOP_His-tag, insert	XV	8
vDsbA	DsbA2_LH C493_499STOP_His-tag, insert	XVI	14
vDsbA	DsbA2_LH STOP493_499STOP_His-tag, insert	XVI	19
vDsbA	DsbA2_HMP_STOP_His-tag, insert	XV	10
vDsbA(Kn)	MalE_SH_His-tag, insert	XVI	10
vMalE	MalE2_HMP_His-tag, insert	XV	3
MalE	MalE2_LH M493_STOP_His-tag, insert	XV	12
vMalE	MalE2_LH C493_STOP_His-tag, insert	XV	14
vMalE	MalE2_LH STOP493_STOP_His-tag, insert	XV	16
vRSF	TEV_LH M493_499STOP, insert	XVI	3
vRSF	TEV_LH C493_499STOP, insert	XVI	5
vRSF	TEV_LH STOP493_499STOP, insert	XVI	7

1.5.3 Expression analysis

Two colonies were picked from each agar well (if possible) and grown in 1 ml of LB. After 18 hours from IPTG induction, the cells were spun down and lysed. Samples with a volume of 4 µl were taken after lysis. The collected samples were analyzed by SDS- PAGE (Suppl. Fig.6). Most of the LH constructs investigated in this experiment do not have a His-tag (Suppl. Tab.7). Thus, IMAC analysis was not performed. All the obtained recombinant DNA constructs were sequenced.

gel XV

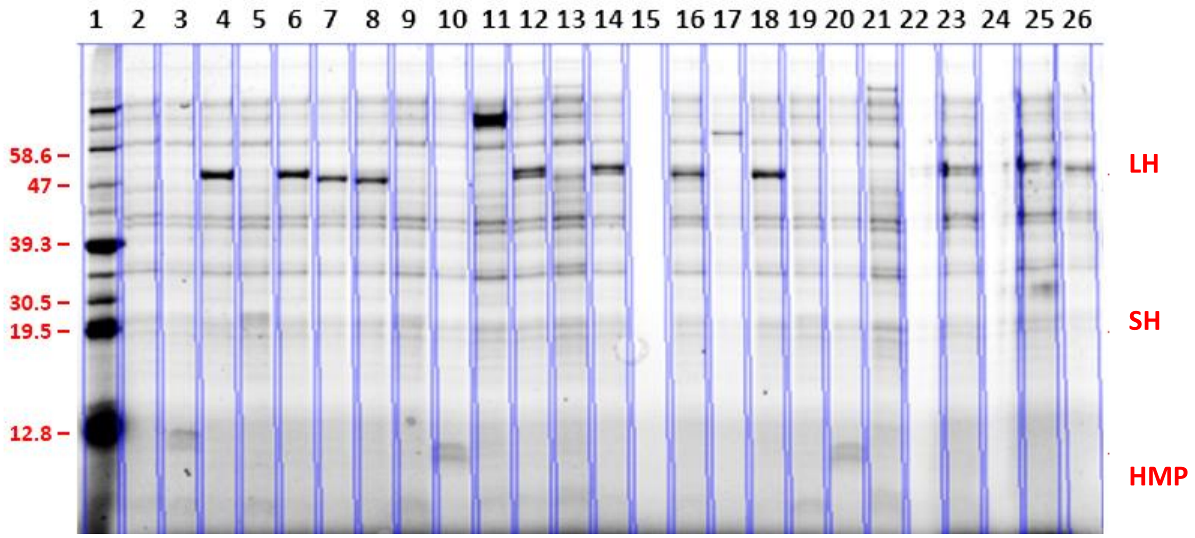

gel XVI

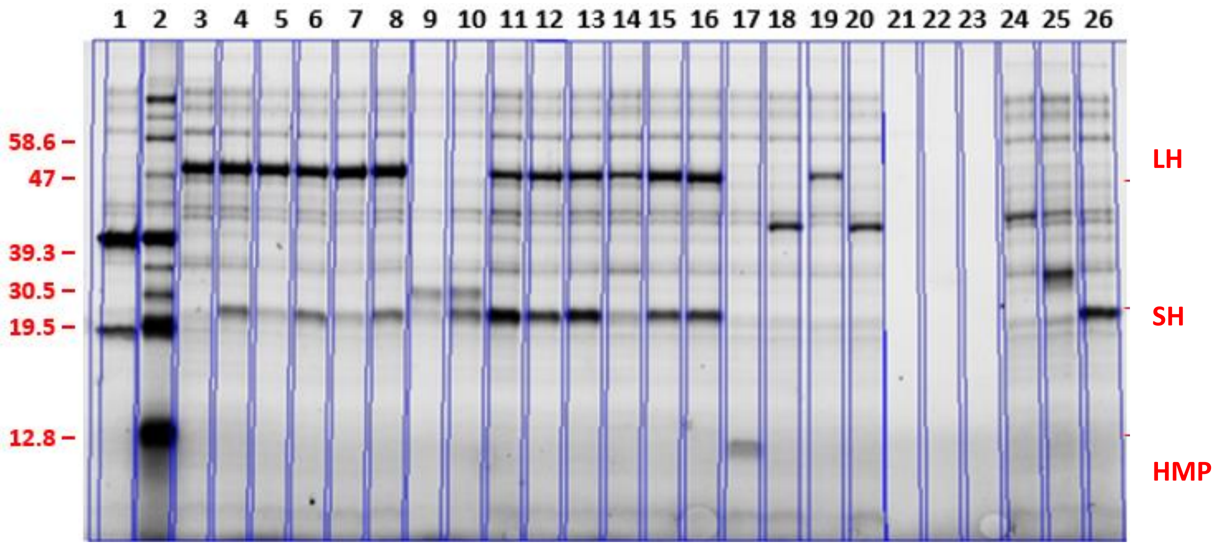

Suppl. Fig.6. SDS-PAGE analysis of the cell lysates, obtained after expression experiment with periplasmic signaling (Suppl. Tab.7): gels XV and XVI. Large subunit (LH), small subunit (SH) and hydrogenase maturation protease (HMP) abbreviations are placed corresponding to the predicted POIs migration in gel.

1.6 Production of hydrogenase complex via co-expression of two-piece operon elements

The complicated maturation process of [NiFe] H2ases includes the activity of specific hydrogenase maturation protease. Only after cleavage of LH by HMP can the large and small subunits associate, and probably afterward be fitted into its typical cellular environment (Lacasse & Zamble, 2016). However, HMP can act only in the aftermath of several maturation steps (Khorasani-Motlagh et al., 2019; Pinske et al., 2019; Theodoratou et al., 2005). It was hypothesized that producing the LH subunit without the endoproteolytically cleavable C-terminal extension could simplify complex maturation and subunit assembly (Fig.31).

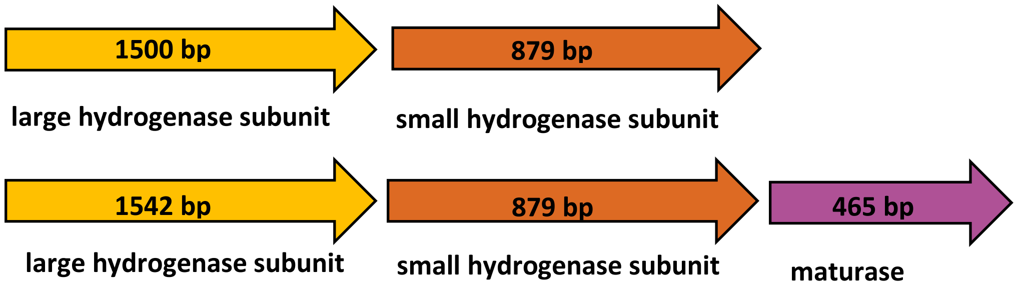

Suppl. Fig.7. Layouts of a two-piece operon with truncated hydrogenase (top) and three-piece operon (bottom).

1.6.1 Annealing and transformation

For co-expression, a set of SH and LH variants were chosen. The selected variants were: *i*) periplasmic SH (shorter DsbA version; SASA, with kanamycin resistance), *ii*) SH without periplasmic transport signaling, expressed from vector 53 (ampicillin resistance), *iii*) LH (all)493 truncated at position 499 or *iv*) LH (all)493 in an operon with HMP. All variants were grown in duplicate for aerobic and anaerobic conditions (under argon).

E. coli cells were co-transformed with plasmids chosen for co-expression experiments. Bacteria after transformation were plated on agar plates with corresponding antibiotics. Co-transformant alignments are presented in Suppl. Tab.8.

Suppl. Tab.8. Alignment of constructs selected for co-expression in aerobic (O2) and anaerobic (~~O2~~) conditions. Gel and lane numbers from corresponding SDS-PAGE analyses are noted. All DNA constructs presented in the table were sequenced.

**construct I**	**construct II**	**gel no. Suppl. Fig.8AB**	**lane O2**	**lane ~~O2~~**
SH_v53	no	XVII	3,4	15,16
No	DsbA2_LH M493_STOP_vDsbA	XVII	5	17
No	DsbA2_LH C493_STOP_vDsbA	XVII	6	18
MalE_SH_vDsbA (kan)	DsbA2_LH M493_STOP_vDsbA	XVII	7	19
MalE_SH_vDsbA (kan)	DsbA2_LH C493_STOP_vDsbA	XVII	8	20
SH_v53	LH M493_rbs514_HMP_STOP_v93	XVII	9	21
SH_v53	LH C493_rbs517_HMP_STOP_v93	XVII	10	22
No	LH M493_rbs514_HMP_STOP_v93	XVII	11	23
No	LH C493_rbs517_HMP_STOP_v93	XVII	12	24
MalE_SH_vDsbA (kan)	no	XVII	13,14	25,26
No	DsbA2_LH STOP493_STOP_vDsbA	XVIII	5	17
No	DsbA2_LH M493_499STOP_vDsbA	XVIII	6	18
MalE_SH_vDsbA (kan)	DsbA2_LH STOP493_STOP_vDsbA	XVIII	7	19
MalE_SH_vDsbA (kan)	DsbA2_LH M493_499STOP_vDsbA	XVIII	8	20
SH_v53	LH M493_499STOP_vRSF	XVIII	9	21
SH_v53	LH C493_499STOP_vRSF	XVIII	10	22
no	LH M493_499STOP_vRSF	XVIII	11	23
no	LH C493_499STOP_vRSF	XVIII	12	24
no	DsbA2_LH C493_499STOP_vDsbA	XIX	5	16
no	DsbA2_LH STOP493_499STOP_vDsbA	XIX	6	17
MalE_SH_vDsbA (kan)	DsbA2_LH C493_499STOP_vDsbA	XIX	7	18
MalE_SH_vDsbA (kan)	DsbA2_LH STOP493_499STOP_vDsbA	XIX	8	19
SH_v53	LH STOP493_499STOP_vRSF	XIX	9	20

SH_v53	LH M493_499STOP_v93	XIX	10	21
no	LH STOP493_499STOP_vRSF	XIX	11	22
no	LH M493_499STOP_v93	XIX	12	23
no	DsbA_LH M493_rbs517_DsbA_HMP_v53	XX	5	17
no	DsbA_LH C493_rbs514_DsbA_HMP_v53	XX	6	18
MalE_SH_vDsbA (kan)	DsbA_LH M493_rbs517_DsbA_HMP_v53	XX	7	19
MalE_SH_vDsbA (kan)	DsbA_LH C493_rbs514_DsbA_HMP_v53	XX	8	20
SH_v53	LH C493_499STOP_v93	XX	9	21
SH_v53	LH STOP493_499STOP_v93	XX	10	22
no	LH C493_499STOP_v93	XX	11	23
no	LH STOP493_499STOP_v93	XX	12	24

1.6.2 Expression analysis

Two colonies were picked from each agar well (if possible) and grown in 1 ml of LB. After 18 hours from IPTG induction, cells were spun down and lysed. Samples with a volume of 4 µl were taken after lysis. The collected samples were analyzed by SDS- PAGE (Suppl. Fig.8). Most of the LH constructs investigated in this experiment are truncated and do not have a His-tag (Suppl. Tab.8). Thus, IMAC analysis was not performed. All the obtained recombinant DNA constructs were sequenced.

gel XVII

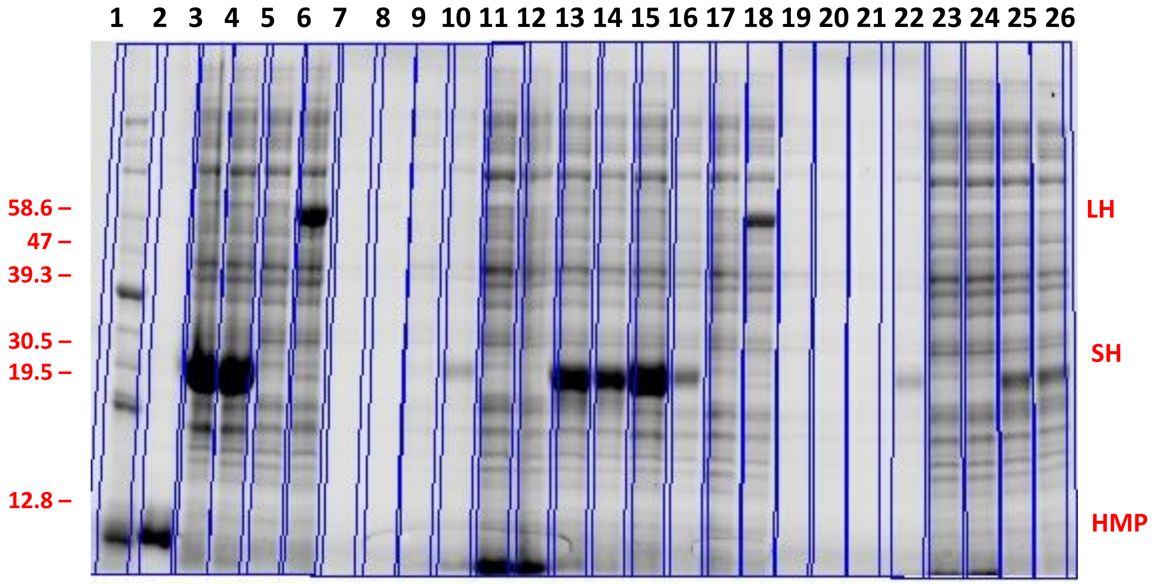

gel XVIII

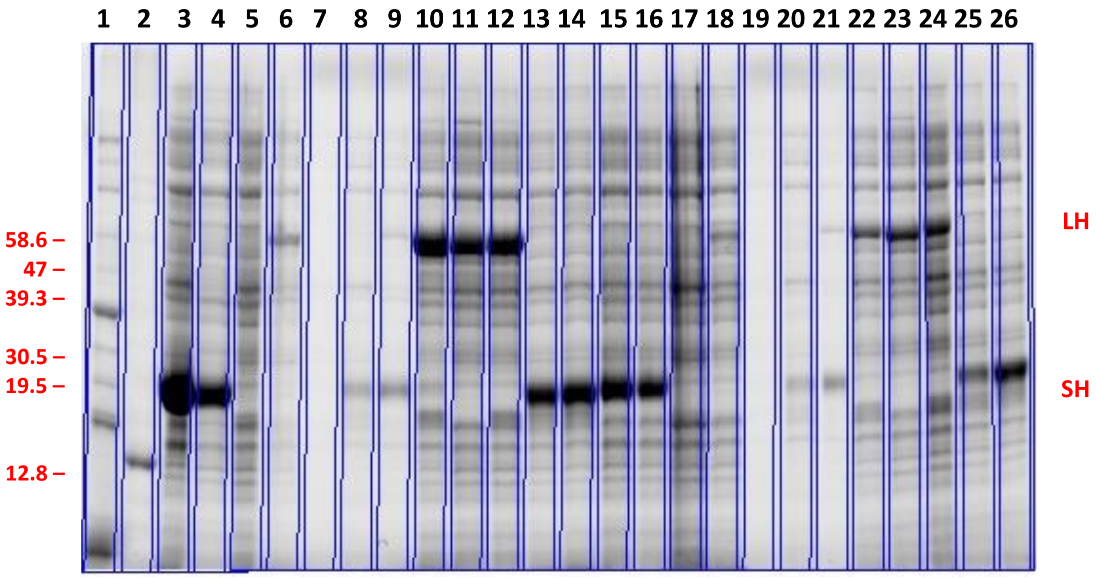

Suppl. Fig.8A. SDS-PAGE analysis of the cell lysates obtained after co-expression experiments (Suppl. Tab.8): gels XVII and XVIII. Large subunit (LH), small subunit (SH) and hydrogenase maturation protease (HMP) abbreviations are placed corresponding to the predicted POIs migration in gel.

gel XIX

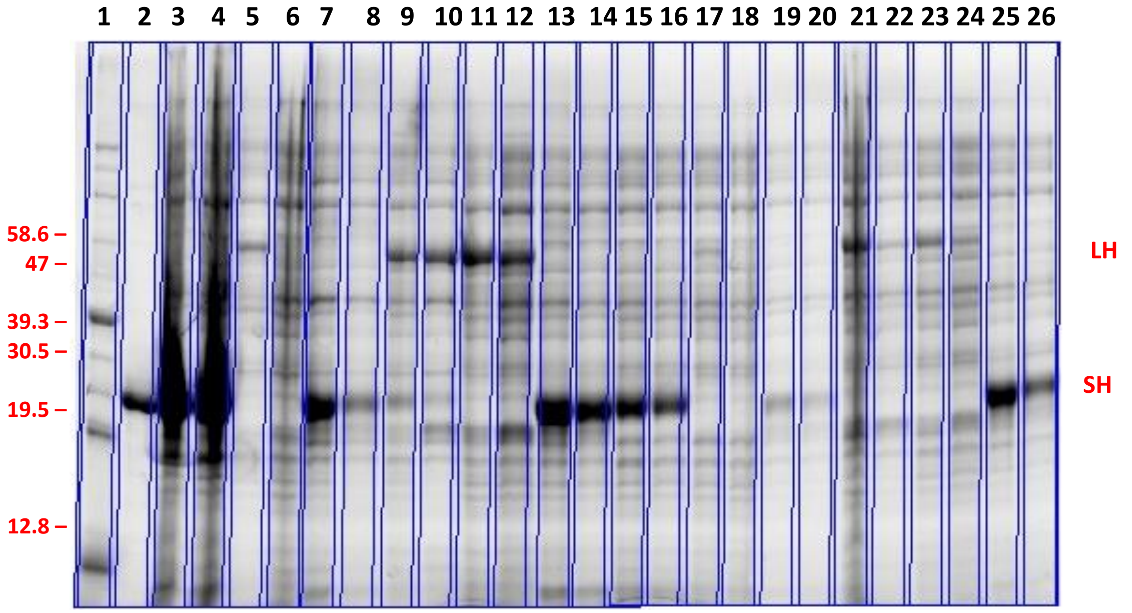

gel XX

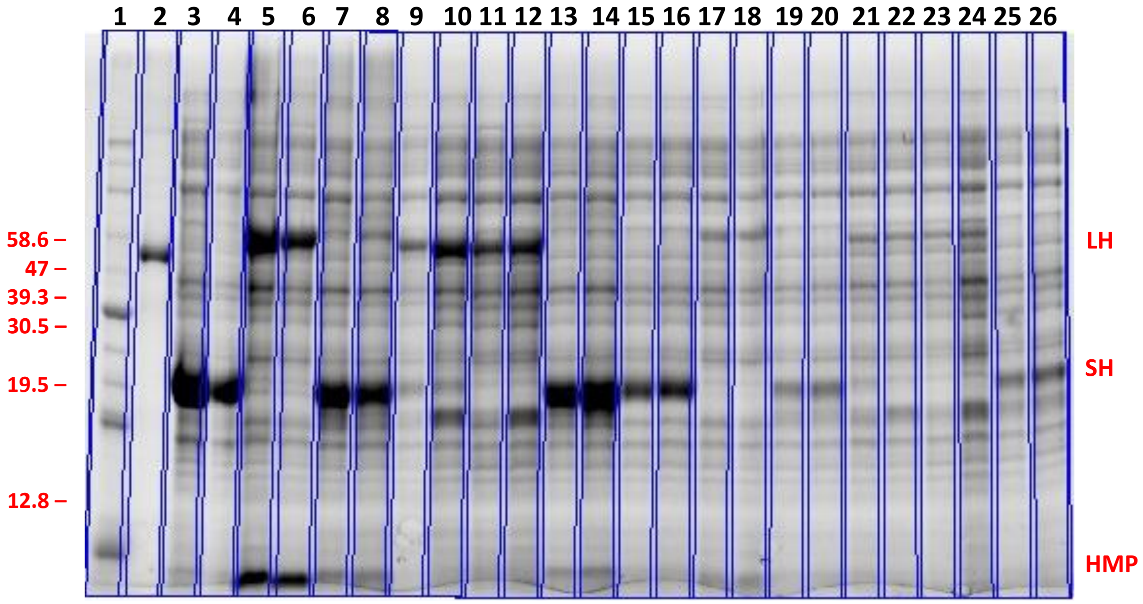

Suppl. Fig.8B. SDS-PAGE analysis of the cell lysates obtained after co-expression experiments (Suppl. Tab.8): gels XIX and XX. Large subunit (LH), small subunit (SH) and hydrogenase maturation protease (HMP) abbreviations are placed corresponding to the predicted POIs migration in gel.

1.7 Optimization of the small subunit solubility

According to the results of previous experiments (especially concerning hydrogenase complex formation via co-expression of two-piece operon elements), the SH solubility level is very low. Although a high level of the overproduced recombinant SH variants were observed in total protein samples, only faint protein bands appeared after SDS-PAGE analysis of the IMAC elution fractions. This observation was true for both cytoplasmic and periplasmic SH variants, irrespectively of whether they are grown in anaerobic or aerobic conditions. Supplementation of culture media with inorganic species (contributing to significant LH sites) showed a positive effect on the solubility and biosynthesis levels of these proteins. Thus, it was decided to employ a similar approach to improve the levels of the soluble SH subunit.

1.7.1 Effect of culture medium additives

To improve the SH solubility, an experiment focused on the expression conditions was conducted. Additives for LB broth were tested as follows (Suppl. Fig.9): M9 “Pink” Medium Metal Supplement (marked as ‘sup’), additional Fe supplementation (25 μM), pyridoxal phosphate PLP (1 mM), and cysteine (50 μM). In addition, standard 40 mM phosphate and 27.5 mM glucose were added upon induction, unless otherwise stated.

Transformation of *E. coli* with SH_v53 and SH_vDsbA constructs was performed. Transformants were grown in 3 ml of LB medium with a range of supplements at 37˚C. After 17 hours from induction with IPTG, 1.5 ml of each culture was processed and the rest was frozen for further experiments. SDS-PAGE was performed for the analysis of protein samples after IMAC purification (Suppl. Fig.9).

gel XXI

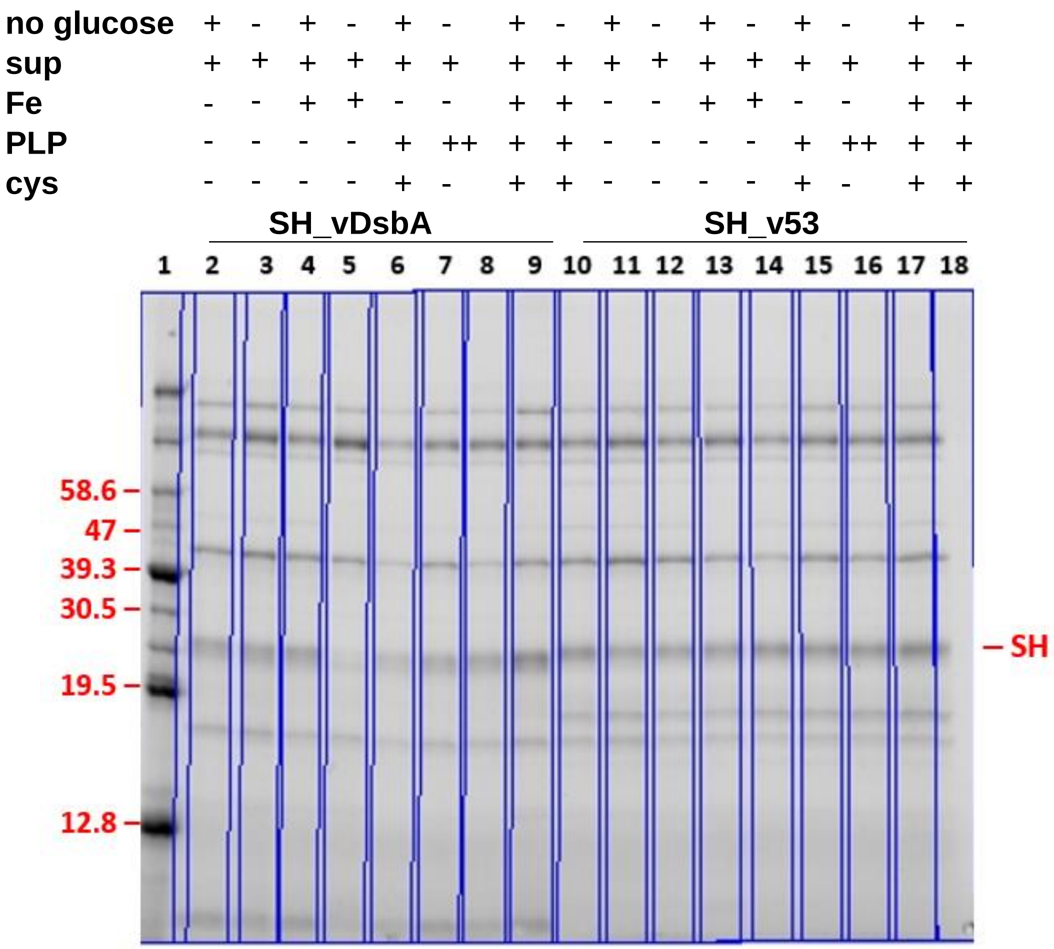

Suppl. Fig.9. Optimization of growth conditions for the improvement of the SH subunit solubility. Protein samples obtained after IMAC were analyzed by SDS-PAGE. Abbreviations: sup - M9 “Pink” Medium Metal Supplement, Fe – iron supplementation, PLP – pyridoxal phosphate supplementation, cys – cysteine supplementation. Small subunit (SH) abbreviation is placed corresponding to the predicted POIs migration in gel.

The described experiment indicated that the SH solubility was only slightly improved in the presence of the investigated additives. Therefore, solubilization and an additional step involving *in vitro* Fe-S clusters incorporation will be employed to obtain higher levels of soluble SH.

1.7.2 Small subunit solubilization and *in vitro* Fe-S clusters incorporation

Since the observed levels of expressed SH are high, but only a small amount can be detected after IMAC purification, it can indicate a solubility issue and inclusion bodies (IBs) formation. There are several different strategies known for inclusion body solubilization and refolding (Kielkopf et al., 2021; Padhiar et al., 2018; Singhvi et al., 2020). Mild chemical agents or physical methods can be used but the efficacy of solubilization depends on the IB type. Strong denaturing agents can be efficient with different types of IBs and are cost-effective. However, the use of urea or guanidine chloride can impair susceptible elements of H2ase, particularly cubane iron-sulfur clusters.

The constructs chosen for solubilization were the same as for supplementation screening: SH_v53 and SH_vDsbA. The plasmids were transformed and grown in 1L of LB media. During the procedure, samples for SDS_PAGE analysis were collected. Bacterial pellets obtained from the induced overnight cultures were subjected to disruption (soluble fraction sample), washing with 2M urea buffer (first wash sample), and solubilization in a buffer with or without TCEP (incubation samples).

It is possible to use chemical or enzymatic methods to increase the occupancy of the Fe- S clusters (Gervason et al., 2019; Külzer et al., 1998; Tsai & Tainer, 2018). Briefly, a sample containing SH subjected to solubilization was gradually mixed with $FeCl_3$ to a final concentration of 1 mM of the latter. Concentrated Na_2S solution was added dropwise - also to a concentration of 1 mM. The resulting mixture was incubated for 60 min with stirring, at RT (Fe-S sample).

The results of SDS-PAGE analysis of the collected samples are shown below (Suppl. Fig.10; Suppl. Tab.9).

Suppl. Tab.9. Attempts to increase SH solubility. Table shows samples taken from different solubilization steps and their location on the polyacrylamide gel (Suppl. Fig.10).

**sample**	**annotations**	**gel no. Suppl. Fig.10**	**lane**
protein marker ladder		XXII	1
gel number sample		XXII	2
LH C493_rbs517_HMP_v53	soluble fraction sample	XXII	3
SH_vDsbA	incubation sample, no TCEP	XXII	4
SH_vDsbA	soluble fraction sample	XXII	5
SH_v53	incubation sample, no TCEP	XXII	6
SH_v53	soluble fraction sample	XXII	7
SH_vDsbA	incubation sample, TCEP	XXII	8
LH C493_v53	soluble fraction sample	XXII	9
SH_v53	incubation sample, TCEP	XXII	10
SH_vDsbA	first wash total sample	XXII	11
SH_vDsbA	Fe-S sample, no TCEP	XXII	12
SH_v53	first wash total sample	XXII	13
SH_v53	Fe-S sample, no TCEP	XXII	14
SH_vDsbA	first wash soluble sample	XXII	15
SH_vDsbA	Fe-S sample, TCEP	XXII	16
SH_v53	first wash soluble sample	XXII	17
SH_v53	Fe-S sample, TCEP	XXII	18

gel XXII

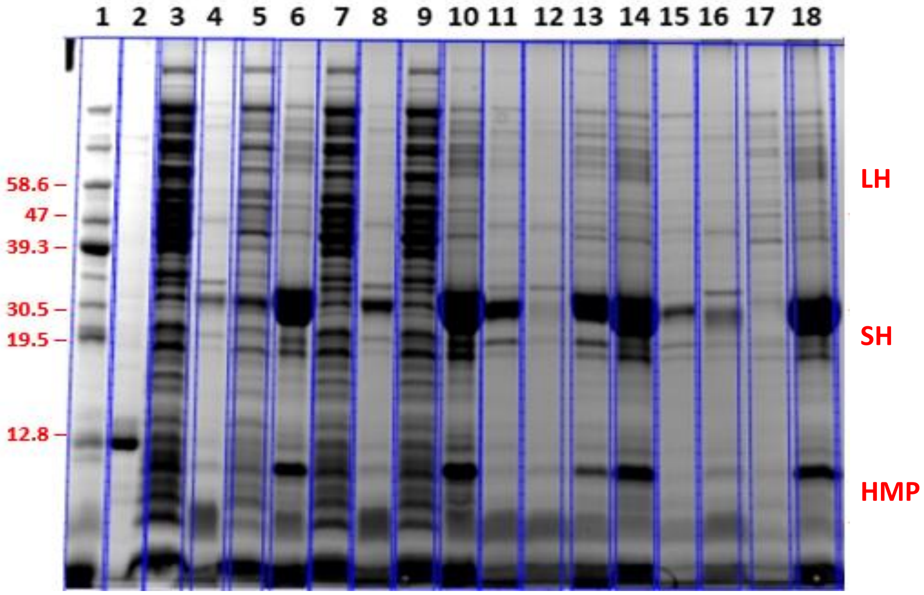

Suppl. Fig.10. SDS-PAGE analysis of the samples taken during SH solubilization experiments. Large subunit (LH), small subunit (SH) and hydrogenase maturation protease (HMP) abbreviations are placed corresponding to the predicted POIs migration in gel.

1.8 Hydrogenase assembly in optimized conditions

The subunits obtained from the expression of different layouts were prepared in anaerobic conditions in supplemented LB medium [1% tryptone, 0.5% yeast extract, 1% NaCl, 1 mM $MgCl_2$, 25 µM $FeCl_3$, 50 µM $NiSO_4$] and their solubility level was confirmed via an analysis of POI occurrence in the IMAC elution fractions (Suppl. Tab.10), (Suppl. Fig.11).

Suppl. Tab.10. Constructs chosen for the biosynthesis of the POI in anaerobic conditions. Amount of POI in the soluble fraction and IMAC elution was controlled via SDS-PAGE (Suppl. Fig.11).

sample	annotations	gel no. Suppl. Fig. 11	lane
protein marker ladder		XXIII	1
gel number sample		XXIII	2
SH_v53	soluble fraction sample	XXIII	3
SH_v53	incubation sample, no TCEP	XXIII	4
LH C493_rbs514_HMP_v53	soluble fraction sample	XXIII	5
LH C493_rbs514_HMP_v53	incubation sample, no TCEP	XXIII	6
LH M493_v92	soluble fraction sample	XXIII	7
LH M493_v92	incubation sample, TCEP	XXIII	8
SH_v53+ LH C493_rbs514_HMP_v53	soluble fraction sample	XXIII	9
SH_v53+ LH C493_rbs514_HMP_v53	incubation sample, TCEP	XXIII	10
SH_v53+ LH M493_v92	first wash total sample	XXIII	11
SH_v53+ LH M493_v92	Fe-S sample, no TCEP	XXIII	12
protein marker ladder	first wash total sample	XXIV	1
gel number sample	Fe-S sample, no TCEP	XXIV	2
SH_v53	soluble fraction	XXIV	3
SH_v53 + LH M493_499STOP_vRSF	solubilized SH + soluble LH	XXIV	4
SH_v53	first wash	XXIV	5
SH_v53 + LH C493_499STOP_vRSF	solubilized SH + soluble LH	XXIV	6
LH C493_499STOP_vRSF	soluble fraction	XXIV	7
SH_v53 + LH M493_499STOP_vRSF	second IMAC	XXIV	8
LH M493_499STOP_vRSF	soluble fraction	XXIV	9
SH_v53 + LH C493_499STOP_vRSF	second IMAC	XXIV	10
SH_v53	IMAC sample	XXIV	11

gel XXIII

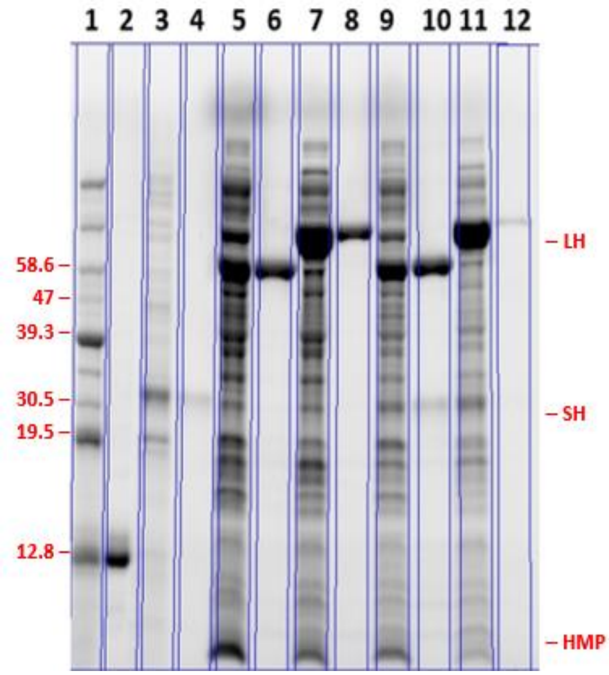

gel XXIV

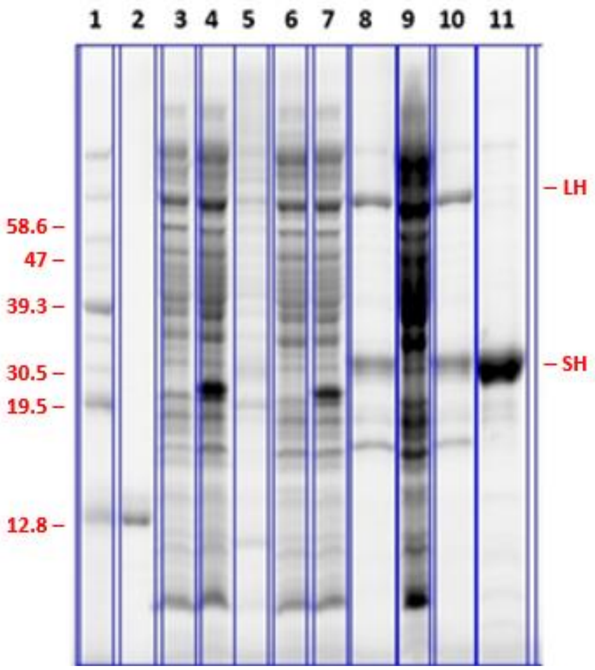

Suppl. Fig.11. SDS-PAGE analysis of the soluble POIs obtained after expressed of various DNA constructs. Large subunit (LH), small subunit (SH) and hydrogenase maturation protease (HMP) abbreviations are placed corresponding to the predicted POIs migration in gel.

1.9 Production of hydrogenase complex via expression of two-piece operon elements

Due to the truncated version of LH, (C or M)493 were tested positive for assembly with separately expressed SH (SH_v53) constructs. The operons containing both elements were constructed.

1.9.1 PCR amplification

Twelve PCR reactions were prepared with the previously obtained and verified constructs as DNA templates. The products of PCR reactions were analyzed by gel electrophoresis.

7.9.2 Annealing and transformation

PCR amplification products were purified from the excess of dNTPs and subjected to incubation with T4 DNA polymerase. DNA fragments were annealed and used for *E. coli*

transformation, resulting in 5 transformation attempts (Suppl. Tab.11). Ampicillin-resistant clones were observed after overnight incubation.

Suppl. Tab.11. Constructs chosen for expression experiments.

**vector**	**insert**	**gel no. Suppl. Fig. 12**	**lane**
v53	TEV_LH M493_499STOP	XXV	2
v53	TEV_LH C493_499STOP	XXV	3
SH_v53	rbs517_LH C493_499STOP_His-tag	XXV	4
SH_v53	rbs517_HMP_STOP_His-tag	XXV	5
SH_v53	rbs517_LH M493_499STOP_His-tag	XXV	6

1.9.3 Expression

Bacterial colonies (Suppl. Tab.11) were picked from each agar plate wells and grown in 1 ml of LB. After reaching the desired A_{600}, the recombinant gene expression was induced upon the addition of IPTG. After 18 hours from the induction, cells were spun down and lysed. Samples were analyzed using SDS-PAGE (Suppl. Fig.12).

gel XXV

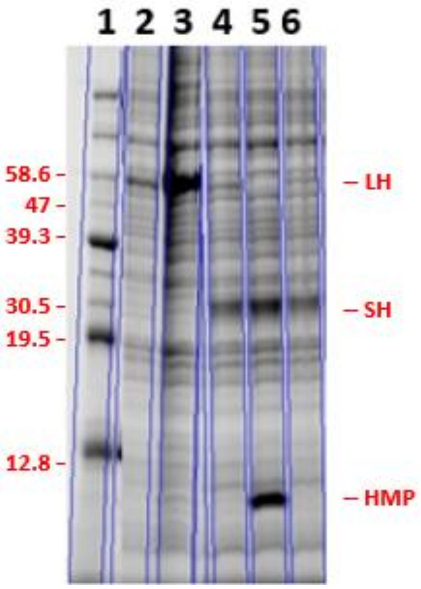

Suppl. Fig.12. SDS-PAGE analysis of the POIs biosynthesis levels obtained after expression of the selected DNA constructs, described in Suppl. Tab. 11. Large subunit (LH), small subunit (SH) and hydrogenase maturation protease (HMP) abbreviations are placed corresponding to the predicted POIs migration in gel.

1.9.4 Purification

For the activity testing, protein samples (Suppl. Tab.12) were purified by IMAC and then subjected to SEC chromatography to exclude aggregates. Fractions corresponding to the soluble, multimeric form of the enzyme were combined. Samples were analyzed using SDS-PAGE (Suppl. Fig.13).

Suppl. Tab.12.Constructs subjected to purification and their location on polyacrylamide gel.

construct	gel no. Suppl. Fig.13	lane
LH C493_499STOP_v53	XXVI	2
SH_rbs517_LH M493_499STOP_v53	XXVI	3
LH C493_499STOP_v53	XXVI	4
SH_rbs517_LH M493_499STOP_v53	XXVI	5
LH C493_499STOP_v537	XXVI	6
LH M493_499STOP_v53	XXVI	8
SH_rbs517_LH C493_499STOP_v53	XXVI	9
LH M493_499STOP_v53	XXVI	10
LH C493_STOP499_v53 + SH_v53	XXVI	11
LH C493_STOP499_v53 + SH_v53	XXVI	12
LH C493_STOP499_v53 + SH_v53	XXVI	13
LH M493_STOP499_v53 + SH_v53	XXVI	14
LH M493_STOP499_v53 + SH_v53	XXVI	15
LH M493_STOP499_v53 + SH_v53	XXVI	16
LH M493_499STOP_v53	XXVI	17
LH M493_499STOP_v53	XXVI	18

gel XXVI

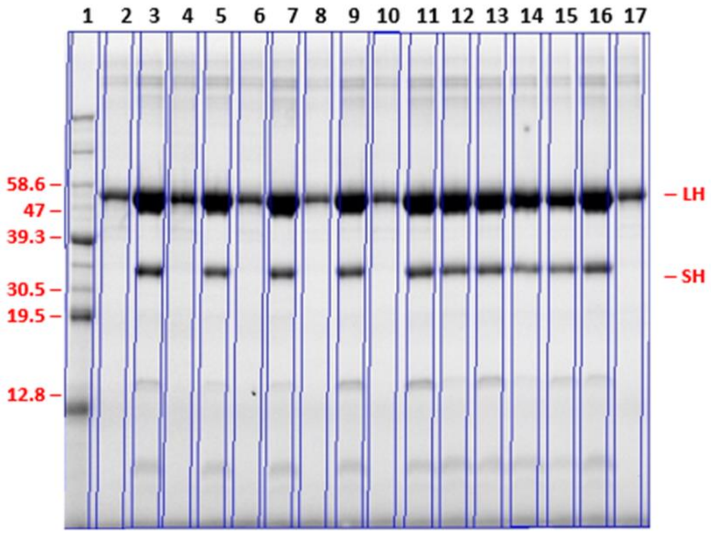

Suppl. Fig.13. SDS-PAGE analysis of the purified *Dmb* complexes, obtained by expression of the selected DNA constructs, described in Suppl. Tab. 12. Large subunit (LH), small subunit (SH) and hydrogenase maturation protease (HMP) abbreviations are placed corresponding to the predicted POIs migration in gel.

Bibliography

1. del Barrio, M., Guendon, C., Kpebe, A., Baffert, C., Fourmond, V., Brugna, M., & Léger, C. (2019). Valine-to-Cysteine Mutation Further Increases the Oxygen Tolerance of Escherichia coli NiFe Hydrogenase Hyd-1. ACS Catalysis, 9(5), 4084–4088. https://doi.org/10.1021/acscatal.9b00543

2. Gervason, S., Larkem, D., Mansour, A. B., Botzanowski, T., Müller, C. S., Pecqueur, L., Le Pavec, G., Delaunay-Moisan, A., Brun, O., Agramunt, J., Grandas, A., Fontecave, M., Schünemann, V., Cianférani, S., Sizun, C., Tolédano, M. B., & D'Autréaux, B. (2019). Physiologically relevant reconstitution of iron-sulfur cluster biosynthesis uncovers persulfide-processing functions of ferredoxin-2 and frataxin. Nature Communications, 10(1), 3566. https://doi.org/10.1038/s41467-019-11470-9

3. Kelly, C. L., Pinske, C., Murphy, B. J., Parkin, A., Armstrong, F., Palmer, T., & Sargent, F. (2015). Integration of an [FeFe]-hydrogenase into the anaerobic metabolism of Escherichia coli. Biotechnology Reports, 8, 94–104. https://doi.org/10.1016/j.btre.2015.10.002

4. Khorasani-Motlagh, M., Noroozifar, M., Kerman, K., & Zamble, D. B. (2019). Complex formation between the Escherichia coli [NiFe]-hydrogenase nickel maturation factors. BioMetals, 32(3), 521–532. https://doi.org/10.1007/s10534-019-00173-9

5. Kielkopf, C. L., Bauer, W., & Urbatsch, I. L. (2021). Solubilization of Expressed Proteins from Inclusion Bodies. Cold Spring Harbor Protocols, 2021(2), pdb.prot102210. https://doi.org/10.1101/pdb.prot102210

6. Kuchenreuther, J. M., Grady-Smith, C. S., Bingham, A. S., George, S. J., Cramer, S. P., & Swartz, J. R. (2010). High-Yield Expression of Heterologous [FeFe] Hydrogenases in Escherichia coli. PLOS ONE, 5(11), e15491. https://doi.org/10.1371/journal.pone.0015491

7. Külzer, R., Pils, T., Kappl, R., Hüttermann, J., & Knappe, J. (1998). Reconstitution and Characterization of the Polynuclear Iron-Sulfur Cluster in Pyruvate Formate-lyase-activating Enzyme: MOLECULAR PROPERTIES OF THE HOLOENZYME FORM *. Journal of Biological Chemistry, 273(9), 4897–4903. https://doi.org/10.1074/jbc.273.9.4897

8. Lacasse, M. J., & Zamble, D. B. (2016). [NiFe]-Hydrogenase Maturation. Biochemistry, 55(12), 1689–1701. https://doi.org/10.1021/acs.biochem.5b01328

9. Lukey, M. J., Roessler, M. M., Parkin, A., Evans, R. M., Davies, R. A., Lenz, O., Friedrich, B., Sargent, F., & Armstrong, F. A. (2011). Oxygen-Tolerant [NiFe]-Hydrogenases: The Individual and Collective Importance of Supernumerary Cysteines at the Proximal Fe-S Cluster. Journal of the American Chemical Society, 133(42), 16881–16892. https://doi.org/10.1021/ja205393w

10. Padhiar, A. A., Chanda, W., Joseph, T. P., Guo, X., Liu, M., Sha, L., Batool, S., Gao, Y., Zhang, W., Huang, M., & Zhong, M. (2018). Comparative study to develop a single method for retrieving wide class of recombinant proteins from classical inclusion bodies. Applied Microbiology and Biotechnology, 102(5), 2363–2377. https://doi.org/10.1007/s00253-018-8754-6

11. Pinske, C., Thomas, C., Nutschan, K., & Sawers, R. G. (2019). Delimiting the Function of the C-Terminal Extension of the Escherichia coli [NiFe]-Hydrogenase 2 Large Subunit Precursor. Frontiers in Microbiology, 10. https://doi.org/10.3389/fmicb.2019.02223

12. Sawers, R. G., Blokesch, M., & Böck, A. (2004). Anaerobic Formate and Hydrogen Metabolism. EcoSal Plus, 1(1). https://doi.org/10.1128/ecosalplus.3.5.4

13. Singhvi, P., Saneja, A., Srichandan, S., & Panda, A. K. (2020). Bacterial Inclusion Bodies: A Treasure Trove of Bioactive Proteins. Trends in Biotechnology, 38(5), 474–486. https://doi.org/10.1016/j.tibtech.2019.12.011

14. Song, Y., Liu, M., Xie, L., You, C., Sun, J., & Zhang, Y.-H. P. J. (2019). A Recombinant 12-His Tagged Pyrococcus furiosus Soluble [NiFe]-Hydrogenase I Overexpressed in Thermococcus kodakarensis KOD1 Facilitates Hydrogen-Powered in vitro NADH Regeneration. Biotechnology Journal, 14(4), 1800301. https://doi.org/10.1002/biot.201800301

15. Stripp, S. T., Lindenstrauss, U., Granich, C., Sawers, R. G., & Soboh, B. (2014). The Influence of Oxygen on [NiFe]–Hydrogenase Cofactor Biosynthesis and How Ligation of Carbon Monoxide Precedes Cyanation. PLOS ONE, 9(9), e107488. https://doi.org/10.1371/journal.pone.0107488

16. Sun, J., Hopkins, R. C., Jr, F. E. J., McTernan, P. M., & Adams, M. W. W. (2010). Heterologous Expression and Maturation of an NADP-Dependent [NiFe]-Hydrogenase: A Key Enzyme in Biofuel Production. PLOS ONE, 5(5), e10526. https://doi.org/10.1371/journal.pone.0010526

17. Theodoratou, E., Huber, R., & Böck, A. (2005). [NiFe]-Hydrogenase maturation endopeptidase: Structure and function. Biochemical Society Transactions, 33(1), 108–111. https://doi.org/10.1042/BST0330108

18. Tsai, C. L., & Tainer, J. A. (2018). Robust Production, Crystallization, Structure Determination, and Analysis of [Fe–S] Proteins: Uncovering Control of Electron Shuttling and Gating in the Respiratory Metabolism of Molybdopterin Guanine Dinucleotide Enzymes. Methods in Enzymology, 599, 157–196. https://doi.org/10.1016/bs.mie.2017.11.006

19. Vincent, K. A., Parkin, A., Lenz, O., Albracht, S. P. J., Fontecilla-Camps, J. C., Cammack, R., Friedrich, B., & Armstrong, F. A. (2005). Electrochemical Definitions of O2 Sensitivity and Oxidative Inactivation in Hydrogenases. Journal of the American Chemical Society, 127(51), 18179–18189. https://doi.org/10.1021/ja055160v
