# Supplementary material for: Promising approaches for the assembly of the catalytically active, recombinant Desulfomicrobium baculatum hydrogenase with substitutions at the active site
Source: Microb Cell Fact. 2023 Jul 21;22:134. doi: 10.1186/s12934-023-02127-w (PMC10362691; doi:10.1186/s12934-023-02127-w)
Supplement: Supplementary file 4 — Additional file 4: Procedures for the solubilization of SH constructs and for in vitro Fe-S clusters insertion. [file 12934_2023_2127_MOESM4_ESM.pdf]

Supplementary Material file 4.

(A) Procedure for the solubilization of SH constructs.
Bacterial pellets obtained from the induced overnight cultures were collected and the culture media was discarded. The pellet was suspended in lysis buffer (50 mM sodium phosphate buffer pH 8.0 at RT, 500 mM NaCl, 1 mM PMSF, 5% glycerol) with 2% Triton X-100. The samples were sonicated on ice with 10 pulses x 10 seconds and centrifuged at high speed for 30 min at 4°C. The soluble fraction sample was taken from the supernatant and the bacterial cell pellet was resuspended in solubilization buffer (lysis buffer with 2M urea, 2% Triton X-100) and further sonicated and centrifuged as described above (1st wash sample). The pellet was subjected to a second round of solubilisation with repeated washing, sonication, and centrifugation steps. Finally, the isolated inclusion bodies (pellet) were resuspended in solubilization buffer with or without 1 mM TCEP. The mixture was incubated for 60 min at RT with gentle stirring and centrifuged for 15 min at 4°C at high speed. The resulting supernatant contained solubilized POIs. The excess of urea was gradually removed from the protein solution by dialysis, which allowed the protein to refold. Final centrifugation was performed to remove the remaining insoluble particles.

(B) Procedure for in vitro Fe-S clusters insertion.
A sample containing solubilized SH (obtained as a result of solubilization procedure described above) was gradually mixed with $FeCl_3$ to a final concentration of 1 mM of the latter. Na_2S was added dropwise to a final concentration of 1 mM. The resulting mixture was incubated for 60 min, with stirring, at RT (Fe-S sample). For further analyses, reconstitution mixtures were gel filtered through Sephadex G-25 columns.

(C) Results

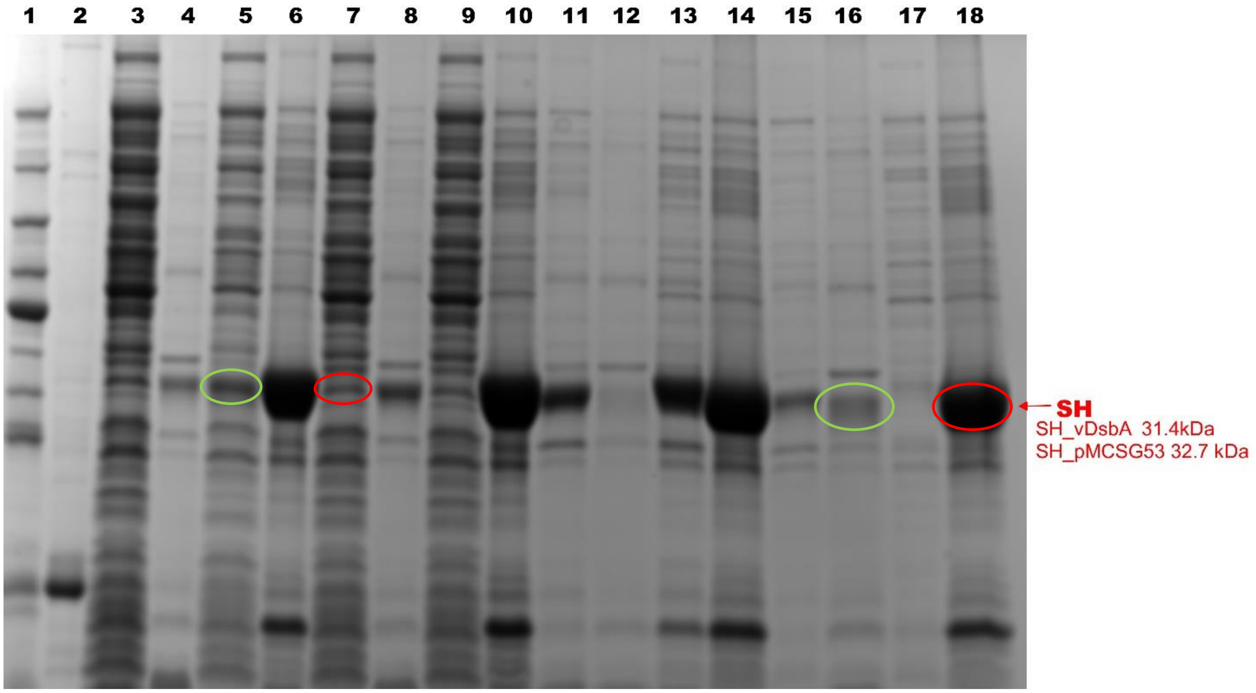

Fig.1. Assessing soluble and insoluble protein expression levels during solubilization and Fe-S cluster insertion. For the experiment, two constructs with SH were selected – with the DsbA signal sequence (SH_vDsbA) and without it (SH_v53). SDS-PAGE of total and soluble samples taken at the different stages described in section A and B. Soluble fraction samples (lanes 5 and 7) and sample after the final step of Fe-S cluster insertion (lane 16 and 18) are marked with red (SH_v53) and green (SH_vDsbA) circles. All lanes are described in the table below (Table 1).

Table 1. Assessing soluble and insoluble protein expression levels during solubilization and Fe-S cluster insertion. Lanes in Figure 1 and sample descriptions according to procedures in sections A and B are presented.

sample	annotations	lane
protein marker ladder		1
gel number sample		2
LH C493_rbs517_HMP_v53	soluble fraction sample	3
SH_vDsbA	incubation sample	4
SH_vDsbA	soluble fraction sample	5
SH_v53	incubation sample	6
SH_v53	soluble fraction sample	7
SH_vDsbA	incubation sample, TCEP	8
LH C493_v53	soluble fraction sample	9
SH_v53	incubation sample, TCEP	10
SH_vDsbA	1st wash total sample	11
SH_vDsbA	Fe-S sample	12
SH_v53	1st wash total sample	13
SH_v53	Fe-S sample	14
SH_vDsbA	1st wash soluble sample	15
SH_vDsbA	Fe-S sample, TCEP	16
SH_v53	1st wash soluble sample	17
SH_v53	Fe-S sample, TCEP	18
