# Supplementary material for: Promising approaches for the assembly of the catalytically active, recombinant Desulfomicrobium baculatum hydrogenase with substitutions at the active site
Source: Microb Cell Fact. 2023 Jul 21;22:134. doi: 10.1186/s12934-023-02127-w (PMC10362691; doi:10.1186/s12934-023-02127-w)
Supplement: Supplementary file 5 — Additional file 5: Genetic map and DNA sequence of the Dmb hydrogenase SH_LH_U493C_pMCSG53 recombinant construct. [file 12934_2023_2127_MOESM5_ESM.pdf]

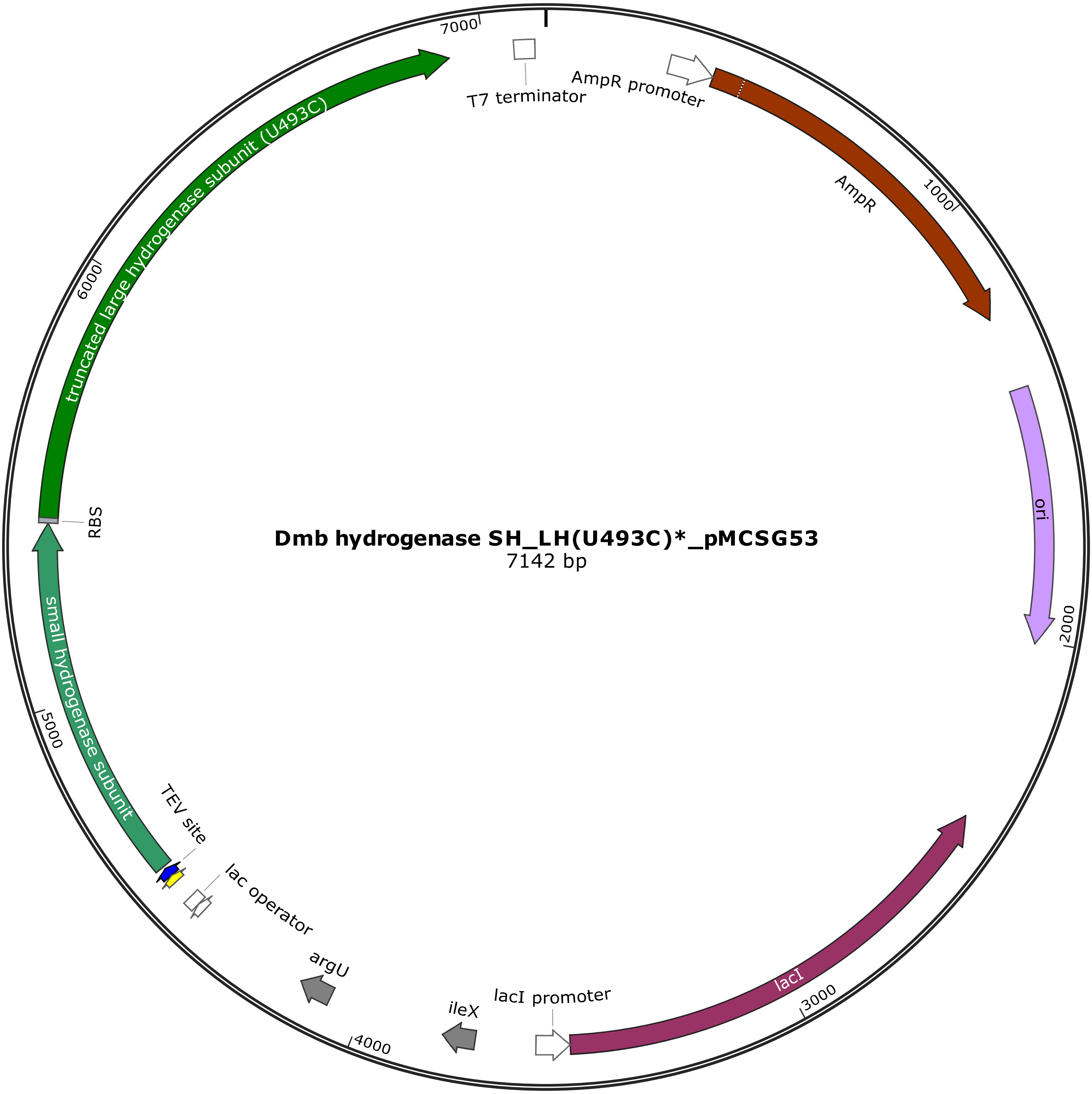
Dmb hydrogenase SH_LH(U493C)*_pMCSG53
7142 bp
T7 terminator
AmpR promoter
AmpR
1000
ori
2000
lacI
3000
lacI promoter
ileX
4000
argU
lac operator
TEV site
small hydrogenase subunit
5000
RBS
truncated large hydrogenase subunit (U493C)
6000
7000

```
5' TGGCGAATGGGACGCGCCCTGTAGCGGCGCATTAAGCGCGGCGGGTGTGGTGGTTACGCG   60
3' ACCGCTTACCCTGCGCGGGACATCGCCGCGTAATTCGCGCCGCCCACACCACCAATGCGC

   CAGCGTGACCGCTACACTTGCCAGCGCCCTAGCGCCCGCTCCTTTCGCTTTCTTCCCTTC   120
   GTCGCACTGGCGATGTGAACGGTCGCGGGATCGCGGGCGAGGAAAGCGAAAGAAGGGAAG

   CTTTCTCGCCACGTTCGCCGGCTTTCCCCGTCAAGCTCTAAATCGGGGGCTCCCTTTAGG   180
   GAAAGAGCGGTGCAAGCGGCCGAAAGGGGCAGTTCGAGATTTAGCCCCCGAGGGAAATCC

   GTTCCGATTTAGTGCTTTACGGCACCTCGACCCCAAAAAACTTGATTAGGGTGATGGTTC   240
   CAAGGCTAAATCACGAAATGCCGTGGAGCTGGGGTTTTTTGAACTAATCCCACTACCAAG

   ACATTAACGCTTACAATTTAGGTGGCACTTTTCGGGGAAATGTGCGCGGAACCCCTATTT   300
   TGTAATTGCGAATGTTAAATCCACCGTGAAAAGCCCCTTTACACGCGCCTTGGGGATAAA
                                                 AmpR promoter >

   GTTTATTTTTCTAAATACATTCAAATATGTATCCGCTCATGAGACAATAACCCTGATAAA   360
   CAAATAAAAAGATTTATGTAAGTTTATACATAGGCGAGTACTCTGTTATTGGGACTATTT
                             AmpR promoter >

   TGCTTCAATAATTTGAAAAAGGAAGAGTATGAGTATTCAACATTTCCGTGTCGCCCTTAT   420
   ACGAAGTTATTAAACTTTTTCCTTCTCATACTCATAAGTTGTAAAGGCACAGCGGGAATA
                                1           5              10
                                M  S  I  Q  H  F  R  V  A  L  I
          AmpR promoter >             signal sequence >
                                           AmpR

   TCCCTTTTTTGCGGCATTTTGCCTTCCTGTTTTTGCTCACCCAGAAACGCTGGTGAAAGT   480
   AGGGAAAAAACGCCGTAAAACGGAAGGACAAAAACGAGTGGGTCTTTGCGACCACTTTCA
            15             20             25             30
    P  F  F  A  A  F  C  L  P  V  F  A  H  P  E  T  L  V  K  V
        signal sequence                                       >
                             AmpR

   AAAAGATGCTGAAGATCAGTTGGGTGCACGAGTGGGTTACATCGAACTGGATCTCAACAG   540
   TTTTCTACGACTTCTAGTCAACCCACGTGCTCACCCAATGTAGCTTGACCTAGAGTTGTC
            35             40             45             50
    K  D  A  E  D  Q  L  G  A  R  V  G  Y  I  E  L  D  L  N  S
                             AmpR                             >

   CGGTAAGATCCTTGAGAGTTTTCGCCCCGAAGAACGTTTTCCAATGATGAGCACTTTTAA   600
   GCCATTCTAGGAACTCTCAAAAGCGGGGCTTCTTGCAAAAGGTTACTACTCGTGAAAATT
            55             60             65             70
    G  K  I  L  E  S  F  R  P  E  E  R  F  P  M  M  S  T  F  K
                             AmpR                             >
```

AGTTCTGCTATGTGGCGCGGTATTATCCCGTATTGATGCCGGGCAAGAGCAACTCGGTCG 660
TCAAGACGATACACCGCGCCATAATAGGGCATAACTACGGCCCGTTCTCGTTGAGCCAGC

75 80 85 90
V L L C G A V L S R I D A G Q E Q L G R

AmpR

CCGCATACACTATTCTCAGAATGACTTGGTTGAGTACTCACCAGTCACAGAAAAGCATCT 720
GGCGTATGTGATAAGAGTCTTACTGAACCAACTCATGAGTGGTCAGTGTCTTTTCGTAGA

95 100 105 110
R I H Y S Q N D L V E Y S P V T E K H L

AmpR

TACGGATGGCATGACAGTAAGAGAATTATGCAGTGCTGCCATAACCATGAGTGATAACAC 780
ATGCCTACCGTACTGTCATTCTCTTAATACGTCACGACGGTATTGGTACTCACTATTGTG

115 120 125 130
T D G M T V R E L C S A A I T M S D N T

AmpR

TGCGGCCAACTTACTTCTGACAACGATCGGAGGACCGAAGGAGCTAACCGCTTTTTTGCA 840
ACGCCGGTTGAATGAAGACTGTTGCTAGCCTCCTGGCTTCCTCGATTGGCGAAAAAACGT

135 140 145 150
A A N L L L T T I G G P K E L T A F L H

AmpR

CAACATGGGGGATCATGTAACTCGCCTTGATCGTTGGGAACCGGAGCTGAATGAAGCCAT 900
GTTGTACCCCCTAGTACATTGAGCGGAACTAGCAACCCTTGGCCTCGACTTACTTCGGTA

155 160 165 170
N M G D H V T R L D R W E P E L N E A I

AmpR

ACCAAACGACGAGCGTGACACCACGATGCCTGCAGCAATGGCAACAACGTTGCGCAAACT 960
TGGTTTGCTGCTCGCACTGTGGTGCTACGGACGTCGTTACCGTTGTTGCAACGCGTTTGA

175 180 185 190
P N D E R D T T M P A A M A T T L R K L

AmpR

ATTAACTGGCGAACTACTTACTCTAGCTTCCCGGCAACAATTAATAGACTGGATGGAGGC 1020
TAATTGACCGCTTGATGAATGAGATCGAAGGGCCGTTGTTAATTATCTGACCTACCTCCG

195 200 205 210
L T G E L L T L A S R Q Q L I D W M E A

AmpR

GGATAAAGTTGCAGGACCACTTCTGCGCTCGGCCCTTCCGGCTGGCTGGTTTATTGCTGA 1080
CCTATTTCAACGTCCTGGTGAAGACGCGAGCCGGGAAGGCCGACCGACCAAATAACGACT

215 220 225 230
D K V A G P L L R S A L P A G W F I A D

AmpR

TAAATCTGGAGCCGGTGAGCGTGGGTCTCGCGGTATCATTGCAGCACTGGGGCCAGATGG 1140
ATTTAGACCTCGGCCACTCGCACCCAGAGCGCCATAGTAACGTCGTGACCCCGGTCTACC

235 240 245 250
K S G A G E R G S R G I I A A L G P D G
AmpR

TAAGCCCTCCCGTATCGTAGTTATCTACACGACGGGGAGTCAGGCAACTATGGATGAACG 1200
ATTCGGGAGGGCATAGCATCAATAGATGTGCTGCCCCTCAGTCCGTTGATACCTACTTGC

255 260 265 270
K P S R I V V I Y T T G S Q A T M D E R
AmpR

AAATAGACAGATCGCTGAGATAGGTGCCTCACTGATTAAGCATTGGTAACTGTCAGACCA 1260
TTTATCTGTCTAGCGACTCTATCCACGGAGTGACTAATTCGTAACCATTGACAGTCTGGT

275 280 285
N R Q I A E I G A S L I K H W *
AmpR

AGTTTACTCATATATACTTTAGATTGATTTAAAACTTCATTTTTAATTTAAAAGGATCTA 1320
TCAAATGAGTATATATGAAATCTAACTAAATTTTGAAGTAAAAATTAAATTTTCCTAGAT

GGTGAAGATCCTTTTTGATAATCTCATGACCAAAATCCCTTAACGTGAGTTTTCGTTCCA 1380
CCACTTCTAGGAAAAACTATTAGAGTACTGGTTTTAGGGAATTGCACTCAAAAGCAAGGT

CTGAGCGTCAGACCCCGTAGAAAAGATCAAAGGATCTTCTTGAGATCCTTTTTTTCTGCG 1440
GACTCGCAGTCTGGGGCATCTTTTCTAGTTTCCTAGAAGAACTCTAGGAAAAAAAGACGC
ori

CGTAATCTGCTGCTTGCAAACAAAAAAACCACCGCTACCAGCGGTGGTTTGTTTGCCGGA 1500
GCATTAGACGACGAACGTTTGTTTTTTTGGTGGCGATGGTCGCCACCAAACAAACGGCCT
ori

TCAAGAGCTACCAACTCTTTTTCCGAAGGTAACTGGCTTCAGCAGAGCGCAGATACCAAA 1560
AGTTCTCGATGGTTGAGAAAAAGGCTTCCATTGACCGAAGTCGTCTCGCGTCTATGGTTT
ori

TACTGTCCTTCTAGTGTAGCCGTAGTTAGGCCACCACTTCAAGAACTCTGTAGCACCGCC 1620
ATGACAGGAAGATCACATCGGCATCAATCCGGTGGTGAAGTTCTTGAGACATCGTGGCGG
ori

TACATACCTCGCTCTGCTAATCCTGTTACCAGTGGCTGCTGCCAGTGGCGATAAGTCGTG 1680
ATGTATGGAGCGAGACGATTAGGACAATGGTCACCGACGACGGTCACCGCTATTCAGCAC
ori

TCTTACCGGGTTGGACTCAAGACGATAGTTACCGGATAAGGCGCAGCGGTCGGGCTGAAC 1740
AGAATGGCCCAACCTGAGTTCTGCTATCAATGGCCTATTCCGCGTCGCCAGCCCGACTTG
ori

GGGGGGTTCGTGCACACAGCCCAGCTTGGAGCGAACGACCTACACCGAACTGAGATACCT 1800
CCCCCCAAGCACGTGTGTCGGGTCGAACCTCGCTTGCTGGATGTGGCTTGACTCTATGGA
ori

ACAGCGTGAGCTATGAGAAAGCGCCACGCTTCCCGAAGGGAGAAAGGCGGACAGGTATCC 1860
TGTCGCACTCGATACTCTTTCGCGGTGCGAAGGGCTTCCCTCTTTCCGCCTGTCCATAGG
ori

GGTAAGCGGCAGGGTCGGAACAGGAGAGCGCACGAGGGAGCTTCCAGGGGGAAACGCCTG 1920
CCATTCGCCGTCCCAGCCTTGTCCTCTCGCGTGCTCCCTCGAAGGTCCCCCTTTGCGGAC
ori

GTATCTTTATAGTCCTGTCGGGTTTCGCCACCTCTGACTTGAGCGTCGATTTTTGTGATG 1980
CATAGAAATATCAGGACAGCCCAAAGCGGTGGAGACTGAACTCGCAGCTAAAAACACTAC
ori

CTCGTCAGGGGGGCGGAGCCTATGGAAAAACGCCAGCAACGCGGCCTTTTTACGGTTCCT 2040
GAGCAGTCCCCCCGCCTCGGATACCTTTTTGCGGTCGTTGCGCCGGAAAAATGCCAAGGA
ori

GGCCTTTTGCTGGCCTTTTGCTCACATGTTCTTTCCTGCGTTATCCCCTGATTCTGTGGA 2100
CCGGAAAACGACCGGAAAACGAGTGTACAAGAAAGGACGCAATAGGGGACTAAGACACCT

TAACCGTATTACCGCCTTTGAGTGAGCTGATACCGCTCGCCGCAGCCGAACGACCGAGCG 2160
ATTGGCATAATGGCGGAAACTCACTCGACTATGGCGAGCGGCGTCGGCTTGCTGGCTCGC

CAGCGAGTCAGTGAGCGAGGAAGCGGAAGAGCGCCTGATGCGGTATTTTCTCCTTACGCA 2220
GTCGCTCAGTCACTCGCTCCTTCGCCTTCTCGCGGACTACGCCATAAAAGAGGAATGCGT

TCTGTGCGGTATTTCACACCGCAATGGTGCACTCTCAGTACAATCTGCTCTGATGCCGCA 2280
AGACACGCCATAAAGTGTGGCGTTACCACGTGAGAGTCATGTTAGACGAGACTACGGCGT

TAGTTAAGCCAGTATACACTCCGCTATCGCTACGTGACTGTAGTCATGCCCCGCGCCCAC 2340
ATCAATTCGGTCATATGTGAGGCGATAGCGATGCACTGACATCAGTACGGGGCGCGGGTG

CGGAGGGAGCTGACTGGGTTGAAGGCTCTCAAGGGCATCGGTCGAGATCCCGGTGCCTAA 2400
GCCTCCCTCGACTGACCCAACTTCCGAGAGTTCCCGTAGCCAGCTCTAGGGCCACGGATT

```
TGAGTGAGCTAACTTACATTAATTGCGTTGCGTTCACTGCCCGCTTTCCAGTCGGGAAAC   2460
ACTCACTCGATTGAATGTAATTAACGCAACGCAAGTGACGGGCGAAAGGTCAGCCCTTTG
                                                360       355
                                                *  Q  G  S  E  L  R  S  V
                                                lacI

CTGTCGTGCCAGCTGCATTAATGAATCGGCCAACGCGCGGGGAGAGGCGGTTTGCGTATT   2520
GACAGCACGGTCGACGTAATTACTTAGCCGGTTGCGCGCCCCTCTCCGCCAAACGCATAA
      350            345            340            335
 Q  R  A  L  Q  M  L  S  D  A  L  A  R  P  S  A  T  Q  T  N
lacI

GGGCGCCAGGGTGGTTTTTCTTTTCACCAGTGAGACGGGCAACAGCTGATTGCCCTTCAC   2580
CCCGCGGTCCCACCAAAAAGAAAAGTGGTCACTCTGCCCGTTGTCGACTAACGGGAAGTG
      330            325            320            315
 P  A  L  T  T  K  R  K  V  L  S  V  P  L  L  Q  N  G  K  V
lacI

CGCCTGGCCCTGAGAGAGTTGCAGCAAGCGGTCCACGCTGGTTTGCCCCAGCAGGCGAAA   2640
GCGGACCGGGACTCTCTCAACGTCGTTCGCCAGGTGCGACCAAACGGGGTCGTCCGCTTT
      310            305            300            295
 A  Q  G  Q  S  L  Q  L  L  R  D  V  S  T  Q  G  L  L  R  F
lacI

ATCCTGTTTGATGGTGGTTAACGGCGGGATATAACATGAGCTGTCTTCGGTATCGTCGTA   2700
TAGGACAAACTACCACCAATTGCCGCCCTATATTGTACTCGACAGAAGCCATAGCAGCAT
      290            285            280            275
 D  Q  K  I  T  T  L  P  P  I  Y  C  S  S  D  E  T  D  D  Y
lacI

TCCCACTACCGAGATATCCGCACCAACGCGCAGCCCGGACTCGGTAATGGCGCGCATTGC   2760
AGGGTGATGGCTCTATAGGCGTGGTTGCGCGTCGGGCCTGAGCCATTACCGCGCGTAACG
      270            265            260            255
 G  V  V  S  I  D  A  G  V  R  L  G  S  E  T  I  A  R  M  A
lacI

GCCCAGCGCCATCTGATCGTTGGCAACCAGCATCGCAGTGGGAACGATGCCCTCATTCAG   2820
CGGGTCGCGGTAGACTAGCAACCGTTGGTCGTAGCGTCACCCTTGCTACGGGAGTAAGTC
      250            245            240            235
 G  L  A  M  Q  D  N  A  V  L  M  A  T  P  V  I  G  E  N  L
lacI

CATTTGCATGGTTTGTTGAAAACCGGACATGGCACTCCAGTCGCCTTCCCGTTCCGCTAT   2880
GTAAACGTACCAAACAACTTTTGGCCTGTACCGTGAGGTCAGCGGAAGGGCAAGGCGATA
      230            225            220            215
 M  Q  M  T  Q  Q  F  G  S  M  A  S  W  D  G  E  R  E  A  I
lacI
```

CGGCTGAATTTGATTGCGAGTGAGATATTTATGCCAGCCAGCCAGACGCAGACGCGCCGA 2940
GCCGACTTAAACTAACGCTCACTCTATAAATACGGTCGGTCGGTCTGCGTCTGCGCGGCT
210 205 200 195
P Q I Q N R T L Y K H W G A L R L R A S
lacI

GACAGAACTTAATGGGCCCGCTAACAGCGCGATTTGCTGGTGACCCAATGCGACCAGATG 3000
CTGTCTTGAATTACCCGGGCGATTGTCGCGCTAAACGACCACTGGGTTACGCTGGTCTAC
190 185 180 175
V S S L P G A L L A I Q Q H G L A V L H
lacI

CTCCACGCCCAGTCGCGTACCGTCTTCATGGGAGAAAATAATACTGTTGATGGGTGTCTG 3060
GAGGTGCGGGTCAGCGCATGGCAGAAGTACCCTCTTTTATTATGACAACTACCCACAGAC
170 165 160 155
E V G L R T G D E H S F I I S N I P T Q
lacI

GTCAGAGACATCAAGAAATAACGCCGGAACATTAGTGCAGGCAGCTTCCACAGCAATGGC 3120
CAGTCTCTGTAGTTCTTTATTGCGGCCTTGTAATCACGTCCGTCGAAGGTGTCGTTACCG
150 145 140 135
D S V D L F L A P V N T C A A E V A I A
lacI

ATCCTGGTCATCCAGCGGATAGTTAATGATCAGCCCACTGACGCGTTGCGCGAGAAGATT 3180
TAGGACCAGTAGGTCGCCTATCAATTACTAGTCGGGTGACTGCGCAACGCGCTCTTCTAA
130 125 120 115
D Q D D L P Y N I I L G S V R Q A L L N
lacI

GTGCACCGCCGCTTTACAGGCTTCGACGCCGCTTCGTTCTACCATCGACACCACCACGCT 3240
CACGTGGCGGCGAAATGTCCGAAGCTGCGGCGAAGCAAGATGGTAGCTGTGGTGGTGCGA
110 105 100 95
H V A A K C A E V G S R E V M S V V V S
lacI

GGCACCCAGTTGATCGGCGCGAGATTTAATCGCCGCGACAATTTGCGACGGCGCGTGCAG 3300
CCGTGGGTCAACTAGCCGCGCTCTAAATTAGCGGCGCTGTTAAACGCTGCCGCGCACGTC
90 85 80 75
A G L Q D A R S K I A A V I Q S P A H L
lacI

GGCCAGACTGGAGGTGGCAACGCCAATCAGCAACGACTGTTTGCCCGCCAGTTGTTGTGC 3360
CCGGTCTGACCTCCACCGTTGCGGTTAGTCGTTGCTGACAAACGGGCGGTCAACAACACG
70 65 60 55
A L S S T A V G I L L S Q K G A L Q Q A
lacI

CACGCGGTTGGGAATGTAATTCAGCTCCGCCATCGCCGCTTCCACTTTTTCCCGCGTTTT 3420
GTGCGCCAACCCTTACATTAAGTCGAGGCGGTAGCGGCGAAGGTGAAAAAGGGCGCAAAA

50 45 40 35
V R N P I Y N L E A M A A E V K E R T K
lacI

CGCAGAAACGTGGCTGGCCTGGTTCACCACGCGGGAAACGGTCTGATAAGAGACACCGGC 3480
GCGTCTTTGCACCGACCGGACCAAGTGGTGCGCCCTTTGCCAGACTATTCTCTGTGGCCG

30 25 20 15
A S V H S A Q N V V R S V T Q Y S V G A
lacI

ATACTCTGCGACATCGTATAACGTTACTGGTTTCACATTCACCACCCTGAATTGACTCTC 3540
TATGAGACGCTGTAGCATATTGCAATGACCAAAGTGTAAGTGGTGGGACTTAACTGAGAG

10 5 1
Y E A V D Y L T V P K M
lacI
lacI promoter

TTCCGGGCGCTATCATGCCATACCGCGAAAGGTTTTGCGCCATTCGATGGTGTCCGGGAT 3600
AAGGCCCGCGATAGTACGGTATGGCGCTTTCCAAAACGCGGTAAGCTACCACAGGCCCTA

lacI promoter

CTCGACGCTCTCCCTTATGCGACTCCTGCATTAGGAAGCAGCCCAGTAGTAGGTTGAGGC 3660
GAGCTGCGAGAGGGAATACGCTGAGGACGTAATCCTTCGTCGGGTCATCATCCAACTCCG

CGTTGAGCACCGCCGCCGCAAGGAATGGTGCATGCGGATTGCGACACGGAGTTACTTTAT 3720
GCAACTCGTGGCGGCGGCGTTCCTTACCACGTACGCCTAACGCTGTGCCTCAATGAAATA

AATCCGCTACCATGGCCCCTTAGCTCAGTGGTTAGAGCAGGCGACTCATAATCGCTTGGT 3780
TTAGGCGATGGTACCGGGGAATCGAGTCACCAATCTCGTCCGCTGAGTATTAGCGAACCA

ileX

CGCTGGTTCAAGTCCAGCAGGGGCCACCAGATATAGCAAAGGCTGACGAGAAATCGTCAG 3840
GCGACCAAGTTCAGGTCGTCCCCGGTGGTCTATATCGTTTCCGACTGCTCTTTAGCAGTC

ileX

CCTTTTTGCATGCAAGGAGATGGCGCCCAACAGTCCCCCGGCCACGGGGCCTGCCACCAT 3900
GGAAAAACGTACGTTCCTCTACCGCGGGTTGTCAGGGGGCCGGTGCCCCGGACGGTGGTA

ACCCACGCCGAAACAAGCGCTCATGAGCCCGAAGTGGCGAGCCCGATCTTCCCCATCGGT 3960
TGGGTGCGGCTTTGTTCGCGAGTACTCGGGCTTCACCGCTCGGGCTAGAAGGGGTAGCCA

GATGTCGGCGATATAGGCGCCAGCAACCGCACCTGTGGCGCCGGCGAGATCAAAAGCCAT 4020
CTACAGCCGCTATATCCGCGGTCGTTGGCGTGGACACCGCGGCCGCTCTAGTTTTCGGTA

TGACTCAGCAAGGGTTGACCGTATAATTCACGCGATTACACCGCATTGCGGTATCAACGC 4080
ACTGAGTCGTTCCCAACTGGCATATTAAGTGCGCTAATGTGGCGTAACGCCATAGTTGCG
argU

GCCCTTAGCTCAGTTGGATAGAGCAACGACCTTCTAAGTCGTGGGCCGCAGGTTCGAATC 4140
CGGGAATCGAGTCAACCTATCTCGTTGCTGGAAGATTCAGCACCCGGCGTCCAAGCTTAG
argU

CTGCAGGGCGCGCCATTACAATTCAATCAGTTACGCCTTCTTTATATCCTCCATAATTCC 4200
GACGTCCCGCGCGGTAATGTTAAGTTAGTCAATGCGGAAGAAATATAGGAGGTATTAAGG
argU

AGAGTGGGACATATTTGGGACATTATCACCAAAAATGTCGTCTATTTTCCTCGCATGCTC 4260
TCTCACCCTGTATAAACCCTGTAATAGTGGTTTTTACAGCAGATAAAAGGAGCGTACGAG

TGTCAAATGATTAGGCGCAAGGTGAGCATACCTACGAACCATTTCTATGGACTCCCATCC 4320
ACAGTTTACTAATCCGCGTTCCACTCGTATGGATGCTTGGTAAAGATACCTGAGGGTAGG

GCCCATTTCCTGAAGCACTGATAATGGGACGCCTGACTGAATCCACCGGTGATGCCGGCC 4380
CGGGTAAAGGACTTCGTGACTATTACCCTGCGGACTGACTTAGGTGGCCACTACGGCCGG

ACGATGCGTCCGGCGTAGAGGATCGAGATCGATCTCGATCCCGCGAAATTAATACGACTC 4440
TGCTACGCAGGCCGCATCTCCTAGCTCTAGCTAGAGCTAGGGCGCTTTAATTATGCTGAG
T7 promoter

ACTATAGGGGAATTGTGAGCGGATAACAATTCCCCTCTAGAAATAATTTTGTTTAACTTT 4500
TGATATCCCCTTAACACTCGCCTATTGTTAAGGGGAGATCTTTATTAAAACAAATTGAAA
T7 promoter
lac operator

AAGAAGGAGATATACATATGCACCATCATCATCATCATGAGAACCTGTACTTCCAATCCA 4560
TTCTTCCTCTATATGTATACGTGGTAGTAGTAGTAGTACTCTTGGACATGAAGGTTAGGT
1 5 1 5
H H H H H H E N L Y F Q S
6xHis
TEV site

ATgccaccgagggtgcTaagaaagcAccagtgatttgggtgcagggTcagggTtgcactg 4620
TAcggtggctcccacgAttctttcgTggtcactaaacccacgtcccAgtcccAacgtgac
N A T E G A K K A P V I W V Q G Q G C T
(in frame with TEV site)
small hydrogenase subunit

```
gttgttccgtttcactgctgaatgcggtacatccgcgtattaaagaaatcctgctggacg    4680
caacaaggcaaagtgacgacttacgccatgtaggcgcataatttctttaggacgacctgc
G  C  S  V  S  L  L  N  A  V  H  P  R  I  K  E  I  L  L  D
(in frame with TEV site)
small hydrogenase subunit

tgatttcactggaatttcatccgactgttatggctagtgaaggagaaatggcactggcgc    4740
actaaagtgaccttaaagtaggctgacaataccgatcacttcctctttaccgtgaccgcg
V  I  S  L  E  F  H  P  T  V  M  A  S  E  G  E  M  A  L  A
(in frame with TEV site)
small hydrogenase subunit

acatgtatgaaattgcggaaaagttcaacggcaacttctttttgttagtggagggcgcca    4800
tgtacatactttaacgccttttcaagttgccgttgaagaaaaacaatcacctcccgcggt
H  M  Y  E  I  A  E  K  F  N  G  N  F  F  L  L  V  E  G  A
(in frame with TEV site)
small hydrogenase subunit

tcccAacTgctaaggaaggacgctactgtgtTgtAggTgagacactggacgcgaagggTc    4860
agggTtgAcgattccttcctgcgatgacacaAcaTccActctgtgacctgcgcttcccAg
I  P  T  A  K  E  G  R  Y  C  V  V  G  E  T  L  D  A  K  G
(in frame with TEV site)
small hydrogenase subunit

accaccatgaaattacgatgatggagttaattcgcgaccttgcccccaagagtcttgcaa    4920
tggtggtactttaatgctactacctcaattaagcgctggaacgggggttctcagaacgtt
H  H  H  E  I  T  M  M  E  L  I  R  D  L  A  P  K  S  L  A
(in frame with TEV site)
small hydrogenase subunit

ctgtAgctattggTacctgcgcAgcTtatggtggcatcccAgctgcAgctggAaatgtta    4980
gacaTcgataaccAtggacgcgTcgAataccaccgtagggTcgacgTcgaccTttacaat
T  V  A  I  G  T  C  A  A  Y  G  G  I  P  A  A  A  G  N  V
(in frame with TEV site)
small hydrogenase subunit

cgggctctaagtcagtgcgtgatttctttgccgaagaaaaaatcgagaagctgctggtaa    5040
gcccgagattcagtcacgcactaaagaaacggcttctttttttagctcttcgacgaccatt
T  G  S  K  S  V  R  D  F  F  A  E  E  K  I  E  K  L  L  V
(in frame with TEV site)
small hydrogenase subunit

acgtgccAggttgtcctcctcacccAgattggatggtaggtacgttagttgcggcatggt    5100
tgcacggTccaacaggaggagtgggTctaacctaccatccatgcaatcaacgccgtacca
N  V  P  G  C  P  P  H  P  D  W  M  V  G  T  L  V  A  A  W
(in frame with TEV site)
small hydrogenase subunit
```

```
ctcacgttctgaacccgactgaacatcctttacccgagttggatgatgatggccgtccgt   5160
gagtgcaagacttgggctgacttgtaggaaatgggctcaacctactactaccggcaggca
S  H  V  L  N  P  T  E  H  P  L  P  E  L  D  D  D  G  R  P
(in frame with TEV site)
small hydrogenase subunit

tgcttttCttCggcgataacatccatgaGaactgtccttaCctggaCaagtacgataaCa   5220
acgaaaaGaaGccgctattgtaggtactCttgacaggaatGgacctGttcatgctattGt
L  L  F  F  G  D  N  I  H  E  N  C  P  Y  L  D  K  Y  D  N
(in frame with TEV site)
small hydrogenase subunit

gtgaatttgcagagacctttaccaaacccggttgcaaagcagaattgggatgcaaaggac   5280
cacttaaacgtctctggaaatggtttgggccaacgtttcgtcttaaccctacgtttcctg
S  E  F  A  E  T  F  T  K  P  G  C  K  A  E  L  G  C  K  G
(in frame with TEV site)
small hydrogenase subunit

cctccacgtacgcggactgcgctaagcgtcgttggaataacggtatcaattggtgtgtag   5340
ggaggtgcatgcgcctgacgcgattcgcagcaaccttattgccatagttaaccacacatc
P  S  T  Y  A  D  C  A  K  R  R  W  N  N  G  I  N  W  C  V
(in frame with TEV site)
small hydrogenase subunit

aaaacgcagtgtgcattggttgtgtcgagcccgacttcccagacgggaagtcgccatttt   5400
ttttgcgtcacacgtaaccaacacagctcgggctgaagggtctgcccttcagcggtaaaa
E  N  A  V  C  I  G  C  V  E  P  D  F  P  D  G  K  S  P  F
(in frame with TEV site)
small hydrogenase subunit

acgttgcagagggaggaaaagaaATGAGTCAGGCTGCAACACCCGCTGCCGACGGTAAAG   5460
tgcaacgtctccctccttttcttTACTCAGTCCGACGTTGTGGGCGACGGCTGCCATTTC
Y  V  A  E  G  G  K  E  M  S  Q  A  A  T  P  A  A  D  G  K
(in frame with TEV site)
small hydro...  RBS  truncated large hydrogenase subunit (U493C)

TTAAGATCTCGATCGATCCATTGACTCGTGTCGAGGGTCACCTTAAGATCGAAGTAGAGG   5520
AATTCTAGAGCTAGCTAGGTAACTGAGCACAGCTCCCAGTGGAATTCTAGCTTCATCTCC
V  K  I  S  I  D  P  L  T  R  V  E  G  H  L  K  I  E  V  E
(in frame with TEV site)
truncated large hydrogenase subunit (U493C)

TAAAGGATGGAAAGGTGGTAGACGCTAAGTGCAGTGGTGGTATGTTTCGTGGCTTCGAGC   5580
ATTTCCTACCTTTCCACCATCTGCGATTCACGTCACCACCATACAAAGCACCGAAGCTCG
V  K  D  G  K  V  V  D  A  K  C  S  G  G  M  F  R  G  F  E
(in frame with TEV site)
truncated large hydrogenase subunit (U493C)
```

AGATCTTGCGTGGTCGTGACCCTCGTGATTCGTCACAGATTGTTCAGCGCATCTGTGGCG 5640
TCTAGAACGCACCAGCACTGGGAGCACTAAGCAGTGTCTAACAAGTCGCGTAGACACCGC
Q I L R G R D P R D S S Q I V Q R I C G
(in frame with TEV site)
truncated large hydrogenase subunit (U493C)

TTTGTCCAACAGCGCACTGCACGGCATCCGTCATGGCGCAAGATGACGCATTTGGAGTGA 5700
AAACAGGTTGTCGCGTGACGTGCCGTAGGCAGTACCGCGTTCTACTGCGTAAACCTCACT
V C P T A H C T A S V M A Q D D A F G V
(in frame with TEV site)
truncated large hydrogenase subunit (U493C)

AAGTCACAACGAACGGGCGCATTACGCGTAACCTGATCTTCGGAGCTAACTACTTGCAGA 5760
TTCAGTGTTGCTTGCCCGCGTAATGCGCATTGGACTAGAAGCCTCGATTGATGAACGTCT
K V T T N G R I T R N L I F G A N Y L Q
(in frame with TEV site)
truncated large hydrogenase subunit (U493C)

GTCACATCCTGCACTTCTACCATCTTGCAGCTCTGGATTATGTCAAAGGTCCTGACGTGT 5820
CAGTGTAGGACGTGAAGATGGTAGAACGTCGAGACCTAATACAGTTTCCAGGACTGCACA
S H I L H F Y H L A A L D Y V K G P D V
(in frame with TEV site)
truncated large hydrogenase subunit (U493C)

CTCCATTCGTACCACGCTATGCCAACGCGGACCTTTTGACTGACCGTATCAAGGATGGCG 5880
GAGGTAAGCATGGTGCGATACGGTTGCGCCTGGAAAACTGACTGGCATAGTTCCTACCGC
S P F V P R Y A N A D L L T D R I K D G
(in frame with TEV site)
truncated large hydrogenase subunit (U493C)

CAAAGGCTGATGCCACCAACACGTATGGACTGAATCAGTACTTGAAGGCCTTGGAGATCC 5940
GTTTCCGACTACGGTGGTTGTGCATACCTGACTTAGTCATGAACTTCCGGAACCTCTAGG
A K A D A T N T Y G L N Q Y L K A L E I
(in frame with TEV site)
truncated large hydrogenase subunit (U493C)

GTCGCATTTGCCACGAAATGGTCGCTATGTTTGGTGGTCGTATGCCACATGTACAAGGTA 6000
CAGCGTAAACGGTGCTTTACCAGCGATACAAACCACCAGCATACGGTGTACATGTTCCAT
R R I C H E M V A M F G G R M P H V Q G
(in frame with TEV site)
truncated large hydrogenase subunit (U493C)

TGGTTGTAGGAGGTGCTACAGAAATTCCCACAGCGGATAAGGTCGCAGAGTACGCAGCTC 6060
ACCAACATCCTCCACGATGTCTTTAAGGGTGTCGCCTATTCCAGCGTCTCATGCGTCGAG
M V V G G A T E I P T A D K V A E Y A A
(in frame with TEV site)
truncated large hydrogenase subunit (U493C)

GCTTCAAAGAGGTCCAAAAGTTCGTCATCGAGGAATACTTGCCTTTGATCTACACACTTG 6120
CGAAGTTTCTCCAGGTTTTCAAGCAGTAGCTCCTTATGAACGGAAACTAGATGTGTGAAC
R F K E V Q K F V I E E Y L P L I Y T L
(in frame with TEV site)
truncated large hydrogenase subunit (U493C)

GATCGGTTTACACTGACCTTTTCGAGACCGGTATTGGATGGAAGAACGTCATCGCTTTCG 6180
CTAGCCAAATGTGACTGGAAAAGCTCTGGCCATAACCTACCTTCTTGCAGTAGCGAAAGC
G S V Y T D L F E T G I G W K N V I A F
(in frame with TEV site)
truncated large hydrogenase subunit (U493C)

GTGTGTTCCCAGAGGACGACGATTACAAGACTTTCTTGTTGAAACCGGGTGTCTACATCG 6240
CACACAAGGGTCTCCTGCTGCTAATGTTCTGAAAGAACAACTTTGGCCCACAGATGTAGC
G V F P E D D D Y K T F L L K P G V Y I
(in frame with TEV site)
truncated large hydrogenase subunit (U493C)

ACGGTAAGGACGAGGAGTTCGACAGCAAACTTGTTAAGGAATACGTTGGTCACTCCTTTT 6300
TGCCATTCCTGCTCCTCAAGCTGTCGTTTGAACAATTCCTTATGCAACCAGTGAGGAAAA
D G K D E E F D S K L V K E Y V G H S F
(in frame with TEV site)
truncated large hydrogenase subunit (U493C)

TCGATCATAGTGCCCCAGGAGGGCTGCACTATAGCGTCGGGGAGACAAACCCTAATCCTG 6360
AGCTAGTATCACGGGGTCCTCCCGACGTGATATCGCAGCCCCTCTGTTTGGGATTAGGAC
F D H S A P G G L H Y S V G E T N P N P
(in frame with TEV site)
truncated large hydrogenase subunit (U493C)

ACAAACCTGGCGCCTATTCATTTGTAAAAGCACCCCGTTACAAGGATAAACCGTGCGAGG 6420
TGTTTGGACCGCGGATAAGTAAACATTTTCGTGGGGCAATGTTCCTATTTGGCACGCTCC
D K P G A Y S F V K A P R Y K D K P C E
(in frame with TEV site)
truncated large hydrogenase subunit (U493C)

TAGGGCCTTTAGCTCGTATGTGGGTCCAAAACCCGGAGTTGTCACCTGTGGGGCAGAAAC 6480
ATCCCGGAAATCGAGCATACACCCAGGTTTTGGGCCTCAACAGTGGACACCCCGTCTTTG
V G P L A R M W V Q N P E L S P V G Q K
(in frame with TEV site)
truncated large hydrogenase subunit (U493C)

TGTTAAAAGAACTTTATGGAATTGAAGCCAAGAACTTTCGCGATCTTGGCGACAAAGCAT 6540
ACAATTTTCTTGAAATACCTTAACTTCGGTTCTTGAAAGCGCTAGAACCGCTGTTTCGTA
L L K E L Y G I E A K N F R D L G D K A
(in frame with TEV site)
truncated large hydrogenase subunit (U493C)

TCTCTATCATGGGCCGTCATGTAGCACGTGCTGAAGAGACCTGGTTGACTGCGGTCGCAG 6600
AGAGATAGTACCCGGCAGTACATCGTGCACGACTTCTCTGGACCAACTGACGCCAGCGTC
F S I M G R H V A R A E E T W L T A V A
(in frame with TEV site)
truncated large hydrogenase subunit (U493C)

TGGAGAAGTGGTTAAAGCAGGTCCAGCCTGGTGCGGAGACTTATGTCAAGAGCGAAATTC 6660
ACCTCTTCACCAATTTCGTCCAGGTCGGACCACGCCTCTGAATACAGTTCTCGCTTTAAG
V E K W L K Q V Q P G A E T Y V K S E I
(in frame with TEV site)
truncated large hydrogenase subunit (U493C)

CGGACGCAGCAGAGGGTACCGGTTTCACAGAAGCGCCTCGTGGAGCGCTGCTGCACTACT 6720
GCCTGCGTCGTCTCCCATGGCCAAAGTGTCTTCGCGGAGCACCTCGCGACGACGTGATGA
P D A A E G T G F T E A P R G A L L H Y
(in frame with TEV site)
truncated large hydrogenase subunit (U493C)

TGAAGATCAAGGATAAAAAGATCGAGAACTACCAAATTGTCTCAGCCACACTGTGGAATG 6780
ACTTCTAGTTCCTATTTTTCTAGCTCTTGATGGTTTAACAGAGTCGGTGTGACACCTTAC
L K I K D K K I E N Y Q I V S A T L W N
(in frame with TEV site)
truncated large hydrogenase subunit (U493C)

CTAACCCTCGCGATGATATGGGCCAGCGTGGACCAATTGAGGAGGCACTTATCGGTGTCC 6840
GATTGGGAGCGCTACTATACCCGGTCGCACCTGGTTAACTCCTCCGTGAATAGCCACAGG
A N P R D D M G Q R G P I E E A L I G V
(in frame with TEV site)
truncated large hydrogenase subunit (U493C)

CAGTGCCTGATATCAAGAACCCAGTGAACGTGGGTCGTTTGGTTCGCTCGTATGACCCAT 6900
GTCACGGACTATAGTTCTTGGGTCACTTGCACCCAGCAAACCAAGCGAGCATACTGGGTA
P V P D I K N P V N V G R L V R S Y D P
(in frame with TEV site)
truncated large hydrogenase subunit (U493C)

GCCTGGGCTGTGCCGTGCACTAAATTGGAAGTGGATAACGGATCCGAATTCGAGCTCCGT 6960
CGGACCCGACACGGCACGTGATTTAACCTTCACCTATTGCCTAGGCTTAAGCTCGAGGCA
C L G C A V H *
(in frame with TEV site)
truncated large hydrogenase subunit (U493C)

CGACAAGCTTGCGGCCGCACTCGAGCACCACCACCACCACCACTGAGATCCGGCTGCTAA 7020
GCTGTTCGAACGCCGGCGTGAGCTCGTGGTGGTGGTGGTGGTGACTCTAGGCCGACGATT

```
CAAAGCCCGAAAGGAAGCTGAGTTGGCTGCTGCCACCGCTGAGCAATAACTAGCATAACC
                                                                    7080
GTTTCGGGCTTTCCTTCGACTCAACCGACGACGGTGGCGACTCGTTATTGATCGTATTGG
                                                T7 terminator

CCTTGGGGCCTCTAAACGGGTCTTGAGGGGTTTTTTGCTGAAAGGAGGAACTATATCCGG
                                                                    7140
GGAACCCCGGAGATTTGCCCAGAACTCCCCAAAAAACGACTTTCCTCCTTGATATAGGCC
                 T7 terminator

AT   3'
    ...   7142
TA   5'
```

**Description:**	
**Created:**	niedziela, 2 maja 2021
**Last Modified:**	środa, 7 lipca 2021
**Accession Number:**	
**Code Number:**	
**Sequence Author:**	
**DNA Type:**	Synthetic DNA
**Laboratory Host Organism:**	
**Bacterial Transformation Strain:**	Unspecified<br>Dam^+ Dcm^+ $EcoKI^+$
**Comments:**	
**References:**	
